# On‐site forensic analysis of colored seized materials: Detection of brown heroin and MDMA‐tablets by a portable NIR spectrometer

**DOI:** 10.1002/dta.3356

**Published:** 2022-08-31

**Authors:** Ruben F. Kranenburg, Henk‐Jan Ramaker, Arian C. van Asten

**Affiliations:** ^1^ Unit Amsterdam, Forensic Laboratory Dutch National Police Amsterdam The Netherlands; ^2^ Van't Hoff Institute for Molecular Sciences University of Amsterdam Amsterdam GD The Netherlands; ^3^ TIPb Amsterdam The Netherlands; ^4^ Co van Ledden Hulsebosch Center (CLHC), Amsterdam Center for Forensic Science and Medicine Amsterdam The Netherlands

**Keywords:** colored samples, forensic casework analysis, illicit drug analysis, near‐infrared spectroscopy, portable devices

## Abstract

The increasing workload for forensic laboratories and the expanding complexity of the drug market necessitates efficient approaches to detect drugs of abuse. Identification directly at the scene of crime enables investigative forces to make rapid decisions. Additionally, on‐site identification of the material also leads to considerable efficiency and cost benefits. As such, paperwork, transportation, and time‐consuming analysis in a laboratory may be avoided. Near‐infrared (NIR) spectroscopy is an analysis technique suitable for rapid drug testing using portable equipment. A possible limitation of spectroscopic analysis concerns the complexity of seized materials. NIR measurements represent composite spectra for mixtures and diagnostic spectral features can be obscured by excipients such as colorants. Herein, a NIR‐based (1300–2600 nm) detection of heroin and MDMA in colored casework (i.e., brown powders and ecstasy tablets) using a portable analyzer is presented. The application includes a multistage data analysis model based on the net analyte signal (NAS) approach. This identification model was specifically designed for mixture analysis and requires a limited set of pure reference spectra only. Consequently, model calibration efforts are reduced to a minimum. A total of 549 forensic samples was tested comprising brown heroine samples and a variety of colored tablets with different active ingredients. This investigation led to a >99% true negative and >93% true positive rate for heroin and MDMA. These results show that accurate on‐site detection in colored casework is possible using NIR spectroscopy combined with an efficient data analysis model. These findings may eventually help in the transition of routine forensic laboratories from laboratory‐based techniques to portable equipment operated on scene.

## Graphical abstract

Near‐infrared (NIR) spectroscopy is an analysis technique suitable for rapid drug testing using portable equipment. Herein, a NIR‐based (1300–2600 nm) detection of heroin and MDMA in colored casework (i.e., brown powders and ecstasy tablets) using a portable analyzer is presented. The identification model was specifically designed for mixture analysis and requires a limited set of pure reference spectra only. Investigation of 549 forensic samples led to a >99% true negative and >93% true positive rate for heroin and MDMA.

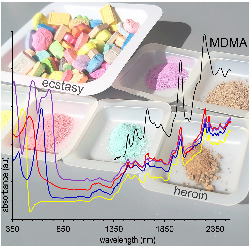

## 1 INTRODUCTION

Opioid drugs (such as heroin) and amphetamine‐type stimulants (such as MDMA) are among the most produced, traded, and consumed drug categories worldwide.^1^ Unlike cocaine that typically has an appearance as a white or lightly colored powder, heroin street samples are generally of a beige, brown, or dark brown complexion. Ecstasy, a regular formulation of MDMA, appears as tablets in a wide range of different and often exuberant colors.

Although both crude heroin and MDMA may have a brown color due to impurities originating from the manufacturing process, the color of street samples in most cases originates from deliberately added colorants. Paracetamol and caffeine are the most commonly used adulterants in heroin. These adulterants are white powders in their pure form. To mask dilution, paracetamol–caffeine mixtures itself are often given a brown color by the use of dyes.^2^ Synthetic colorants brilliant black (E151), sunset yellow (E110), and tartrazine (E102) have been identified in brown heroin casework samples.^3^ Ecstasy tablets have a large variety of shapes, imprints, and colors to make them aesthetically appealing. Common commercially available food colorants such as azo dyes are regularly used for this purpose.^4^, ^5^

Forensic drug testing laboratories are demanding reliable methods for rapid on‐scene detection of these illicit substances in suspected casework materials. Traditionally, colorimetric spot tests are used for this purpose although their applicability is only limited to substances for which such a test is available. Additionally, these tests require a chemical reaction with the suspected material that often involves strong acids and thus may pose a safety risk when performed on‐site.^6^ In recent years, on‐site drug detection by portable spectroscopic techniques such as Raman,^7^, ^8^ Fourier transform infrared (FTIR),^9^ and near‐infrared (NIR) became readily accessible and were also used for casework analysis in the forensic field.^10^, ^11^ NIR is a powerful noninvasive technique for fast and efficient substance detection due to the availability of small size instrumentation (handheld, pocket size, smartphone sensor^12^) at relatively low cost.^13^, ^14^ Recent applications of NIR detection in the forensic drug testing field include the detection of cocaine,^15^, ^16^, ^17^, ^18^, ^19^ “legal high” designer drugs,^20^ ketamine, heroin, and methamphetamine.^15^ All these implementations mainly focus on lightly colored (i.e., white) powdered samples.

Eliaerts *et al* in 2021 reported on the challenges that colored samples may pose in forensic drug detection by dyes or pigments obscuring diagnostic spectroscopic signals.^21^ A technique successfully applied in the on‐site analysis of colored samples is electrochemical detection. Because this technology is based on oxidation or reduction characteristics instead of light absorption, it is independent of the color of the sample material.^22^ This way, detection of MDMA^23^ and heroin^24^ is possible irrespective of sample shade. In line with the color tests, a drawback of electrochemical detection is the required subsampling and the application of chemicals. Additionally, disposables as single‐use electrodes are needed.

Only a limited number of studies are available demonstrating NIR‐based detection of illicit substances in colored samples. Coppey *et al* ^18^ included examples of heroin in their study on a MicroNIR‐based analytical platform for drug detection. This same group also reported on cannabinoid detection in cannabis plant material.^18^, ^25^ The group of Materazzi reported on NIR‐based detection of the synthetic cannabimimetic AKB‐48 in the herbal matrices mint, eucalyptus, fennel, and sage^26^ as well as cannabinoid detection directly in hemp seed oil,^27^ hemp flours,^28^ and veterinary feeds from vegetal materials.^29^ All these studies were carried out using MicroNIR handheld spectrometers operating in the 950–1650 nm wavelength range.

Chen *et al* ^30^ used a portable NIR spectrometer operating in the 908–1676 nm range for the detection of heroin in casework materials by using a partial least square (PLS) model based on a 380‐sample training set and a 30‐sample test set. They mentioned neither the salt form (HCl or base) nor the color of the casework material in their report. In addition to the works of Coppey *et al* ^18^ and Chen *et al*,^30^ the only other NIR‐based studies on heroin were performed on benchtop instruments by Moros *et al*.^31^, ^32^ This group developed a calibration model using data from a laboratory‐grade Bruker FT‐NIR (850–2750 nm). Their initial model used PLS based on the 1100–1647 nm region of interest.^31^ In a follow‐up study, they improved the performance by implementing an uninformative variable elimination approach as preselection for PLS.^32^ Additionally, they provided insight in the origin of diagnostic spectral peaks for heroin (in its base form).

In earlier work, our group reported the NIR spectrum of MDMA HCl in the implementation of a calibration‐friendly approach for mixture analysis in forensic samples that were predominantly white powders.^17^ Only a very limited number of studies reported on NIR‐based MDMA detection. In 1999, Sondermann and Kovar performed NIR analyses on both intact and pulverized tablets using a benchtop instrument operating in the 1100–2500 nm range. They showed that MDMA detection is possible using PLS modeling.^33^, ^34^, ^35^ It is noteworthy that the reported MDMA HCl spectrum in these studies was different from the spectra recorded by our group.^17^, ^36^ These differences were attributed to different hydrated and anhydrous polymorphous forms of MDMA HCl.^35^, ^37^ This indicates that the MDMA used in these studies was either dried to convert the hydrates into anhydrates or was synthesized in the clandestine laboratory in an anhydrous way. To our knowledge, only one earlier study has reported on MDMA detection in colored casework materials (i.e., ecstasy tablets) using portable NIR instruments. Tsujikawa *et al* ^37^ developed a library search‐based screening for MDMA using a portable 1400–2400 nm NIR spectrometer.

A plausible explanation for the lack of NIR‐based studies for MDMA or heroin detection is the perceived detrimental influence of the complex mixture matrix and appearance as, for example, tablets or chunks. Portable NIR spectrometers operating below ~1000 nm were generally found unsuitable for illicit drug detection in colored samples due to the major influence of colorants in this wavelength range^16^, ^36^ combined with a limited number of spectral features of MDMA.^36^ Both a wider and higher wavelength range of the NIR spectrum (above 1000 and up to 2600 nm) is suggested for MDMA detection due to specific spectral features in this range.^17^ Additionally, the spectral fingerprint obtained from forensic casework is a combination of signals from all constituents in the sample material. As adulterants (e.g., caffeine and paracetamol), impurities (e.g., papaverine and noscapine), and excipients (e.g., tablet fillers microcrystalline cellulose and lactose) are commonly present in casework and contribute to the spectral signal, advanced data processing tools and models are required for successful identification.

In this study, we present a novel application to detect common drugs of abuse in colored forensic samples using portable NIR technology, with a focus on heroin and MDMA. Based on the calibration‐free approach for mixture analysis introduced before and demonstrated for cocaine,^17^ we now successfully performed the detection of heroin and MDMA in 549 casework samples. The sample set used for validation of our application included, among others, 55 brown casework powders seized as heroin‐suspected material, 136 differently colored tablets, and 71 colored powders originating from crushed tablets.

## 2 MATERIALS AND METHODS

### 2.1 Forensic casework materials

A total of 549 forensic samples from different types and origin were used in this study. These samples provide a broad representation of materials that may be encountered in forensic casework. The composition of the individual samples was established by validated GC‐MS methods of the illicit drug laboratory of the Amsterdam police. The identities of all individual samples can be found in the supporting information ([Link], [Link], [Link]). The 549 forensic samples are categorized and grouped into the following subsets of data:

○ **Set H (heroin)**: This sample set consists of 55 brown‐colored powders from casework samples seized in Amsterdam in 2021 and 2022. The color of the individual samples includes shades of khaki, maroon, caramel, taupe, and dark brown. A total of 46 out of 55 samples were identified as heroin‐containing with GC‐MS. The remaining nine brown powders did not contain heroin. These samples were diverse in composition and comprised brown‐colored mixtures of paracetamol and caffeine, benzylmethylketone (BMK), and tobacco and instant cocoa powder.
○ **Set M (MDMA)**: This sample set consists of 11 different batches of MDMA crystals originating from various seizures in the Amsterdam area in 2019 and 2020. The crystals ranges in hue from cola brown via champagne to cream.
○ **Set T (intact tablets)**: A total of 71 different ecstasy tables in various shapes and colors, including shades of red, orange, blue, green, yellow, purple, and gray. Out of the 71 tablets, 39 were MDMA‐containing, and 32 contained other active ingredients (e.g., 2C‐B, 4‐FMA, and 4‐MMC). The tablets originated from 2020 casework of the Amsterdam police. Individual pictures, sizes, colors, and identities are reported in earlier work.^23^
○ **Set P (crushed tablets)**: This set consisted of a selection of intact tablets (another accompanying sample from Set T) that were crushed to better understand the effects of sample representation and heterogeneity on model performance
○ **Set T2 (extra intact tablets)**: A set of 65 ecstasy tablets with novel imprints or color (different from Set T) encountered in casework of the Amsterdam police for the first time in 2021. Out of these 65 tablets, 47 were MDMA‐containing, and 18 contained another active ingredient (2C‐B, 4‐FA, amphetamine).
○ **Set C,D,N (other drugs and substances)**: This set of 95 samples consisted of a wide range of substances that can be encountered in a forensic setting. The 17 samples coded “C” are common drugs of abuse, the 38 samples coded “D” are various synthetic designer drugs, and the 40 samples coded “N” are common pharmaceuticals, common household chemicals, and adulterants, both pure and in mixtures. The set “N” samples originated from various commercial sources reported elsewhere.^16^
○ **Set PAM (Police Amsterdam casework samples)**: A total of 181 samples with a light (white, off‐white, and cream) color were randomly selected from 2020 casework of the Amsterdam police. These samples were mainly cocaine (109 times). Other samples included ketamine, amphetamine, methamphetamine, MDMA powders, and various cutting agents. Additional details are published in earlier work.^17^

### 2.2 Reference matrix libraries

Matrices for both heroin and MDMA were created to load the identification models. Each matrix consists of preselected library components. A library component represents an NIR spectrum of a pure compound such as paracetamol. Library components become a matrix member because they can be encountered in casework samples. The heroin matrix consisted of the following library components: heroin HCl·H_2_O, heroin base, acetylcodeine, caffeine, noscapine HCl·H_2_O, papaverine HCl, and paracetamol. The MDMA matrices consisted of the following library components: MDMA HCl, microcrystalline cellulose (Avicel™), lactose, mannitol, *myo*‐inositol, magnesium stearate, and 2C‐B. For MDMA HCl, both spectra from the neat material (own synthesis, identity confirmed by GC‐MS, FTIR, and NMR) being the common crystalline MDMA HCl hydrate as well as the anhydrous MDMA HCl form were recorded. Anhydrous MDMA HCl was prepared from MDMA HCl hydrate by drying grinded powder at 100°C for 2 h. Identity of the dried material was confirmed by GC‐MS and FTIR. Two separate MDMA matrices were created and assessed in this study: one with only the MDMA HCl hydrate spectrum and one with both the spectra of the hydrate and anhydrous form. Heroin base was prepared from heroin HCl (Slotervaartziekenhuis, The Netherlands) by alkaline extraction of an aqueous heroin HCl solution with diethyl ether. The organic phase was evaporated to dryness to obtain the heroin base. Its identity was confirmed with GC‐MS. No degradation products (monoacetylmorphine, morphine) were observed.

### 2.3 Instrumentation and software

All NIR measurements were carried out on the Powder Puck. The hardware and data analysis software for forensic casework were developed for a previous study on cocaine samples and are described in more detail in an earlier report.^17^ In short, the Powder Puck is a USB‐controlled portable spectrometer that consists of a NeoSpectra sensor (Si‐WARE, Cairo, Egypt). This sensor operates in the 1300–2600 nm range with a spectral resolution of 16 nm at 1550 nm. The raw spectral data (160 datapoints) were processed by an in‐house developed MATLAB (Version 2020b Update 5) application: Second derivative spectra were projected onto a linear discriminant analysis (LDA) model based on a specifically selected matrix. The outcome of the LDA model serves as input to an optional subsequent mixture detection analysis using a NAS approach. The NAS model determines the optimal combination of library components that describes the unknown sample spectra best. The outcome of the software presents the user with a possible composition of the samples (either as a pure component or a mixture of components). Each identity result is accompanied with a similarity score that ranges between 0.00 and 1.00. A score of 1.00 indicates a match with a pure reference substance in the LDA model. A score between 0.00 and 0.99 originates from the subsequent NAS model and is a measure of the similarity between the recorded spectrum and the model result (calculated optimal fit with a substance or mixture at a given concentration). Herein, a higher score indicates a better similarity. For this study, a score above 0.80 is considered a confident identification by the software. A similarity score above 0.70 and **≤**0.80 serves merely as an indication of the potential presence of a drug of abuse. Consequently, the corresponding sample could be sent to the laboratory for confirmatory analysis. Similarity scores **≤**0.70 imply that no substance(s) from the given matrix was selected for identification purposes. This, therefore, is considered to be a negative result (no substance detected). For the purpose of this study, identifications with similarity scores >0.70 and **≤**0.80 are also treated as negatives. Consequently, these outcomes are rated either as false or true negatives. It must be noted that these threshold levels were set by practical experience and may be further optimized for specific applications. A higher threshold will, for example, lead to less false positive results but may come with an increase of false negative results.

Casework samples were scanned in triplicate with the exception of the seized tablets in set T2, which were only scanned once. Reference spectra of all substances included in the matrix libraries of heroin and MDMA were scanned 15 times per individual component. All samples were analyzed in glass vials by positioning the glass vial on top of the scanner and recording absorbance data in diffuse reflection (i.e., remission) mode. Samples were shaken between each replicate. Background measurements were performed every 15 min during measurement sessions using Fluorilon background material.

## 3 RESULTS AND DISCUSSION

### 3.1 NIR spectral selectivity of heroin and MDMA

**FIGURE 1:**
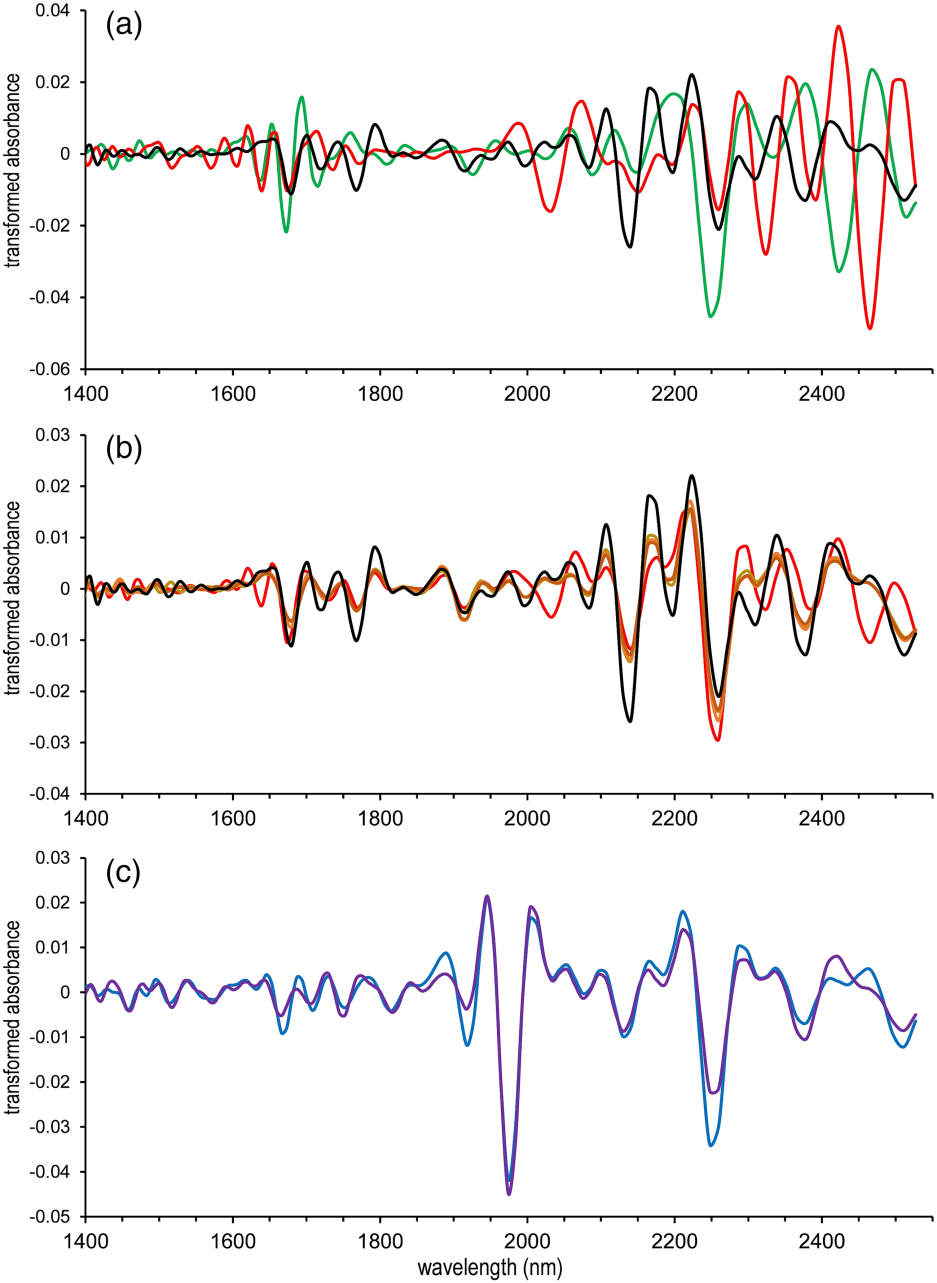
Second derivative NIR spectra of heroin base (black trace in a and b) compared with common adulterants (a) and various brown heroin casework samples (b). (a) Paracetamol (red), caffeine (green). (b) Brown heroin samples (brown to orange shades), brown heroin sample highly adulterated with paracetamol and caffeine, H19 (red). (c) Heroin HCl·H2O (purple) and a white heroin casework sample, H8 (blue) [Colour figure can be viewed at wileyonlinelibrary.com]

The possibilities for heroin and MDMA detection in (colored) forensic casework by NIR spectroscopy were examined. As a first step, the associated NIR spectra and unique spectral features were visually inspected and compared with other relevant substances. Earlier studies already showed NIR spectra of MDMA and heroin, both yielding sufficient differences from other common drugs of abuse (e.g., cocaine, ketamine, and methamphetamine) to allow for their differentiation.^17^, ^36^ Similarly to cocaine, heroin may also occur in both its HCl salt or free base form in casework samples; typical brown heroin samples are reported to predominantly contain heroin base, whereas the more pure “white” heroin typically contains heroine HCl.^38^ Unlike chromatographic methods (e.g., GC‐MS) where samples are dissolved and converted into their native forms, direct spectroscopic analyses produce different spectra for different salt forms.^7^, ^39^ It is therefore important to include both heroin HCl and heroin base in the spectral library. Figure 1a shows the second derivative spectrum of heroin base compared with its two main cutting agents paracetamol (red trace) and caffeine (green trace) clearly showing differences (e.g., at ~1800 and ~2150 nm). Figure 1b shows the heroin base (black) reference spectrum in overlay with four brown heroin casework samples. In three of these samples (brown to orange shades), the spectral features diagnostic for heroin can clearly be observed. The spectrum of the forth sample (red plot)—brown heroin sample H19 that is highly adulterated with paracetamol and caffein—clearly shows spectral features of the two adulterants (e.g., valley ~2450 nm for paracetamol and peak ~2300 nm for both paracetamol and caffeine) in combination with partly obscured signals for heroin. Figure 1c depicts the reference heroin HCl·H_2_O (purple) in overlay with a seized white heroin samples (blue) clearly showing spectral similarities. These finding show, in line with NIR studies on cocaine,^16^, ^17^ that spectra from heroin casework samples consist of spectral features originating from both the active ingredient and the adulterants present. The latter may manifest in a wide range in terms of active ingredient concentration and composition, thus complicating data analysis. To tackle these complications, the advanced data analysis approach previously implemented in the Powder Puck software for casework cocaine mixtures was also applied for the current work.^17^ Because this approach focuses on mixture deconvolution, it is also expected to be suitable for heroin and MDMA casework. For comparative purposes, the unprocessed raw spectra of all plots in Figure 1 are shown in Figure S1.

**FIGURE 2:**
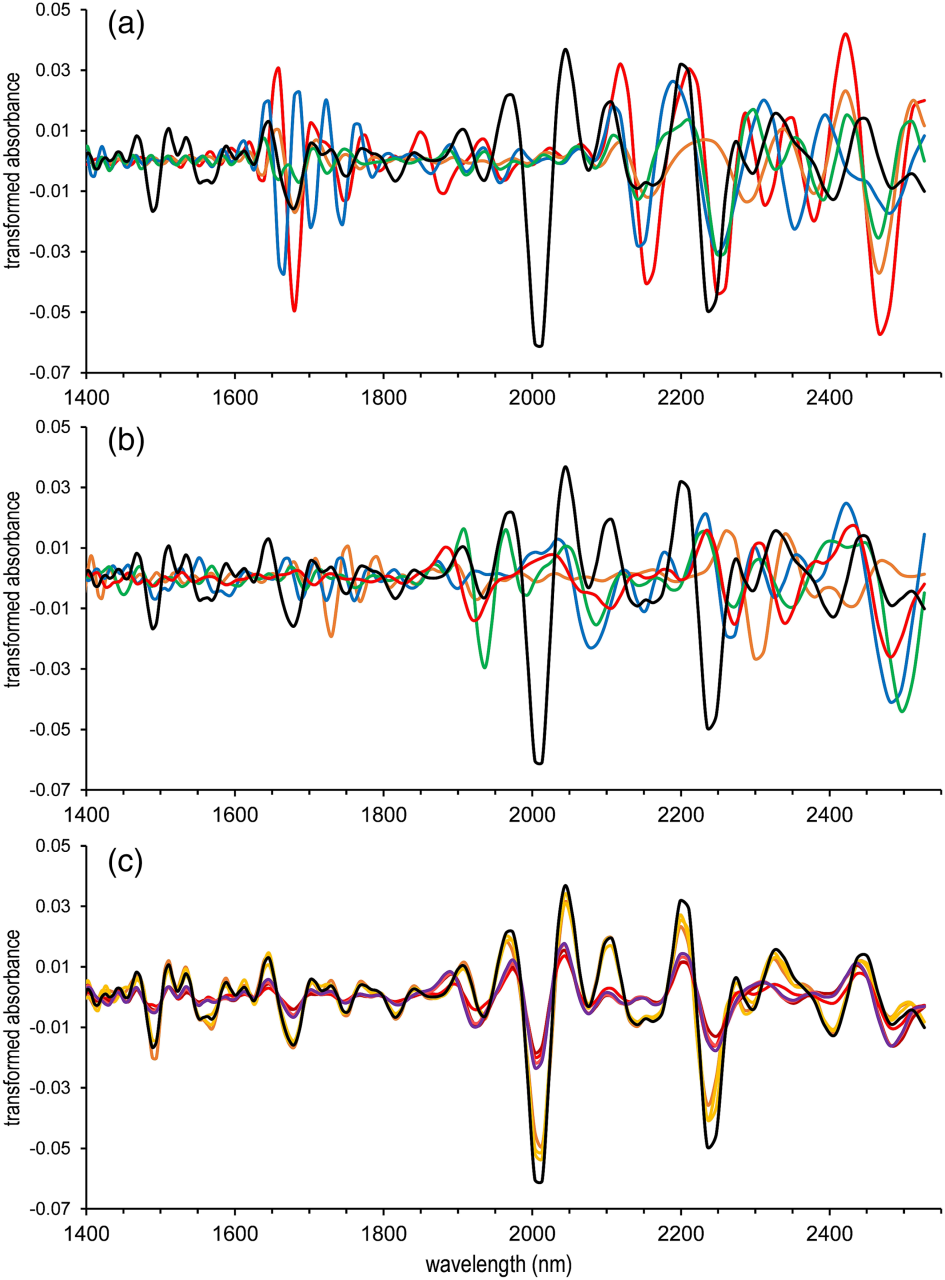
Second derivative NIR spectra of MDMA HCl (black trace in all panels) compared with other drugs (a), common excipients (b), and various MDMA‐containing casework samples (c). (a) Methamphetamine HCl (red), cocaine HCl (green), ketamine (blue), amphetamine sulfate (orange). (b) Microcrystalline cellulose (red), lactose (green), mannitol (blue), Mg stearate (orange). (c) MDMA crystals, M1‐3 (orange shades), crushed MDMA‐containing ecstasy tablets, P1‐4 (purple to red shades) [Colour figure can be viewed at wileyonlinelibrary.com]

**FIGURE 3:**
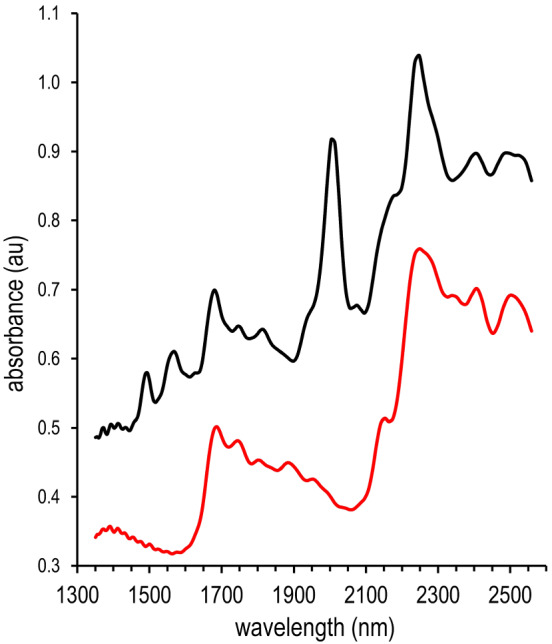
Normalized (raw) diffuse reflection NIR spectra of hydrated MDMA HCl crystals (black trace) and anhydrous MDMA HCl (red trace) [Colour figure can be viewed at wileyonlinelibrary.com]

Second derivative spectra of MDMA HCl also show significant differences among other drugs (Figure 2a), excipients and adulterants commonly encountered in ecstasy tablets (Figure 2b). The most notable differences are the ~2000 nm valley and adjacent peaks in the derivative spectra. These features originate from a major peak at 2020 nm in the MDMA HCl raw spectrum (Figure 3, black trace). The diagnostic value of this peak—as well as the peaks around 1500 nm—becomes evident when the reference MDMA spectrum is compared with MDMA‐containing casework. Figure 2c shows an overlay of MDMA HCl (black) with three different batches of seized MDMA crystals (plots in orange shades) as well as four different crushed MDMA‐containing ecstasy tablets (plots in purple to red shades). The latter show less abundant signals although the MDMA‐specific features (~1500 nm and ~2000 nm) are still clearly observable. Additionally, spectral features from the common tablet filler microcrystalline cellulose (Figure 2b, red plot) also contribute to the spectra of the ecstasy tablets such as the ~1875 nm peak, the ~1925 nm valley, and the valley at ~2350 nm. Similar to heroin, this phenomenon shows that a data analysis approach focusing on mixture detection and spectral deconvolution may also be suitable for MDMA detection in forensic casework. Again, the raw spectra of all plots in Figure 2 can be found in Figure S2.

Unlike heroin, the free base form of MDMA is not a relevant issue in forensic casework as this substance is in liquid state at ambient temperatures. Spectral variation caused by hydration polymorphism may however complicate NIR identification similarly as in FTIR.^40^ To provide insight in the possible challenge due to this phenomenon, anhydrous MDMA HCl was prepared and analyzed (Figure 3). A comprehensive study on the occurrence of anhydrous MDMA HCl is currently in progress; however, preliminary results indicate that casework MDMA in the Netherlands almost exclusively consists of hydrated MDMA HCl·H_2_O crystals. Because both MDMA HCl types (anhydrous and hydrates) yield significantly different NIR spectra (Figure 3), both variants were included in a dedicated spectral reference library in this study.

### 3.2 Data analysis by the Powder Puck chemometric model

All 1516 spectra obtained from the 549 different forensic casework samples of the sample sets described in Section 2.1 were processed by the multistage chemometric model incorporated in the Powder Puck software.^17^ One of the features of this software is the availability of user‐selectable matrices for efficient analysis. This way, the user can select the most appropriate matrix based on their forensic expertise for a first analysis. For example, for white powdered samples, the cocaine matrix may be a first start, whereas for seized ecstasy tablets, an MDMA matrix may be profitable. In this study, two new matrices were developed, namely, the heroin matrix and the MDMA matrix. Both matrices consist of adulterants and excipients that are most likely to be encountered in casework samples. Expert knowledge about the Dutch illicit drugs market is utilized to select the proper library components. It must be remarked that manual selection of matrix libraries currently may be considered a constraining factor by end users. Automized processing of multiple matrices is therefore a desirable future development for this approach. This development is currently under investigation in our group. The composition of a matrix is crucial to correctly identify unknown samples based on their NIR spectrum. Each matrix composition is described in Section 2.2. Regarding MDMA, two different versions of the MDMA matrix were constructed: One matrix included both the anhydrate and hydrate forms of MDMA HCl. Another matrix was constructed that only included the hydrated form of MDMA HCl.

The identification result provided by the Powder Puck software contains three types of information: (i) identification, (ii) similarity, and (iii) indicative concentration. For example, sample H3 was identified first using the heroin matrix. The Powder Puck software returns the results as “Heroine Base (26%) + Paracetamol (34%) + Caffeine (17%)” with a similarity score of 0.92. When the MDMA matrix is selected to identify sample H3, the results read as “Inconclusive,” meaning that no match above the 0.70 threshold was found. Each individual scan result was identified on each of the three matrices. The results of this analysis are found in [Link], [Link], [Link]. An overview of the results per sample set is shown in Table 1. Herein, a true positive is considered a result with a similarity score >0.80 that includes (optionally among others) the drug‐of‐abuse component present in the sample. All samples were analyzed in triplicate, except for the tablets in set T2 that were only scanned once. The results of Table 1 can thus also be presented on the sample level; these figures can be found in Table S1.

**TABLE 1:** Performance of the chemometric model on the various sample sets and results in scans

Sample set	pos#	TP	FN	neg#	TN	FP
	Heroin matrix library					
H	138	128	10^a^	27	27	0
M	0	0	0	33	33	0
P	0	0	0	213	213	0
T	0	0	0	213	213	0
T2	0	0	0	65	65	0
C,D,N	9	9	0	276	276	0
PAM	0	0	0	542	542	0
*Total*	*147*	*137*	*10*	*1369*	*1369*	*0*

	MDMA matrix library (incl. anhydride)					
H	0	0	0	165	164	1
M	33	33	0	0	0	0
P	117	112	5^b^	96	96	0
T	117	111	6	96	96	0
T2	47	44	3	18	18	0
C,D,N	9	9	0	276	273	3
PAM	39	39	0	503	500	3
*Total*	*362*	*348*	*14*	*1154*	*1147*	*7*

	MDMA matrix library (excl. anhydride)					
H	0	0	0	165	163	2
M	33	33	0	0	0	0
P	117	112	5^b^	96	96	0
T	117	111	6	96	96	0
T2	47	44	3	18	18	0
C,D,N	9	9	0	276	276	0
PAM	39	39	0	503	503	0
*Total*	*362*	*348*	*14*	*1154*	*1152*	*2*

^a^ For 7 scans originating from three samples in set H, a match score between 0.70 and 0.80 for heroin base was provided by the software. These are considered false negatives in this study.

^b^ For two scans originating from one sample in set P, a match score between 0.70 and 0.80 for MDMA was provided by the software. These are considered false negatives in this study.

It must be noted all samples included in sets H, P, T, and T2 were diversely colored (including all regular hues) and that the samples in set M (MDMA crystals) ranged in color from cream to dark brown. The purpose of sets C,D,N and PAM (predominantly white and off‐white powders) was to assess the selectivity of the model against a wide variety of substances that may be encountered in a forensic setting.

#### 3.2.1 Heroin matrix library results

When looking at the heroin results, the most notable result is the total absence of false positives. Especially in forensic settings, a low false positive rate is important as this can have major adverse effects such as unjustified custody. Additionally, for a total of 10 scans, the presence of heroin was missed in a sample, thus resulting in a false negative. For seven out of these 10 scans, an indication for the presence of heroin was still given due to a heroin match with a similarity score between 0.70 and 0.80. The remaining three false negatives were the three replicates of sample H17, and for each scan, the sample was identified as a mixture of (only) paracetamol and caffeine. The GC‐MS analysis revealed that this particular sample had a low heroin content in addition to larger quantities of paracetamol and caffein. Additionally, this sample contained small quantities of 6‐monoacetylmorphine (6‐MAM), a substance that was lacking in the matrix library due to the unavailability of a reference standard. All other 43 brown‐colored powders were correctly identified as heroin‐containing. It is noteworthy that in all these cases, heroin base was detected. The only instances in which heroin HCl was detected were “white heroin” samples with a pale (cream, off‐white) color, namely, samples H8 and N23. The overall performance of the heroin matrix library is shown in Table 2, both in results per scan and results per sample. Individual sample results were determined by majority voting of the replicate scans. Generally, the performance of the heroin matrix library was more than satisfactory with a 93.2% true positive rate (6.8% false negative rate) and a 100% true negative rate (0% false positive rate).

**TABLE 2:** Overall performance of heroin and MDMA detection in forensic casework

Matrix library	pos#	TP	FN	neg#	TN	FP
	Scans					
Heroin	147	137 (93.2%)	10 (6.8%)^a^	1369	1369 (100%)	0 (0%)
MDMA (incl. anhydride)	362	348 (96.1%)	14 (3.9%)^b^	1154	1147 (99.4%)	7 (0.6%)
MDMA (excl. anhydride)	362	348 (96.1%)	14 (3.9%)^b^	1154	1152 (99.8%)	2 (0.2%)

	Samples					
Heroin	49	45 (91.8%)	4 (8.2%)^a^	500	500 (100%)	0 (0%)
MDMA (incl. anhydride)	152	145 (95.4%)	7 (4.6%)^b^	397	395 (99.5%)	2 (0.5%)
MDMA (excl. anhydride)	152	145 (95.4%)	7 (4.6%)^b^	397	396 (99.7%)	1 (0.3%)

^a^ For seven out of the 10 false negative scans and three out of the four false negative samples, an indication for the presence of heroin (match between 0.70 and 0.80) was provided by the software.

^b^ For two out of the 14 false negative scans and one out of the seven false negative samples, an indication for the presence of MDMA (match between 0.70 and 0.80) was provided by the software.

#### 3.2.2 MDMA matrix libraries results

**FIGURE 4:**
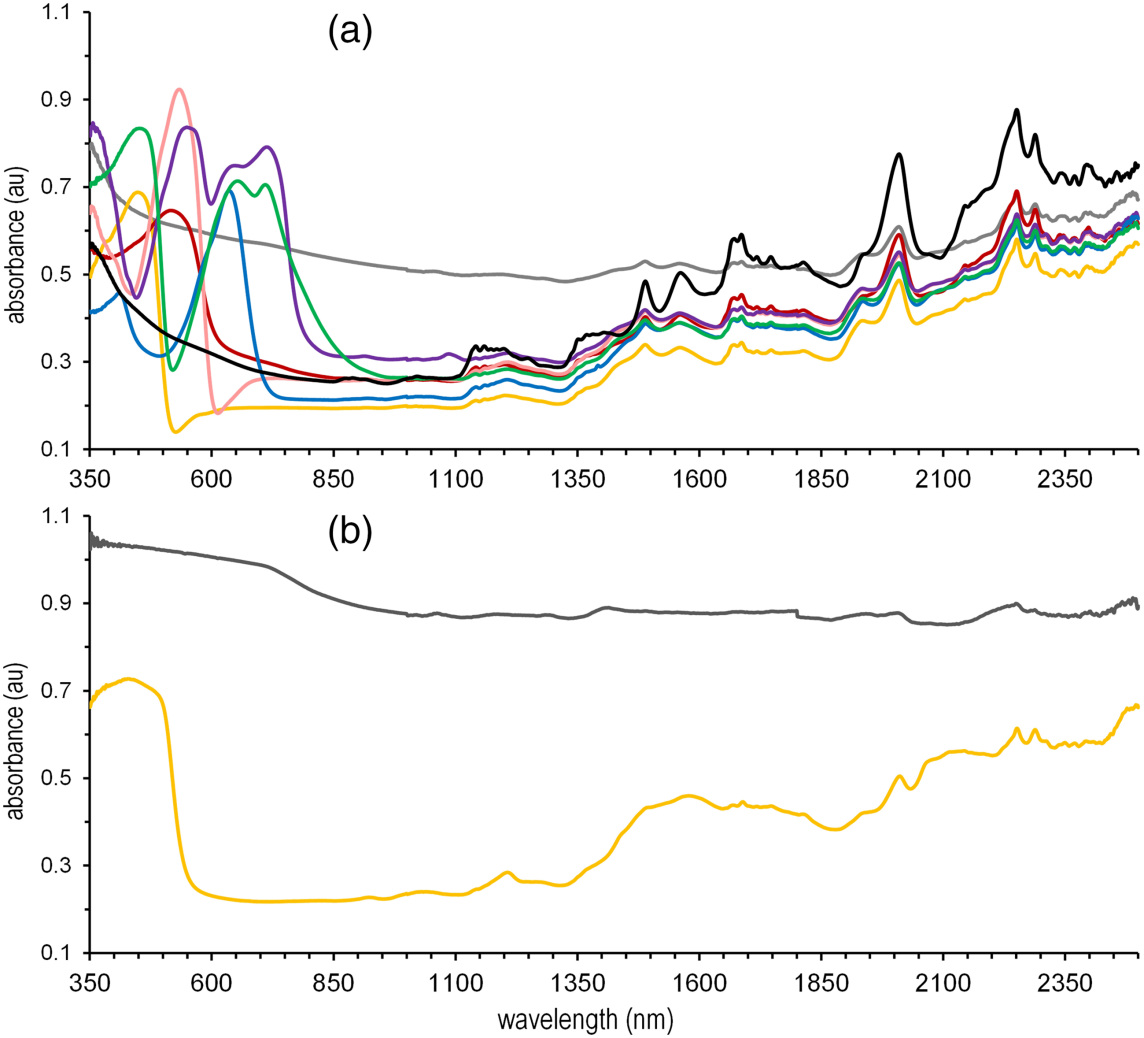
VIS‐NIR spectrum of crushed MDMA‐containing ecstasy tablets. Plots in (a) were correctly identified as MDMA‐containing. Plots in (b) are samples with a false negative result on the MDMA matrix libraries. (a) P1 (gray); P9 (yellow); P13 (pink); P16 (red); P18 (purple); P25 (blue); P37 (green), and pure MDMA HCl hydrate (black). (b) P19 (yellow) and P30 (dark gray); the plot colors correspond with the actual color of the ecstasy tablets. [Colour figure can be viewed at wileyonlinelibrary.com]

For both MDMA matrix libraries, a total of 14 out of the 362 scans from MDMA‐containing samples returned a false negative outcome. These erroneous results all originated from intact or crushed tablets (sets T, T2, and P). All MDMA‐containing casework in the other sets (crystals, powders) were correctly predicted irrespective of the color of the sample. The false negative results came from a black‐colored (rectangle tablet, imprint: Rolls Royce logo; T30) and a yellow‐colored (rectangle tablet, imprint “gold”; T19) ecstasy tablet and their respective crushed powders (P30, P19). For the crushed powder of the yellow tablet, MDMA was correctly detected in one out of three scans, whereas only an indication for MDMA (similarity score between 0.70 and 0.80) was obtained for the other two scans. In addition, three different ecstasy tablets in the 2021 casework set T2 gave false negative results. Interestingly, these originated from tablets with similar colors: a yellow rectangle tablet with imprint “gold” (different in size than T19), a black battery‐shaped tablet, imprint: “Duracell,” and a black tablet in the shape of the Philipp Plein logo. All other tablets in a wide diversity of colors were correctly identified; even other tablets with a yellow or dark gray/black color. A possible explanation for this phenomenon is that only certain particular colorants with a black and yellow color absorb light beyond the visible range into the 1500–2600 nm NIR range, thereby obscuring diagnostic spectral fingerprints of the active ingredient. Other colorants, including other types of yellow and black dyes, have a less detrimental effect still allowing MDMA detection by the method described in this study. To substantiate this, data from these crushed tablets that were earlier recorded using a 350–2500 nm visible‐NIR (VIS‐NIR) spectrometer^36^, ^41^ were examined with emphasis on their color. Figure 4 shows the VIS‐NIR spectra of seven differently colored MDMA‐containing powdered ecstasy tablets that were correctly identified by the software as examples: sample P1 (gray), P9 (yellow), P13 (pink), P16 (red), P18 (purple), P25 (blue), and P37 (green). These spectra are overlaid with the spectra of the two false negatives: P19 (yellow, dashed line) and P30 (dark gray, dashed line). The reference MDMA HCl·H_2_O spectrum is shown in black to emphasize the spectral peaks that could be attributed to MDMA. It is obvious that the intense absorption bands in the visible wavelength range (350–800 nm) originate from the different coloring agents in the tablets. For most tablets, this absorption is significantly reduced from ~800 nm onward into the NIR. However, for the gray (P1) and dark gray/black (P30) colored tablets, the high absorption continues throughout the entire NIR spectrum, also partly obscuring the features diagnostic for MDMA. For the yellow tablet T19/P19 in which the presence of MDMA was missed by the software, this effect was less detrimental although broad absorption bands were visible between 1400–1600 nm and 2000–2200 nm (Figure 4, dashed yellow line).

Remarkably, in all MDMA‐containing casework material (crystals, powders, tablets, crushed tablets), only the hydrated MDMA HCl variant was identified. The anhydrous variant was not detected in these sample sets. When the same data were processed on an MDMA matrix library in which the anhydrous form of MDMA was omitted, the same characteristics in terms of true positives (348 out of 362 scans) and the same false negatives (14 out of 362 scans) were reported. Contrary, the number of false positive results for MDMA further decreased from seven out of 1154 scans (0.6%) to two out of 1154 scans (0.2%) when excluding the anhydrous MDMA HCl polymorph from the library (Table 2). Misidentifications for MDMA were observed in one particular brown powder with unknown identity (H13, no peaks observed in GC‐MS analysis) that was identified as a complex mixture of cellulose (~60%), magnesium stearate (~15%), and MDMA either as HCl hydrate or in its anhydrous form at ~15%. In the MDMA matrix library containing both polymorphs, the anhydrate was reported (one out of three scans). In the MDMA matrix library containing only the hydrated form, this form was included in the result (two out of three scans). This sample was the only false positive result for MDMA in the matrix library containing only hydrated MDMA HCl. All other false positive results were related to the erroneous attribution of a small (<20%) amount of anhydrous MDMA HCl to explain the recorded NIR signal in the mixture detection part of the model. These were thus only observed for the MDMA matrix library including both polymorphous forms. A grinded 100 mg sildenafil citrate tablet was identified as 16% MDMA HCl anhydrate in ~55% microcrystalline cellulose in all replicates. Additionally, samples PAM86 and PAM183 (both mixtures of cocaine with levamisole) were explained as a mixture of MDMA HCl anhydrate with either mannitol or inositol in a total of three out of six scans. It must be noted that these false positives may easily be avoided when the spectral data were first processed on the cocaine matrix library that is more suitable for white powders. Earlier work showed that both samples were correctly identified as cocaine‐containing using this library.^17^

The decision to include either both MDMA HCl polymorphous forms or only the hydrated form in the matrix library could be made depending on the specific forensic setting in which the spectrometer is used. Although rare, anhydrous MDMA HCl does exist and may thus be encountered in a forensic setting. A false negative for this controlled substance is therefore unwanted. On the other hand, when prior knowledge indicates that changes to encounter this specific form are negligible or when other non‐spectroscopic tests are also performed (e.g., a colorimetric test with the Marquis reagent), only the frequently occurring hydrated form may be included to reduce false positives. Another approach is to determine specific decision rules in the multistage chemometric identification model. For example, when it is more likely to encounter anhydrous MDMA HCl in its pure form than processed in tablets, a possible option is to include both forms in the first LDA step and only the hydrated form in the subsequent NAS model for mixture detection. This way, pure anhydrous MDMA HCl will be detected, but no false positives will arise from this compound used to explain a minor part of the spectral signal in mixture deconvolution. Alternatively, a specific threshold for the minimum amount of anhydrous MDMA HCl present in a mixture could be set to avoid false positives from sub‐20% detections.

## 4 CONCLUSION AND FUTURE OUTLOOK

This study demonstrates the applicability of a 1300–2600 nm portable NIR spectrometer in combination with a multistage data analysis model to successfully detect heroin and MDMA in colored casework materials. Similarly to cocaine, casework samples of heroin and MDMA typically consist of a relatively confined set of related impurities, adulterants, or excipients. This makes these compounds suitable for detection by the NAS approach developed in earlier work that explains spectral signals by the optimal combination of spectra in a matrix library.^17^ A satisfactory false positive rate below 1% and a false negative rate less than 5% were observed for both heroin and MDMA in a wide variety of forensic samples. These samples included casework materials of various color (brown for heroin, different colors for ecstasy tablets), indicating that potential detrimental effects of the colorants did not significantly impact correct assignment of the active ingredient using this method. False negative results for MDMA were attributed to several black and yellow‐colored tablets in which the specific dyes in these samples were hypothesized to also absorb in the 1300–2600 nm NIR range and partly obscure the spectral signals from MDMA. For most other tablets, the colorant almost exclusively showed absorbance in the visible wavelength range.

For heroin, the 93% true positive rate illustrates the suitability of this approach for casework analysis. However, the low similarity scores (0.70–0.80) yielding false negatives and predicted compositions that do not account for the full sample mixture indicate that the model could not always sufficiently explain all spectral variation present. A probable explanation is incompleteness of the heroin matrix library. Future improvements may aim for extensive composition analysis of heroin casework samples (including salt and crystalline forms) by various analytical methods to reveal possible candidate substances to be added to the library. Additionally, inclusion of several heroin impurities such as 6‐MAM also is expected to increase model performance.

In NIR‐based methods for substance identification, different salt forms or crystal polymorphism of a single substance may complicate analysis as these may produce distinct NIR spectra. This study showed that regular brown heroin casework samples in the Netherlands contained heroin base, whereas regular MDMA crystals and MDMA‐containing ecstasy tablets contained the hydrated from of MDMA HCl. Future work on the occurrence of the various polymorphous forms of MDMA and their detectability by NIRS is currently in progress in our laboratory. Care must be taken to acquire and analyze reference materials in the same form as present in casework materials to avoid false negative results.

Combined with the conclusions from earlier work on cocaine,^17^ the results in this study show that the calibration friendly data analysis approach based on dedicated matrix libraries and mixture detection works sufficiently for various types of drugs‐of‐abuse casework. However, user knowledge is required to select the optimal matrix library based on the physical properties and expert recognition of the material (in analogy with colorimetric spot tests). Although multiple consecutive matrix libraries can be used to characterize the sample, this holds a risk of erroneous results due to the selection of the wrong library. An interesting future development may be to automatically process multiple libraries and report the result from the matrix library hit with the highest similarity score. Another option may be to expand the matrix library to include all frequently occurring drugs of abuse. However, this requires additional validation on model performance. The risk of erroneous results due to incompleteness of the matrix libraries could also be reduced by automatic monitoring of drug market developments to include NIR spectra of novel excipients in the library. A possible scenario is that samples with an inconclusive result or an unusually low match score are sent to the laboratory for detailed GC‐MS analysis. If a new adulterant or excipient is detected this way, their reference NIR spectra can be added to the library to improve model performance in future samples.

## CONFLICT OF INTEREST

HJR is managing partner at TIPb, the company commercializing the Powder Puck sensor.

## AUTHOR CONTRIBUTIONS

RFK: Conceptualization, methodology, investigation, data curation, project administration, writing—original draft; HJR: conceptualization, formal analysis, software, modeling, writing—review and editing; ACvA: supervision, writing—review and editing.

## Supporting information


**Figure S1.** NIR spectra (raw) of heroin and the corresponding samples shown in Figure 1.
**Figure S2.** NIR spectra (raw) of MDMA and the corresponding samples shown in Figure 2.
**Table S1.** Performance of the chemometric model on the various sample sets, results in samples.

**Data S1.** Supporting Information

**Data S2.** Supporting Information

**Data S3.** Supporting Information

## Data Availability

The data that support the findings of this study are available from the corresponding author upon reasonable request.
