# Supplementary material for: On‐site forensic analysis of colored seized materials: Detection of brown heroin and MDMA‐tablets by a portable NIR spectrometer
Source: Drug Test Anal. 2022 Aug 31;14(10):1762–72. doi: 10.1002/dta.3356 (PMC9804980; doi:10.1002/dta.3356)
Supplement: Supplementary file 1 — Figure S1. NIR spectra (raw) of heroin and the corresponding samples shown in Figure 1. Figure S2. NIR spectra (raw) of MDMA and the corresponding samples shown in Figure 2. Table S1. Performance of the chemometric model on the various sample sets, results in samples. [file DTA-14-1762-s003.pdf]

Supplemental Information

for

On-site forensic analysis of colored seized materials: detection of brown heroin and MDMA-tablets by a portable NIR spectrometer

Ruben F. Kranenburg[1,2,*], Henk-Jan Ramaker[3], Arian C. van Asten[2,4]

[1] Dutch National Police, Unit Amsterdam, Forensic Laboratory, Kabelweg 25, Amsterdam 1014 BA, The Netherlands

[2] Van 't Hoff Institute for Molecular Sciences, University of Amsterdam, Postbus 94157, Amsterdam 1090 GD, The Netherlands

[3] TIPb, Koningin Wilhelminaplein 30, Amsterdam 1062 KR, The Netherlands

[4] Co van Ledden Hulsebosch Center (CLHC), Amsterdam Center for Forensic Science and Medicine, Postbus 94157, Amsterdam 1090 GD, The Netherlands

[*] Corresponding author. *E-mail address:* ruben.kranenburg@politie.nl (R.F. Kranenburg).

Contents.

Figure S1. NIR spectra (raw) of heroin and the corresponding samples shown in Fig. 1.
Figure S2. NIR spectra (raw) of MDMA and the corresponding samples shown in Fig. 2.
Table S1. Performance of the chemometric model on the various sample sets, results in samples.

Individual results for all scans and samples can be found in the following separate supplementary files:

Results per sample_heroin matrix.pdf
Results per sample_MDMA matrix both forms.pdf
Results per sample_MDMA matrix hydrate only.pdf

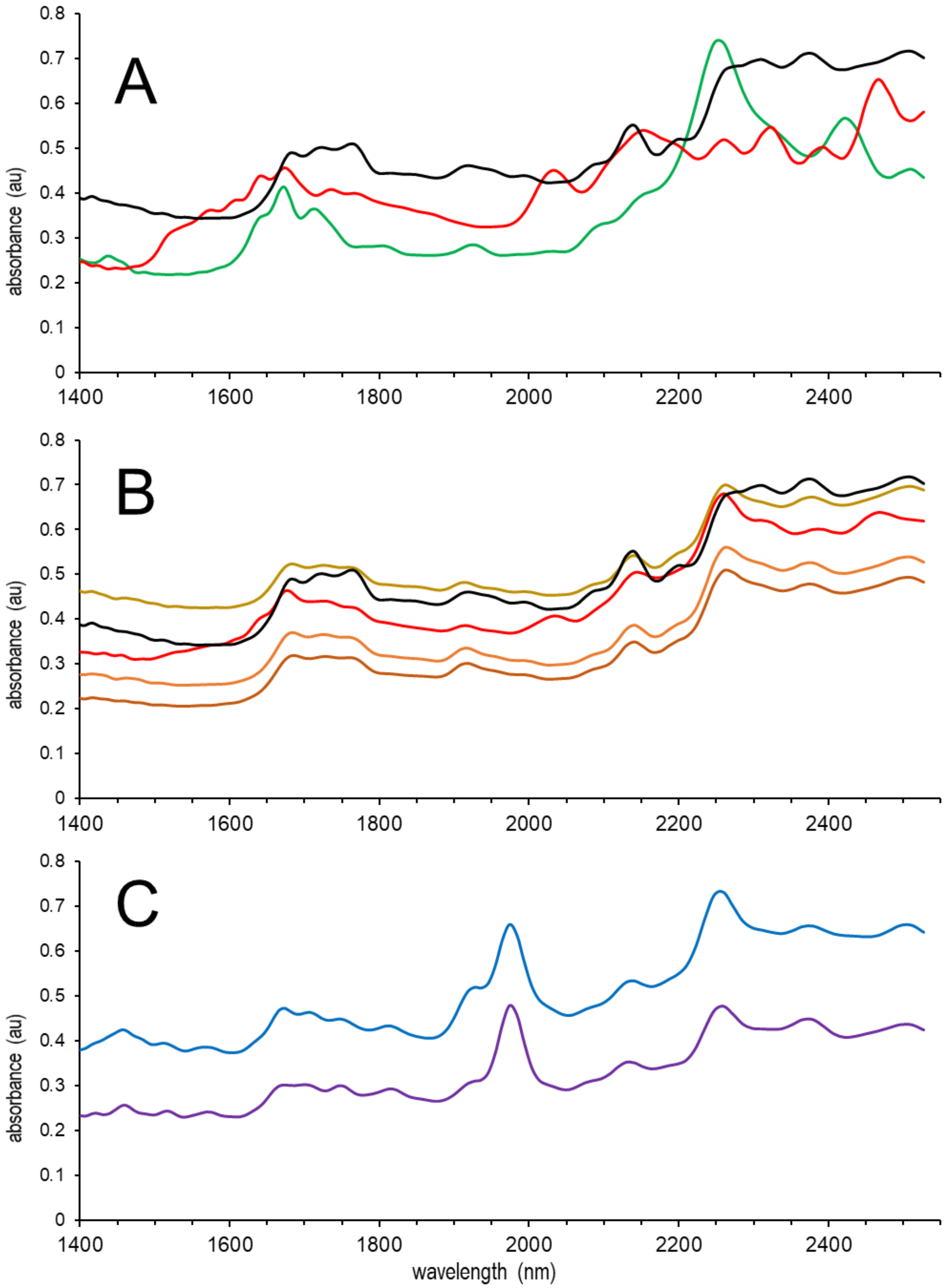

Figure S1. NIR spectra (raw) of heroin base (black trace in panels A and B) compared to common adulterants (panel A) and various brown heroin casework samples (panel B). Panel A: paracetamol (red), caffeine (green). Panel B: brown heroin samples (brown to orange shades), brown heroin sample highly adulterated with paracetamol and caffeine, H19 (red). Panel C depicts heroin HCl·H_2O (purple) and a white heroin casework sample, H8 (blue).

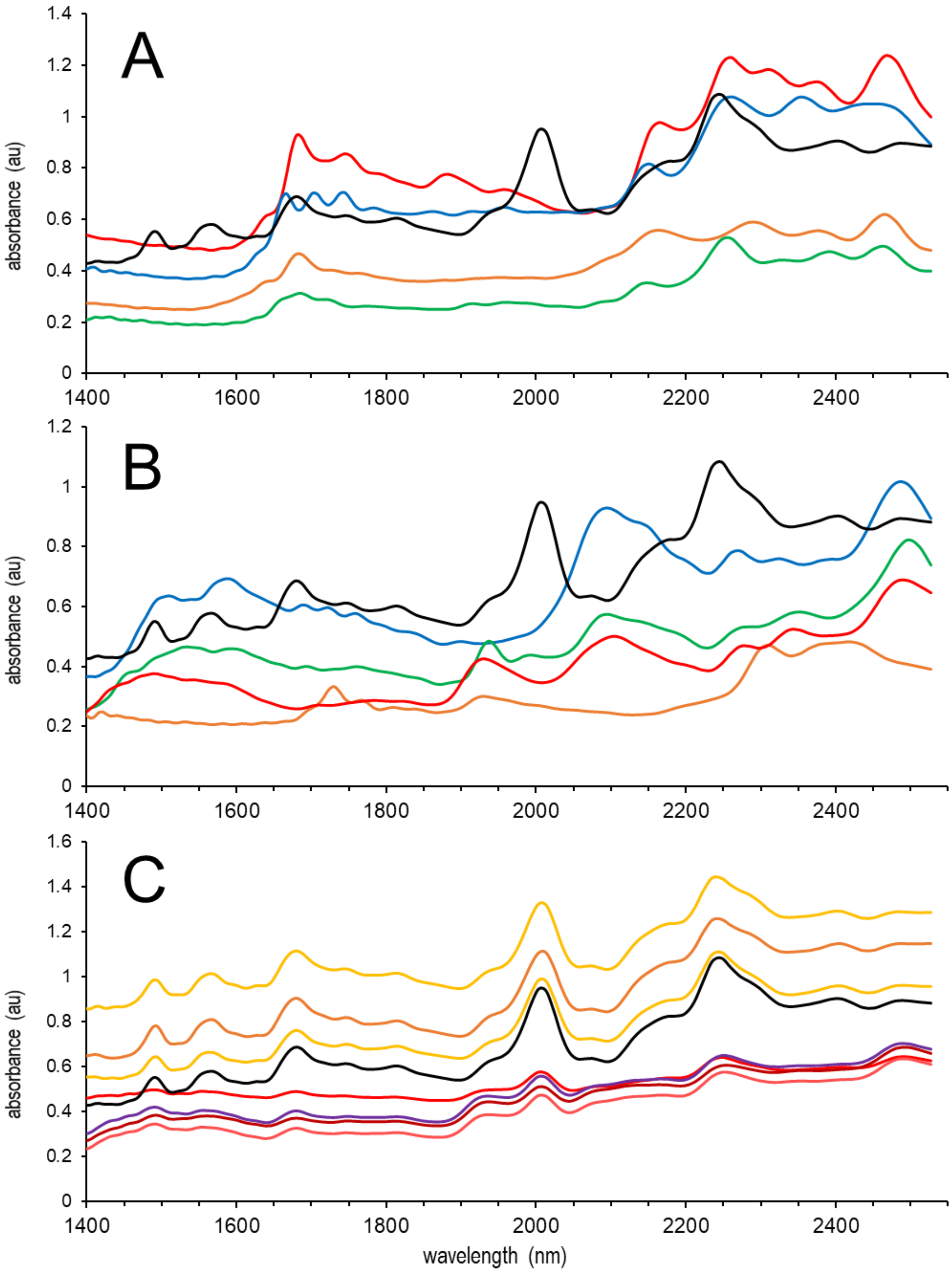

Figure S2. NIR spectra (raw) of MDMA HCl (black trace in all panels) compared to other drugs (panel A), common excipients (panel B) and various MDMA-containing casework samples (panel C). Panel A: methamphetamine HCl (red), cocaine HCl (green), ketamine (blue), amphetamine sulphate (orange). Panel B: microcrystalline cellulose (red), lactose (green), mannitol (blue), Mg stearate (orange). Panel C: MDMA crystals, M1-3 (orange shades), crushed MDMA-containing ecstasy tablets, P1-4 (purple to red shades).

Table S1. Performance of the chemometric model on the various sample sets, results in samples

			**heroin matrix library**			
sample set	pos#	TP	FN	neg#	TN	FP
H	46	42	4*	9	9	0
M	0	0	0	11	11	0
P	0	0	0	71	71	0
T	0	0	0	71	71	0
T2	0	0	0	65	65	0
C,D,N	3	3	0	92	92	0
PAM	0	0	0	181	181	0
TOTAL	*49*	*45*	*4*	*500*	*500*	*0*
			**MDMA matrix library (incl. anhydride)**			
sample set	pos#	TP	FN	neg#	TN	FP
H	0	0	0	55	55	0
M	11	11	0	0	0	0
P	39	37	2**	32	32	0
T	39	37	2	32	32	0
T2	47	44	3	18	18	0
C,D,N	3	3	0	92	91	1
PAM	13	13	0	168	167	1
TOTAL	*152*	*145*	*7*	*397*	*395*	*2*
			**MDMA matrix library (excl. anhydride)**			
sample set	pos#	TP	FN	neg#	TN	FP
H	0	0	0	55	54	1
M	11	11	0	0	0	0
P	39	37	2**	32	32	0
T	39	37	2	32	32	0
T2	47	44	3	18	18	0
C,D,N	3	3	0	92	92	0
PAM	13	13	0	168	168	0
TOTAL	*152*	*145*	*7*	*397*	*396*	*1*

Results per sample were calculated by majority voting of the individual scans. * For 3 samples in set H, 7 out of the 9 scans resulted in a match score between 0.70 - 0.80 (below threshold) for heroin. ** For one sample in set P, two out of the three scans resulted in a match score between 0.70 - 0.80 (below threshold) for MDMA. These samples were considered false negatives in this study.
