# Supplementary material for: On‐site forensic analysis of colored seized materials: Detection of brown heroin and MDMA‐tablets by a portable NIR spectrometer
Source: Drug Test Anal. 2022 Aug 31;14(10):1762–72. doi: 10.1002/dta.3356 (PMC9804980; doi:10.1002/dta.3356)
Supplement: Supplementary file 2 — Data S1. Supporting Information [file DTA-14-1762-s001.pdf]

RESULTS ON HEROIN MATRIX

legend:

- true positive & true negative
- false positive
- false negative
- false negative (similary 0.70 - 0.80)
- false positive (similary 0.70 - 0.80)

Sample	Identity	Powder Puck ID	Similarity
C1	Heroin (brown)	Heroin base (47%)	0.86
C1	Heroin (brown)	Heroin base (52%)	0.88
C1	Heroin (brown)	Heroin base (48%)	0.87
C17	Heroin (brown)	Heroin base (52%)	0.88
C17	Heroin (brown)	Heroin base (45%)	0.84
C17	Heroin (brown)	Heroin base (43%)	0.83
H1	Heroin	Heroin base (47%)	0.87
H1	Heroin	Heroin base (42%)	0.82
H1	Heroin	Heroin base (49%)	0.85
H2	Heroin	Heroin base (50%)	0.88
H2	Heroin	Heroin base (47%)	0.85
H2	Heroin	Heroin base (46%)	0.87
H3	Heroin	Heroin base (29%) + Paracetamol (37%)	0.88
H3	Heroin	Heroin base (35%) + Paracetamol (30%)	0.87
H3	Heroin	Heroin base (26%) + Paracetamol (34%) + Caffeine (17%)	0.92
H4	Heroin	Heroin base (40%)	0.82
H4	Heroin	Heroin base (41%)	0.83
H4	Heroin	Heroin base (41%)	0.82
H5	caffeine	Caffeine (32%) + Paracetamol (58%)	0.98
H5	caffeine	Caffeine (33%) + Paracetamol (57%)	0.98
H5	caffeine	Caffeine (31%) + Paracetamol (56%)	0.96
H6	negative	No result	0.34
H6	negative	No result	0.32
H6	negative	No result	0.34
H7	Heroin	Heroin base (31%)	0.80
H7	Heroin	Heroin base (41%)	0.83
H7	Heroin	Heroin base (39%)	0.82
H8	Heroin	Heroin HCl (64%) + Noscapine HCl (31%)	0.95
H8	Heroin	Heroin HCl (63%) + Noscapine HCl (31%)	0.94
H8	Heroin	Heroin HCl (63%) + Noscapine HCl (31%)	0.94
H9	Heroin	Heroin base (34%) + Caffeine (16%) + Paracetamol (15%)	0.85
H9	Heroin	Heroin base (33%)	0.75
H9	Heroin	Heroin base (37%)	0.79
H10	Heroin	Heroin base (47%)	0.87
H10	Heroin	Heroin base (49%)	0.89
H10	Heroin	Heroin base (48%)	0.87
H11	Heroin	Heroin base (43%) + Paracetamol (25%)	0.89
H11	Heroin	Heroin base (28%) + Paracetamol (26%) + Caffeine (16%)	0.90
H11	Heroin	Heroin base (44%) + Paracetamol (24%)	0.88
H12	Heroin	Heroin base (35%) + Paracetamol (33%)	0.88
H12	Heroin	Heroin base (38%) + Paracetamol (29%)	0.87
H12	Heroin	Heroin base (36%) + Paracetamol (32%)	0.87
H13	negative	No result	0.42
H13	negative	No result	0.43
H13	negative	No result	0.42
H14	Heroin	Heroin base (27%) + Paracetamol (26%) + Caffeine (18%)	0.90
H14	Heroin	Heroin base (29%) + Paracetamol (25%) + Caffeine (17%)	0.90
H14	Heroin	Heroin base (28%) + Paracetamol (26%) + Caffeine (18%)	0.90
H15	Heroin	Heroin base (21%) + Paracetamol (29%) + Noscapine HCl (19%)	0.87
H15	Heroin	Heroin base (35%) + Paracetamol (28%)	0.83
H15	Heroin	Heroin base (24%) + Paracetamol (28%) + Noscapine HCl (19%)	0.89
H16	paracetamol, caffeine	Caffeine (21%) + Paracetamol (70%)	0.98
H16	paracetamol, caffeine	Caffeine (22%) + Paracetamol (68%)	0.97
H16	paracetamol, caffeine	Caffeine (22%) + Paracetamol (68%)	0.97
H17	Heroin	Caffeine (28%) + Paracetamol (48%)	0.92
H17	Heroin	Caffeine (32%) + Paracetamol (49%)	0.95
H17	Heroin	Paracetamol (48%) + Caffeine (29%)	0.93

H18	Heroin	Heroin base (50%)	0.86
H18	Heroin	Heroin base (46%)	0.86
H18	Heroin	Heroin base (41%)	0.83
H19	Heroin	Heroin base (21%) + Paracetamol (25%) + Caffeine (16%) + Noscapine HCl (15%)	0.93
H19	Heroin	Heroin base (16%) + Paracetamol (30%) + Caffeine (17%) + Noscapine HCl (13%)	0.93
H19	Heroin	Heroin base (19%) + Paracetamol (37%) + Caffeine (24%)	0.94
H20	Heroin	Heroin base (13%) + Paracetamol (33%) + Caffeine (19%) + Noscapine HCl (12%)	0.92
H20	Heroin	Heroin base (12%) + Paracetamol (35%) + Caffeine (20%) + Noscapine HCl (13%)	0.95
H20	Heroin	Heroin base (30%) + Paracetamol (37%)	0.88
H21	Heroin	Heroin base (45%)	0.86
H21	Heroin	Heroin base (43%)	0.86
H21	Heroin	Heroin base (44%)	0.86
H22	Heroin	Heroin base (46%)	0.89
H22	Heroin	Heroin base (49%)	0.88
H22	Heroin	Heroin base (46%)	0.86
H23	Heroin	Heroin base (46%)	0.86
H23	Heroin	Heroin base (44%)	0.85
H23	Heroin	Heroin base (43%)	0.85
H24	Heroin	Heroin base (47%)	0.87
H24	Heroin	Heroin base (49%)	0.87
H24	Heroin	Heroin base (46%)	0.87
H25	Heroin	Heroin base (49%)	0.89
H25	Heroin	Heroin base (50%)	0.89
H25	Heroin	Heroin base (49%)	0.88
H26	PMK	No result	0.58
H26	PMK	No result	0.58
H26	PMK	No result	0.60
H27	Heroin	Heroin base (50%)	0.89
H27	Heroin	Heroin base (51%)	0.88
H27	Heroin	Heroin base (48%)	0.87
H28	Heroin	Heroin base (52%)	0.89
H28	Heroin	Heroin base (49%)	0.88
H28	Heroin	Heroin base (46%)	0.87
H29	Heroin	Heroin base (46%)	0.87
H29	Heroin	Heroin base (45%)	0.86
H29	Heroin	Heroin base (42%)	0.85
H30	Heroin	Heroin base (48%)	0.88
H30	Heroin	Heroin base (50%)	0.88
H30	Heroin	Heroin base (47%)	0.86
H31	Heroin	Heroin base (52%)	0.88
H31	Heroin	Heroin base (52%)	0.89
H31	Heroin	Heroin base (48%)	0.85
H32	Heroin	Heroin base (50%)	0.87
H32	Heroin	Heroin base (49%)	0.86
H32	Heroin	Heroin base (48%)	0.86
H33	Heroin	Heroin base (50%)	0.89
H33	Heroin	Heroin base (47%)	0.88
H33	Heroin	Heroin base (50%)	0.89
H34	tabacco	No result	0.33
H34	tabacco	No result	0.30
H34	tabacco	No result	0.36
H35	Heroin	Heroin base (49%)	0.87
H35	Heroin	Heroin base (43%) + Noscapine HCl (21%)	0.84
H35	Heroin	Heroin base (50%)	0.87
H36	Heroin	Heroin base (44%)	0.85
H36	Heroin	Heroin base (43%)	0.86
H36	Heroin	Heroin base (44%)	0.86
H37	instant cocoa	No result	0.29
H37	instant cocoa	No result	0.27
H37	instant cocoa	No result	0.30
H38	paracetamol, caffeine	Caffeine (29%) + Paracetamol (61%)	0.97
H38	paracetamol, caffeine	Caffeine (29%) + Paracetamol (60%)	0.97
H38	paracetamol, caffeine	Caffeine (29%) + Paracetamol (61%)	0.97
H39	Heroin	Heroin base (35%) + Caffeine (15%) + Paracetamol (18%)	0.87
H39	Heroin	Heroin base (34%)	0.80

RESULTS ON HEROIN MATRIX

H39	Heroin	Heroin base (36%) + Caffeine (16%) + Paracetamol (17%)	0.88
H40	Heroin	Heroin base (26%) + Paracetamol (36%) + Caffeine (19%)	0.94
H40	Heroin	Heroin base (21%) + Paracetamol (39%) + Caffeine (20%)	0.93
H40	Heroin	Heroin base (24%) + Paracetamol (37%) + Caffeine (19%)	0.93
H41	Heroin	Heroin base (31%)	0.76
H41	Heroin	Heroin base (30%)	0.76
H41	Heroin	Heroin base (43%) + Paracetamol (22%)	0.86
H42	Heroin	Heroin base (49%)	0.89
H42	Heroin	Heroin base (49%)	0.89
H42	Heroin	Heroin base (52%)	0.89
H43	Heroin	Heroin base (52%)	0.89
H43	Heroin	Heroin base (45%)	0.85
H43	Heroin	Heroin base (50%)	0.88
H44	Heroin	Heroin base (48%)	0.88
H44	Heroin	Heroin base (46%)	0.88
H44	Heroin	Heroin base (49%)	0.87
H45	paracetamol, caffeine	Caffeine (24%) + Paracetamol (53%) + Noscapine HCl (10%)	0.97
H45	paracetamol, caffeine	Caffeine (29%) + Paracetamol (62%)	0.98
H45	paracetamol, caffeine	Caffeine (27%) + Paracetamol (61%)	0.97
H46	Heroin	Heroin base (27%)	0.71
H46	Heroin	Heroin base (33%) + Caffeine (19%) + Paracetamol (16%)	0.86
H46	Heroin	Heroin base (36%) + Caffeine (20%) + Paracetamol (15%)	0.89
H47	Heroin	Heroin base (19%) + Paracetamol (32%) + Caffeine (16%) + Noscapine HCl (13%)	0.95
H47	Heroin	Heroin base (26%) + Paracetamol (38%) + Caffeine (20%)	0.96
H47	Heroin	Heroin base (23%) + Paracetamol (42%) + Caffeine (21%)	0.97
H48	Heroin	Heroin base (34%)	0.79
H48	Heroin	Heroin base (31%)	0.77
H48	Heroin	Heroin base (26%)	0.71
H49	Heroin	Heroin base (48%) + Noscapine HCl (22%)	0.89
H49	Heroin	Heroin base (51%) + Noscapine HCl (22%)	0.91
H49	Heroin	Heroin base (55%)	0.89
H50	Heroin	Heroin base (29%) + Paracetamol (21%) + Noscapine HCl (21%)	0.89
H50	Heroin	Heroin base (30%) + Paracetamol (21%) + Noscapine HCl (22%)	0.90
H50	Heroin	Heroin base (28%) + Paracetamol (23%) + Noscapine HCl (21%)	0.90
H51	Heroin base reference	Heroin base (65%)	0.96
H51	Heroin base reference	Heroin base (66%)	0.96
H51	Heroin base reference	Heroin base (69%)	0.97
H52	Heroin base reference	Heroin base (66%)	0.95
H52	Heroin base reference	Heroin base (66%)	0.95
H52	Heroin base reference	Heroin base (65%)	0.95
N23	Heroin (white)	Heroin HCl (83%)	0.98
N23	Heroin (white)	Heroin HCl (85%)	0.98
N23	Heroin (white)	Heroin HCl (85%)	0.98
M1	MDMA	No result	0.50
M1	MDMA	No result	0.46
M1	MDMA	No result	0.51
M2	MDMA	No result	0.50
M2	MDMA	No result	0.54
M2	MDMA	No result	0.51
M3	MDMA	No result	0.52
M3	MDMA	No result	0.53
M3	MDMA	No result	0.54
M4	MDMA	No result	0.53
M4	MDMA	No result	0.53
M4	MDMA	No result	0.54
M5	MDMA	No result	0.53
M5	MDMA	No result	0.52
M5	MDMA	No result	0.51
M6	MDMA	No result	0.52
M6	MDMA	No result	0.54
M6	MDMA	No result	0.51
M7	MDMA	No result	0.54
M7	MDMA	No result	0.51
M7	MDMA	No result	0.52
M8	MDMA	No result	0.50

M8	MDMA	No result	0.50
M8	MDMA	No result	0.48
M9	MDMA	No result	0.54
M9	MDMA	No result	0.54
M9	MDMA	No result	0.52
M10	MDMA	No result	0.50
M10	MDMA	No result	0.50
M10	MDMA	No result	0.49
M11	MDMA HCl reference	No result	0.52
M11	MDMA HCl reference	No result	0.52
M11	MDMA HCl reference	No result	0.51
P1	MDMA	No result	0.56
P1	MDMA	No result	0.56
P1	MDMA	No result	0.56
P2	MDMA	No result	0.46
P2	MDMA	No result	0.48
P2	MDMA	No result	0.50
P3	MDMA	No result	0.49
P3	MDMA	No result	0.54
P3	MDMA	No result	0.55
P4	MDMA	No result	0.51
P4	MDMA	No result	0.54
P4	MDMA	No result	0.52
P5	MDMA	No result	0.46
P5	MDMA	No result	0.47
P5	MDMA	No result	0.50
P6	MDMA	No result	0.56
P6	MDMA	No result	0.57
P6	MDMA	No result	0.56
P7	MDMA	No result	0.46
P7	MDMA	No result	0.44
P7	MDMA	No result	0.44
P8	MDMA	No result	0.56
P8	MDMA	No result	0.56
P8	MDMA	No result	0.58
P9	MDMA	No result	0.55
P9	MDMA	No result	0.55
P9	MDMA	No result	0.57
P10	MDMA	No result	0.55
P10	MDMA	No result	0.55
P10	MDMA	No result	0.55
P11	MDMA	No result	0.52
P11	MDMA	No result	0.55
P11	MDMA	No result	0.57
P12	MDMA	No result	0.57
P12	MDMA	No result	0.57
P12	MDMA	No result	0.57
P13	MDMA	No result	0.60
P13	MDMA	No result	0.57
P13	MDMA	No result	0.58
P14	MDMA	No result	0.58
P14	MDMA	No result	0.59
P14	MDMA	No result	0.57
P16	MDMA	No result	0.58
P16	MDMA	No result	0.57
P16	MDMA	No result	0.58
P17	MDMA	No result	0.55
P17	MDMA	No result	0.56
P17	MDMA	No result	0.56
P18	MDMA	No result	0.59
P18	MDMA	No result	0.59
P18	MDMA	No result	0.59
P19	MDMA	No result	0.42
P19	MDMA	No result	0.39
P19	MDMA	No result	0.41

P20	MDMA	No result	0.51
P20	MDMA	No result	0.53
P20	MDMA	No result	0.52
P21	MDMA	No result	0.56
P21	MDMA	No result	0.57
P21	MDMA	No result	0.56
P22	MDMA	No result	0.51
P22	MDMA	No result	0.54
P22	MDMA	No result	0.49
P23	MDMA	No result	0.59
P23	MDMA	No result	0.58
P23	MDMA	No result	0.58
P24	MDMA	No result	0.55
P24	MDMA	No result	0.56
P24	MDMA	No result	0.56
P25	MDMA	No result	0.51
P25	MDMA	No result	0.51
P25	MDMA	No result	0.53
P26	MDMA	No result	0.45
P26	MDMA	No result	0.49
P26	MDMA	No result	0.51
P27	MDMA	No result	0.48
P27	MDMA	No result	0.50
P27	MDMA	No result	0.49
P28	MDMA	No result	0.55
P28	MDMA	No result	0.56
P28	MDMA	No result	0.55
P29	MDMA	No result	0.32
P29	MDMA	No result	0.33
P29	MDMA	No result	0.32
P30	MDMA	No result	0.00
P30	MDMA	No result	0.00
P30	MDMA	No result	0.00
P31	MDMA	No result	0.57
P31	MDMA	No result	0.57
P31	MDMA	No result	0.56
P32	MDMA	No result	0.42
P32	MDMA	No result	0.44
P32	MDMA	No result	0.45
P33	MDMA	No result	0.53
P33	MDMA	No result	0.59
P33	MDMA	No result	0.59
P34	MDMA	No result	0.55
P34	MDMA	No result	0.58
P34	MDMA	No result	0.51
P35	MDMA	No result	0.57
P35	MDMA	No result	0.56
P35	MDMA	No result	0.58
P36	MDMA	No result	0.56
P36	MDMA	No result	0.56
P36	MDMA	No result	0.55
P37	MDMA	No result	0.57
P37	MDMA	No result	0.58
P37	MDMA	No result	0.56
P38	MDMA	No result	0.56
P38	MDMA	No result	0.57
P38	MDMA	No result	0.57
P39	MDMA	No result	0.44
P39	MDMA	No result	0.42
P39	MDMA	No result	0.43
P40	MDMA	No result	0.47
P40	MDMA	No result	0.46
P40	MDMA	No result	0.45
P101	2C-B	No result	0.29
P101	2C-B	No result	0.30

P101	2C-B	No result	0.29
P102	4-MMC	No result	0.36
P102	4-MMC	No result	0.36
P102	4-MMC	No result	0.39
P104	2C-B	No result	0.29
P104	2C-B	No result	0.30
P104	2C-B	No result	0.29
P105	2C-B	No result	0.36
P105	2C-B	No result	0.33
P105	2C-B	No result	0.33
P106	2Br45DMPEA	No result	0.30
P106	2Br45DMPEA	No result	0.30
P106	2Br45DMPEA	No result	0.32
P107	2Br45DMPEA	No result	0.30
P107	2Br45DMPEA	No result	0.31
P107	2Br45DMPEA	No result	0.32
P108	FA	No result	0.50
P108	FA	No result	0.47
P108	FA	No result	0.49
P109	FA	No result	0.49
P109	FA	No result	0.51
P109	FA	No result	0.49
P110	2C-B	No result	0.22
P110	2C-B	No result	0.26
P110	2C-B	No result	0.26
P111	2C-B	No result	0.23
P111	2C-B	No result	0.23
P111	2C-B	No result	0.22
P112	4-FMA	No result	0.46
P112	4-FMA	No result	0.44
P112	4-FMA	No result	0.42
P113	2Br45DMPEA	No result	0.30
P113	2Br45DMPEA	No result	0.31
P113	2Br45DMPEA	No result	0.31
P114	2C-B	No result	0.31
P114	2C-B	No result	0.29
P114	2C-B	No result	0.29
P115	pentylone	No result	0.42
P115	pentylone	No result	0.39
P115	pentylone	No result	0.36
P116	2C-B	No result	0.27
P116	2C-B	No result	0.27
P116	2C-B	No result	0.28
P117	FMA	No result	0.38
P117	FMA	No result	0.43
P117	FMA	No result	0.40
P118	2C-B	No result	0.28
P118	2C-B	No result	0.26
P118	2C-B	No result	0.26
P119	2C-B	No result	0.27
P119	2C-B	No result	0.25
P119	2C-B	No result	0.25
P120	2C-B	No result	0.29
P120	2C-B	No result	0.29
P120	2C-B	No result	0.29
P121	2C-B	No result	0.24
P121	2C-B	No result	0.25
P121	2C-B	No result	0.24
P122	2Br45DMPEA	No result	0.30
P122	2Br45DMPEA	No result	0.31
P122	2Br45DMPEA	No result	0.33
P123	FA	No result	0.51
P123	FA	No result	0.51
P123	FA	No result	0.50
P124	FA	No result	0.49

P124	FA	No result	0.50
P124	FA	No result	0.51
P125	FMA	No result	0.42
P125	FMA	No result	0.41
P125	FMA	No result	0.39
P126	4-FA	No result	0.48
P126	4-FA	No result	0.47
P126	4-FA	No result	0.47
P127	2C-B-fly	No result	0.35
P127	2C-B-fly	No result	0.34
P127	2C-B-fly	No result	0.33
P128	FMA	No result	0.40
P128	FMA	No result	0.38
P128	FMA	No result	0.37
P129	FA	No result	0.33
P129	FA	No result	0.32
P129	FA	No result	0.29
P130	FMA	No result	0.58
P130	FMA	No result	0.57
P130	FMA	No result	0.54
P131	mCPP	No result	0.35
P131	mCPP	No result	0.31
P131	mCPP	No result	0.34
P132	6-APB	No result	0.48
P132	6-APB	No result	0.50
P132	6-APB	No result	0.48
P133	4-FA	No result	0.48
P133	4-FA	No result	0.47
P133	4-FA	No result	0.47
T1	MDMA	No result	0.43
T1	MDMA	No result	0.45
T1	MDMA	No result	0.41
T2	MDMA	No result	0.45
T2	MDMA	No result	0.45
T2	MDMA	No result	0.40
T3	MDMA	No result	0.42
T3	MDMA	No result	0.42
T3	MDMA	No result	0.47
T4	MDMA	No result	0.46
T4	MDMA	No result	0.45
T4	MDMA	No result	0.48
T5	MDMA	No result	0.44
T5	MDMA	No result	0.42
T5	MDMA	No result	0.46
T6	MDMA	No result	0.53
T6	MDMA	No result	0.53
T6	MDMA	No result	0.54
T7	MDMA	No result	0.39
T7	MDMA	No result	0.38
T7	MDMA	No result	0.40
T8	MDMA	No result	0.53
T8	MDMA	No result	0.52
T8	MDMA	No result	0.55
T9	MDMA	No result	0.49
T9	MDMA	No result	0.48
T9	MDMA	No result	0.44
T10	MDMA	No result	0.52
T10	MDMA	No result	0.48
T10	MDMA	No result	0.48
T11	MDMA	No result	0.51
T11	MDMA	No result	0.54
T11	MDMA	No result	0.53
T12	MDMA	No result	0.53
T12	MDMA	No result	0.47
T12	MDMA	No result	0.52

T13	MDMA	No result	0.52
T13	MDMA	No result	0.53
T13	MDMA	No result	0.53
T14	MDMA	No result	0.50
T14	MDMA	No result	0.53
T14	MDMA	No result	0.50
T16	MDMA	No result	0.54
T16	MDMA	No result	0.54
T16	MDMA	No result	0.54
T17	MDMA	No result	0.51
T17	MDMA	No result	0.55
T17	MDMA	No result	0.49
T18	MDMA	No result	0.54
T18	MDMA	No result	0.53
T18	MDMA	No result	0.55
T19	MDMA	No result	0.32
T19	MDMA	No result	0.30
T19	MDMA	No result	0.35
T20	MDMA	No result	0.41
T20	MDMA	No result	0.43
T20	MDMA	No result	0.41
T21	MDMA	No result	0.57
T21	MDMA	No result	0.54
T21	MDMA	No result	0.55
T22	MDMA	No result	0.41
T22	MDMA	No result	0.40
T22	MDMA	No result	0.43
T23	MDMA	No result	0.54
T23	MDMA	No result	0.53
T23	MDMA	No result	0.55
T24	MDMA	No result	0.48
T24	MDMA	No result	0.45
T24	MDMA	No result	0.47
T25	MDMA	No result	0.47
T25	MDMA	No result	0.49
T25	MDMA	No result	0.47
T26	MDMA	No result	0.50
T26	MDMA	No result	0.44
T26	MDMA	No result	0.45
T27	MDMA	No result	0.45
T27	MDMA	No result	0.48
T27	MDMA	No result	0.42
T28	MDMA	No result	0.47
T28	MDMA	No result	0.51
T28	MDMA	No result	0.47
T29	MDMA	No result	0.31
T29	MDMA	No result	0.37
T29	MDMA	No result	0.34
T30	MDMA	No result	0.00
T30	MDMA	No result	0.00
T30	MDMA	No result	0.00
T31	MDMA	No result	0.45
T31	MDMA	No result	0.48
T31	MDMA	No result	0.45
T32	MDMA	No result	0.41
T32	MDMA	No result	0.38
T32	MDMA	No result	0.39
T33	MDMA	No result	0.48
T33	MDMA	No result	0.46
T33	MDMA	No result	0.48
T34	MDMA	No result	0.48
T34	MDMA	No result	0.51
T34	MDMA	No result	0.46
T35	MDMA	No result	0.55
T35	MDMA	No result	0.50

T35	MDMA	No result	0.54
T36	MDMA	No result	0.48
T36	MDMA	No result	0.48
T36	MDMA	No result	0.46
T37	MDMA	No result	0.49
T37	MDMA	No result	0.50
T37	MDMA	No result	0.51
T38	MDMA	No result	0.54
T38	MDMA	No result	0.53
T38	MDMA	No result	0.55
T39	MDMA	No result	0.37
T39	MDMA	No result	0.40
T39	MDMA	No result	0.40
T40	MDMA	No result	0.47
T40	MDMA	No result	0.40
T40	MDMA	No result	0.44
T101	2C-B	No result	0.31
T101	2C-B	No result	0.29
T101	2C-B	No result	0.30
T102	4-MMC	No result	0.33
T102	4-MMC	No result	0.34
T102	4-MMC	No result	0.34
T104	2C-B	No result	0.28
T104	2C-B	No result	0.30
T104	2C-B	No result	0.29
T105	2C-B	No result	0.32
T105	2C-B	No result	0.30
T105	2C-B	No result	0.31
T106	2C-B isomer	No result	0.31
T106	2C-B isomer	No result	0.29
T106	2C-B isomer	No result	0.30
T107	2C-B isomer	No result	0.31
T107	2C-B isomer	No result	0.30
T107	2C-B isomer	No result	0.27
T108	FA	No result	0.46
T108	FA	No result	0.49
T108	FA	No result	0.47
T109	FA	No result	0.49
T109	FA	No result	0.46
T109	FA	No result	0.48
T110	2C-B	No result	0.25
T110	2C-B	No result	0.28
T110	2C-B	No result	0.26
T111	2C-B	No result	0.27
T111	2C-B	No result	0.25
T111	2C-B	No result	0.24
T112	4-FMA	No result	0.39
T112	4-FMA	No result	0.42
T112	4-FMA	No result	0.39
T113	2C-B isomer	No result	0.33
T113	2C-B isomer	No result	0.34
T113	2C-B isomer	No result	0.31
T114	2C-B	No result	0.31
T114	2C-B	No result	0.33
T114	2C-B	No result	0.32
T115	pentylone	No result	0.41
T115	pentylone	No result	0.39
T115	pentylone	No result	0.38
T116	2C-B	No result	0.29
T116	2C-B	No result	0.30
T116	2C-B	No result	0.30
T117	FMA	No result	0.32
T117	FMA	No result	0.35
T117	FMA	No result	0.37
T118	2C-B	No result	0.27

T118	2C-B	No result	0.28
T118	2C-B	No result	0.28
T119	2C-B	No result	0.26
T119	2C-B	No result	0.25
T119	2C-B	No result	0.24
T120	2C-B	No result	0.31
T120	2C-B	No result	0.32
T120	2C-B	No result	0.31
T121	2C-B	No result	0.27
T121	2C-B	No result	0.24
T121	2C-B	No result	0.24
T122	2C-B isomer	No result	0.32
T122	2C-B isomer	No result	0.33
T122	2C-B isomer	No result	0.32
T123	FA	No result	0.49
T123	FA	No result	0.52
T123	FA	No result	0.51
T124	FA	No result	0.45
T124	FA	No result	0.48
T124	FA	No result	0.46
T125	FMA	No result	0.25
T125	FMA	No result	0.30
T125	FMA	No result	0.24
T126	4-FA	No result	0.42
T126	4-FA	No result	0.39
T126	4-FA	No result	0.39
T127	2C-B-fly	No result	0.34
T127	2C-B-fly	No result	0.34
T127	2C-B-fly	No result	0.33
T128	FMA	No result	0.27
T128	FMA	No result	0.27
T128	FMA	No result	0.29
T129	FA	No result	0.29
T129	FA	No result	0.26
T129	FA	No result	0.27
T130	FMA	No result	0.50
T130	FMA	No result	0.54
T130	FMA	No result	0.51
T131	mCPP	No result	0.33
T131	mCPP	No result	0.35
T131	mCPP	No result	0.34
T132	6-APB	No result	0.50
T132	6-APB	No result	0.51
T132	6-APB	No result	0.48
T133	4-FA	No result	0.48
T133	4-FA	No result	0.54
T133	4-FA	No result	0.50
T2_1	2C-B; dark green; clown	No result	0.24
T2_2	2C-B; green; clown	No result	0.21
T2_3	2C-B; green; Mario	No result	0.20
T2_4	2C-B; light green; Moncler logo	No result	0.23
T2_5	2C-B; pink; fox	No result	0.24
T2_6	2C-B; pink; NASA logo	No result	0.34
T2_7	2C-B; pink; Plusle	No result	0.28
T2_8	2C-B; purple; Maybach logo	No result	0.24
T2_9	2C-B; salmon; 2cb	No result	0.25
T2_10	2C-B; salmon; griffin	No result	0.24
T2_11	2C-B; salmon; Oreo logo	No result	0.27
T2_12	2C-B; yellow; Pickachu	No result	0.17
T2_13	2C-B; yellow; Plusle	No result	0.28
T2_14	2C-B; yellow; robot	No result	0.24
T2_15	4-FA; pink; Duplo	No result	0.49
T2_16	amphetamine; blue; Bitcoin logo	No result	0.32
T2_17	FMA; gray; Tomorrowland logo	No result	0.38
T2_18	FMA; yellow; Tesla logo	No result	0.42

T2_19	MDMA; black; Duracell	No result	0.00
T2_20	MDMA; black; Philipp Plein logo	No result	0.00
T2_21	MDMA; blue; FCBarcelona logo	No result	0.47
T2_22	MDMA; blue; Porsche logo	No result	0.49
T2_23	MDMA; blue; Punisher logo	No result	0.56
T2_24	MDMA; cream; Coca Cola logo	No result	0.43
T2_25	MDMA; cream; Flugel logo	No result	0.37
T2_26	MDMA; cream; Maserati logo	No result	0.55
T2_27	MDMA; cream; Rolex logo	No result	0.44
T2_28	MDMA; green; four leaf clover	No result	0.48
T2_29	MDMA; green; Heineken logo	No result	0.51
T2_30	MDMA; green; Jurrasic Park logo	No result	0.48
T2_31	MDMA; green; Nike sneaker	No result	0.45
T2_32	MDMA; grey; Jurrasic Park logo	No result	0.43
T2_33	MDMA; light blue; Philipp Plein logo	No result	0.51
T2_34	MDMA; light gray; granate	No result	0.42
T2_35	MDMA; light yellow; Casa de Papel logo	No result	0.38
T2_36	MDMA; light yellow; Philipp Plein logo	No result	0.44
T2_37	MDMA; light yellow; Punisher logo	No result	0.54
T2_38	MDMA; light yellow; Trump	No result	0.44
T2_39	MDMA; ocher; Mickey Mouse	No result	0.53
T2_40	MDMA; ocher; Mybrand logo	No result	0.45
T2_41	MDMA; orange; AUDI logo	No result	0.40
T2_42	MDMA; orange; Fanta logo	No result	0.39
T2_43	MDMA; orange; Michelin logo	No result	0.48
T2_44	MDMA; orange; Soundcloud logo	No result	0.44
T2_45	MDMA; pink; Casa de Papel logo	No result	0.38
T2_46	MDMA; pink; Skittles shape	No result	0.49
T2_47	MDMA; pink; Strawberry	No result	0.53
T2_48	MDMA; pink; Superman logo	No result	0.42
T2_49	MDMA; purple; Bugatti logo	No result	0.52
T2_50	MDMA; purple; shield	No result	0.43
T2_51	MDMA; purple; trump	No result	0.51
T2_52	MDMA; red; DJ	No result	0.54
T2_53	MDMA; red; Nintendo	No result	0.56
T2_54	MDMA; red; Punisher logo	No result	0.44
T2_55	MDMA; red; Skittles shape	No result	0.56
T2_56	MDMA; red; WIFI logo	No result	0.50
T2_57	MDMA; salmon; Punisher logo	No result	0.50
T2_58	MDMA; white; smiley	No result	0.50
T2_59	MDMA; yellow; Ducati logo	No result	0.54
T2_60	MDMA; yellow; Ghostbusters logo	No result	0.53
T2_61	MDMA; yellow; gold bar shape	No result	0.37
T2_62	MDMA; yellow; Minion shape	No result	0.47
T2_63	MDMA; yellow; Skittles shape	No result	0.50
T2_64	MDMA; yellow; Stewie shape	No result	0.50
T2_65	MDMA; yellow; Versace logo	No result	0.35
C1	Heroin (brown)	Heroin base (52%)	0.88
C1	Heroin (brown)	Heroin base (48%)	0.87
C1	Heroin (brown)	Heroin base (53%)	0.88
C2	GHB (powder)	No result	0.16
C2	GHB (powder)	No result	0.16
C2	GHB (powder)	No result	0.15
C3	cocaine base	No result	0.51
C3	cocaine base	No result	0.51
C3	cocaine base	No result	0.51
C4	cocaine HCl	No result	0.68
C4	cocaine HCl	No result	0.69
C4	cocaine HCl	No result	0.70
C5	MDMA	No result	0.52
C5	MDMA	No result	0.53
C5	MDMA	No result	0.52
C6	cocaine base	No result	0.51
C6	cocaine base	No result	0.51
C6	cocaine base	No result	0.51

C7	cocaine HCl	No result	0.69
C7	cocaine HCl	No result	0.70
C7	cocaine HCl	No result	0.69
C8	amphetamine	No result	0.44
C8	amphetamine	No result	0.41
C8	amphetamine	No result	0.44
C9	amphetamine	No result	0.43
C9	amphetamine	No result	0.42
C9	amphetamine	No result	0.41
C10	GHB (powder)	No result	0.14
C10	GHB (powder)	No result	0.14
C10	GHB (powder)	No result	0.15
C11	GHB (powder)	No result	0.00
C11	GHB (powder)	No result	0.18
C11	GHB (powder)	No result	0.19
C12	GHB (liquid)	No result	0.00
C12	GHB (liquid)	No result	0.00
C12	GHB (liquid)	No result	0.00
C13	ketamine	No result	0.47
C13	ketamine	No result	0.49
C13	ketamine	No result	0.49
C14	methamphetamine	No result	0.58
C14	methamphetamine	No result	0.55
C14	methamphetamine	No result	0.58
C15	methamphetamine	No result	0.55
C15	methamphetamine	No result	0.58
C15	methamphetamine	No result	0.57
C16	MDMA	No result	0.51
C16	MDMA	No result	0.54
C16	MDMA	No result	0.52
C17	Heroin (brown)	Heroin base (48%)	0.86
C17	Heroin (brown)	Heroin base (49%)	0.86
C17	Heroin (brown)	Heroin base (50%)	0.87
D1	2-CB	No result	0.53
D1	2-CB	No result	0.44
D1	2-CB	No result	0.51
D2	Caffeine	Caffeine (97%)	0.97
D2	Caffeine	Caffeine (82%)	0.95
D2	Caffeine	Caffeine (75%)	0.96
D3	non-dairy creamer	No result	0.37
D3	non-dairy creamer	No result	0.40
D3	non-dairy creamer	No result	0.37
D4	Ethylcathinon	No result	0.55
D4	Ethylcathinon	No result	0.54
D4	Ethylcathinon	No result	0.54
D5	N-ethylnorpentedron	No result	0.55
D5	N-ethylnorpentedron	No result	0.54
D5	N-ethylnorpentedron	No result	0.54
D6	MEC-CMC-CEC	No result	0.61
D6	MEC-CMC-CEC	No result	0.58
D6	MEC-CMC-CEC	No result	0.57
D7	FPM	No result	0.44
D7	FPM	No result	0.44
D7	FPM	No result	0.45
D8	levamisol	No result	0.49
D8	levamisol	No result	0.48
D8	levamisol	No result	0.48
D9	non-dairy creamer	No result	0.38
D9	non-dairy creamer	No result	0.38
D9	non-dairy creamer	No result	0.37
D10	cocaine	No result	0.67
D10	cocaine	No result	0.66
D10	cocaine	No result	0.66
D11	2-CB	No result	0.47
D11	2-CB	No result	0.50

D11	2-CB	No result	0.48
D12	3-MEC	No result	0.51
D12	3-MEC	No result	0.52
D12	3-MEC	No result	0.52
D13	4-MEC	No result	0.61
D13	4-MEC	No result	0.60
D13	4-MEC	No result	0.57
D14	4-MEC	No result	0.57
D14	4-MEC	No result	0.60
D14	4-MEC	No result	0.57
D15	4-MEC	No result	0.63
D15	4-MEC	No result	0.61
D15	4-MEC	No result	0.60
D16	2-FMA	No result	0.49
D16	2-FMA	No result	0.48
D16	2-FMA	No result	0.49
D17	4-FA	No result	0.49
D17	4-FA	No result	0.49
D17	4-FA	No result	0.50
D18	4-MMC	No result	0.41
D18	4-MMC	No result	0.44
D18	4-MMC	No result	0.44
D19	3,4-dMMC	No result	0.42
D19	3,4-dMMC	No result	0.42
D19	3,4-dMMC	No result	0.43
D20	2-MMC	No result	0.57
D20	2-MMC	No result	0.58
D20	2-MMC	No result	0.58
D21	3-MMC	No result	0.44
D21	3-MMC	No result	0.45
D21	3-MMC	No result	0.44
D22	3-MMC	No result	0.48
D22	3-MMC	No result	0.46
D22	3-MMC	No result	0.46
D23	4-CMC	No result	0.52
D23	4-CMC	No result	0.51
D23	4-CMC	No result	0.50
D24	N-ethylpentylon	No result	0.42
D24	N-ethylpentylon	No result	0.42
D24	N-ethylpentylon	No result	0.40
D25	3-CMC	No result	0.63
D25	3-CMC	No result	0.64
D25	3-CMC	No result	0.65
D26	4-CMC	No result	0.49
D26	4-CMC	No result	0.48
D26	4-CMC	No result	0.34
D27	2-FMA	No result	0.46
D27	2-FMA	No result	0.46
D27	2-FMA	No result	0.48
D28	3,4-dMMC	No result	0.43
D28	3,4-dMMC	No result	0.42
D28	3,4-dMMC	No result	0.42
D29	5-APB	No result	0.48
D29	5-APB	No result	0.49
D29	5-APB	No result	0.50
D30	6-APB	No result	0.38
D30	6-APB	No result	0.40
D30	6-APB	No result	0.42
D31	DMMC	No result	0.41
D31	DMMC	No result	0.43
D31	DMMC	No result	0.41
D32	CMC	No result	0.48
D32	CMC	No result	0.32
D32	CMC	No result	0.33
D33	4-MC	No result	0.55

D33	4-MC	No result	0.57
D33	4-MC	No result	0.57
D34	Pentedrone	No result	0.40
D34	Pentedrone	No result	0.42
D34	Pentedrone	No result	0.40
D35	4-CMC	No result	0.53
D35	4-CMC	No result	0.52
D35	4-CMC	No result	0.38
D36	4-CMC	No result	0.37
D36	4-CMC	No result	0.36
D36	4-CMC	No result	0.37
D37	cocaine HCl	No result	0.68
D37	cocaine HCl	No result	0.68
D37	cocaine HCl	No result	0.67
D38	4-CEC	No result	0.45
D38	4-CEC	No result	0.45
D38	4-CEC	No result	0.46
N1	paracetamol	Paracetamol (98%)	0.98
N1	paracetamol	Paracetamol (99%)	0.99
N1	paracetamol	Paracetamol (99%)	0.99
N2	caffeine	Caffeine (98%)	0.98
N2	caffeine	Caffeine (99%)	0.99
N2	caffeine	Caffeine (98%)	0.98
N3	levamisole	No result	0.46
N3	levamisole	No result	0.45
N3	levamisole	No result	0.46
N4	lidocaine	No result	0.49
N4	lidocaine	No result	0.49
N4	lidocaine	No result	0.49
N5	phenacetin	No result	0.58
N5	phenacetin	No result	0.58
N5	phenacetin	No result	0.57
N6	procaine	No result	0.56
N6	procaine	No result	0.55
N6	procaine	No result	0.55
N7	benzocaine	No result	0.07
N7	benzocaine	No result	0.20
N7	benzocaine	No result	0.08
N8	mannitol	No result	0.33
N8	mannitol	No result	0.30
N8	mannitol	No result	0.30
N9	lactose	No result	0.21
N9	lactose	No result	0.23
N9	lactose	No result	0.23
N10	vitamin C	No result	0.00
N10	vitamin C	No result	0.11
N10	vitamin C	No result	0.11
N11	sugar (powdered)	No result	0.22
N11	sugar (powdered)	No result	0.21
N11	sugar (powdered)	No result	0.25
N12	glucose	No result	0.00
N12	glucose	No result	0.00
N12	glucose	No result	0.00
N13	boric acid	No result	0.13
N13	boric acid	No result	0.15
N13	boric acid	No result	0.11
N14	diltiazem	No result	0.53
N14	diltiazem	No result	0.49
N14	diltiazem	No result	0.50
N15	prometazine	No result	0.41
N15	prometazine	No result	0.41
N15	prometazine	No result	0.40
N16	non-dairy creamer	No result	0.37
N16	non-dairy creamer	No result	0.39
N16	non-dairy creamer	No result	0.37

N17	wheat flour	No result	0.20
N17	wheat flour	No result	0.23
N17	wheat flour	No result	0.19
N18	acetylsalicylic acid	No result	0.64
N18	acetylsalicylic acid	No result	0.54
N18	acetylsalicylic acid	No result	0.62
N19	ketamine	No result	0.47
N19	ketamine	No result	0.49
N19	ketamine	No result	0.48
N20	amphetamine	No result	0.46
N20	amphetamine	No result	0.46
N20	amphetamine	No result	0.44
N21	MDMA (powder)	No result	0.53
N21	MDMA (powder)	No result	0.54
N21	MDMA (powder)	No result	0.54
N22	methamphetamine	No result	0.56
N22	methamphetamine	No result	0.57
N22	methamphetamine	No result	0.60
N23	Heroin (white)	Heroin HCl (98%)	0.98
N23	Heroin (white)	Heroin HCl (84%)	0.98
N23	Heroin (white)	Heroin HCl (99%)	0.99
N24	sildenafil citrate tablet, grinded	No result	0.38
N24	sildenafil citrate tablet, grinded	No result	0.36
N24	sildenafil citrate tablet, grinded	No result	0.39
N25	oxazepam tablet, grinded	No result	0.33
N25	oxazepam tablet, grinded	No result	0.34
N25	oxazepam tablet, grinded	No result	0.35
N26	flunitrazepam tablet, grinded	No result	0.26
N26	flunitrazepam tablet, grinded	No result	0.28
N26	flunitrazepam tablet, grinded	No result	0.26
N27	mephedrone	No result	0.41
N27	mephedrone	No result	0.42
N27	mephedrone	No result	0.44
N28	4-FA tablet, grinded	No result	0.33
N28	4-FA tablet, grinded	No result	0.34
N28	4-FA tablet, grinded	No result	0.33
N29	paracetamol :caffein, 1:1	Caffeine (35%) + Paracetamol (53%)	0.96
N29	paracetamol :caffein, 1:1	Caffeine (33%) + Paracetamol (53%)	0.96
N29	paracetamol :caffein, 1:1	Caffeine (35%) + Paracetamol (54%)	0.97
N30	levamisole:lidocaine, 1:1	No result	0.55
N30	levamisole:lidocaine, 1:1	No result	0.55
N30	levamisole:lidocaine, 1:1	No result	0.54
N31	levamisole:paracetamol:lidocaine, 1:1:1	Paracetamol (34%) + Noscapine HCl (8%) + Papaverine HCl (18%)	0.75
N31	levamisole:paracetamol:lidocaine, 1:1:1	Paracetamol (40%) + Papaverine HCl (19%)	0.76
N31	levamisole:paracetamol:lidocaine, 1:1:1	Paracetamol (38%) + Papaverine HCl (22%)	0.77
N32	levamisole:phenacetin, 1:1	No result	0.64
N32	levamisole:phenacetin, 1:1	No result	0.63
N32	levamisole:phenacetin, 1:1	No result	0.65
N33	phenacetin:lidocaine, 1:1	No result	0.45
N33	phenacetin:lidocaine, 1:1	No result	0.46
N33	phenacetin:lidocaine, 1:1	No result	0.45
N34	phenacetin:procaine, 1:1	No result	0.55
N34	phenacetin:procaine, 1:1	No result	0.56
N34	phenacetin:procaine, 1:1	No result	0.55
N35	levamisole:phenacetin:procaine, 1:1:1	No result	0.60
N35	levamisole:phenacetin:procaine, 1:1:1	No result	0.60
N35	levamisole:phenacetin:procaine, 1:1:1	No result	0.63
N36	paracetamol:phenacetin, 1:1	Paracetamol (56%)	0.90
N36	paracetamol:phenacetin, 1:1	Paracetamol (60%)	0.92
N36	paracetamol:phenacetin, 1:1	Paracetamol (62%)	0.92
N37	diazepam tablet 10 mg, grinded	No result	0.23
N37	diazepam tablet 10 mg, grinded	No result	0.27
N37	diazepam tablet 10 mg, grinded	No result	0.32
N38	methylphenidate 10 mg tablet, grinded	No result	0.00
N38	methylphenidate 10 mg tablet, grinded	No result	0.00

N38	methylphenidate 10 mg tablet, grinded	No result	0.00
N39	smartshop blend mix caffeine, lactose, mannitol	No result	0.47
N39	smartshop blend mix caffeine, lactose, mannitol	No result	0.46
N39	smartshop blend mix caffeine, lactose, mannitol	No result	0.45
N40	inositol	No result	0.00
N40	inositol	No result	0.00
N40	inositol	No result	0.00
PAM1	caffeine + levamisol	No result	0.41
PAM1	caffeine + levamisol	No result	0.42
PAM1	caffeine + levamisol	No result	0.35
PAM2	cocaine	No result	0.48
PAM2	cocaine	No result	0.47
PAM2	cocaine	No result	0.49
PAM3	cocaine	No result	0.65
PAM3	cocaine	No result	0.66
PAM3	cocaine	No result	0.66
PAM6	cocaine	No result	0.51
PAM6	cocaine	No result	0.52
PAM6	cocaine	No result	0.50
PAM7	cocaine + procaine	No result	0.65
PAM7	cocaine + procaine	No result	0.65
PAM7	cocaine + procaine	No result	0.65
PAM8	cocaine	No result	0.66
PAM8	cocaine	No result	0.66
PAM8	cocaine	No result	0.66
PAM9	cocaine + lidocaine + procaine + levamisole	No result	0.58
PAM9	cocaine + lidocaine + procaine + levamisole	No result	0.60
PAM9	cocaine + lidocaine + procaine + levamisole	No result	0.59
PAM11	MDMA	No result	0.52
PAM11	MDMA	No result	0.50
PAM11	MDMA	No result	0.51
PAM12	cocaine	No result	0.65
PAM12	cocaine	No result	0.67
PAM12	cocaine	No result	0.63
PAM13	cocaine	No result	0.64
PAM13	cocaine	No result	0.66
PAM13	cocaine	No result	0.63
PAM14	negatief	No result	0.12
PAM14	negatief	No result	0.11
PAM14	negatief	No result	0.12
PAM15	ketamine	No result	0.50
PAM15	ketamine	No result	0.56
PAM15	ketamine	No result	0.57
PAM16	cocaine + procaine + phenacetin	No result	0.55
PAM16	cocaine + procaine + phenacetin	No result	0.58
PAM16	cocaine + procaine + phenacetin	No result	0.57
PAM17	cocaine + lidocaine + phenacetin + levamisole	No result	0.60
PAM17	cocaine + lidocaine + phenacetin + levamisole	No result	0.60
PAM17	cocaine + lidocaine + phenacetin + levamisole	No result	0.58
PAM18	ketamine	No result	0.47
PAM18	ketamine	No result	0.48
PAM18	ketamine	No result	0.47
PAM19	ketamine	No result	0.46
PAM19	ketamine	No result	0.48
PAM19	ketamine	No result	0.46
PAM20	cocaine + phenacetin	No result	0.54
PAM20	cocaine + phenacetin	No result	0.52
PAM20	cocaine + phenacetin	No result	0.51
PAM21	cocaine	No result	0.67
PAM21	cocaine	No result	0.66
PAM21	cocaine	No result	0.66
PAM22	cocaine	No result	0.67
PAM22	cocaine	No result	0.66
PAM22	cocaine	No result	0.66
PAM23	cocaine	No result	0.53

PAM23	cocaine	No result	0.62
PAM23	cocaine	No result	0.62
PAM25	negatief	No result	0.00
PAM25	negatief	No result	0.00
PAM25	negatief	No result	0.00
PAM26	ketamine	No result	0.47
PAM26	ketamine	No result	0.41
PAM26	ketamine	No result	0.50
PAM27	cocaine + levamisole	No result	0.51
PAM27	cocaine + levamisole	No result	0.61
PAM27	cocaine + levamisole	No result	0.60
PAM28	cocaine	No result	0.68
PAM28	cocaine	No result	0.66
PAM28	cocaine	No result	0.66
PAM29	amphetamine	No result	0.45
PAM29	amphetamine	No result	0.45
PAM29	amphetamine	No result	0.50
PAM31	cocaine + levamisole	No result	0.67
PAM31	cocaine + levamisole	No result	0.67
PAM31	cocaine + levamisole	No result	0.67
PAM32	cocaine + lidocaine + caffeine + phenacetin + levamisole	No result	0.64
PAM32	cocaine + lidocaine + caffeine + phenacetin + levamisole	No result	0.59
PAM32	cocaine + lidocaine + caffeine + phenacetin + levamisole	No result	0.64
PAM33	cocaine + levamisole	No result	0.50
PAM33	cocaine + levamisole	No result	0.56
PAM33	cocaine + levamisole	No result	0.59
PAM35	cocaine + levamisole	No result	0.67
PAM35	cocaine + levamisole	No result	0.65
PAM35	cocaine + levamisole	No result	0.68
PAM36	cocaine + phenacetin + levamisole	No result	0.66
PAM36	cocaine + phenacetin + levamisole	No result	0.65
PAM36	cocaine + phenacetin + levamisole	No result	0.62
PAM37	paracetamol + caffeine	Paracetamol (74%)	0.93
PAM37	paracetamol + caffeine	Paracetamol (94%)	0.94
PAM37	paracetamol + caffeine	Paracetamol (74%)	0.92
PAM39	ketamine	No result	0.46
PAM39	ketamine	No result	0.47
PAM39	ketamine	No result	0.47
PAM40	lidocaine	No result	0.39
PAM40	lidocaine	No result	0.38
PAM40	lidocaine	No result	0.37
PAM41	cocaine + lidocaine + levamisole	No result	0.65
PAM41	cocaine + lidocaine + levamisole	No result	0.67
PAM41	cocaine + lidocaine + levamisole	No result	0.66
PAM42	cocaine	No result	0.66
PAM42	cocaine	No result	0.65
PAM42	cocaine	No result	0.67
PAM43	MDMA	No result	0.52
PAM43	MDMA	No result	0.52
PAM43	MDMA	No result	0.51
PAM44	cocaine	No result	0.64
PAM44	cocaine	No result	0.64
PAM44	cocaine	No result	0.66
PAM45	phenacetin	No result	0.56
PAM45	phenacetin	No result	0.56
PAM45	phenacetin	No result	0.56
PAM46	amphetamine	Caffeine (94%)	0.94
PAM46	amphetamine	Caffeine (68%)	0.92
PAM46	amphetamine	Caffeine (93%)	0.93
PAM47	cocaine	No result	0.65
PAM47	cocaine	No result	0.64
PAM47	cocaine	No result	0.65
PAM48	phenacetin	No result	0.29
PAM48	phenacetin	No result	0.28
PAM48	phenacetin	No result	0.28

PAM49	cocaine + phenacetin + levamisole	No result	0.61
PAM49	cocaine + phenacetin + levamisole	No result	0.64
PAM49	cocaine + phenacetin + levamisole	No result	0.62
PAM50	cocaine	No result	0.67
PAM50	cocaine	No result	0.62
PAM50	cocaine	No result	0.63
PAM51	cocaine + levamisole	No result	0.50
PAM51	cocaine + levamisole	No result	0.47
PAM51	cocaine + levamisole	No result	0.49
PAM52	cocaine + levamisole	No result	0.54
PAM52	cocaine + levamisole	No result	0.54
PAM52	cocaine + levamisole	No result	0.52
PAM53	negatief	No result	0.44
PAM53	negatief	No result	0.38
PAM53	negatief	No result	0.36
PAM56	MDMA	No result	0.53
PAM56	MDMA	No result	0.53
PAM56	MDMA	No result	0.52
PAM57	cocaine	No result	0.65
PAM57	cocaine	No result	0.63
PAM57	cocaine	No result	0.64
PAM58	cocaine	No result	0.52
PAM58	cocaine	No result	0.50
PAM58	cocaine	No result	0.56
PAM59	negatief	No result	0.00
PAM59	negatief	No result	0.00
PAM59	negatief	No result	0.00
PAM60	MDMA	No result	0.54
PAM60	MDMA	No result	0.52
PAM60	MDMA	No result	0.52
PAM61	amphetamine	No result	0.52
PAM61	amphetamine	No result	0.50
PAM62	cocaine + levamisole	No result	0.67
PAM62	cocaine + levamisole	No result	0.68
PAM62	cocaine + levamisole	No result	0.50
PAM63	cocaine + lidocaine + caffeine + phenacetin + levamisole	Heroin base (12%) + Paracetamol (23%) + Noscapine HCl (20%)	0.76
PAM63	cocaine + lidocaine + caffeine + phenacetin + levamisole	Heroin base (12%) + Paracetamol (22%) + Noscapine HCl (20%)	0.76
PAM63	cocaine + lidocaine + caffeine + phenacetin + levamisole	Heroin base (8%) + Paracetamol (19%) + Noscapine HCl (23%)	0.72
PAM64	cocaine	No result	0.61
PAM64	cocaine	No result	0.68
PAM64	cocaine	No result	0.66
PAM65	levamisol	No result	0.48
PAM65	levamisol	No result	0.48
PAM65	levamisol	No result	0.49
PAM66	lidocaine	No result	0.49
PAM66	lidocaine	No result	0.50
PAM66	lidocaine	No result	0.49
PAM67	levamisol	No result	0.48
PAM67	levamisol	No result	0.47
PAM67	levamisol	No result	0.47
PAM68	cocaine + phenacetin + levamisole	No result	0.56
PAM68	cocaine + phenacetin + levamisole	No result	0.53
PAM68	cocaine + phenacetin + levamisole	No result	0.58
PAM69	cocaine	No result	0.52
PAM69	cocaine	No result	0.50
PAM69	cocaine	No result	0.51
PAM71	negatief	No result	0.00
PAM71	negatief	No result	0.18
PAM71	negatief	No result	0.14
PAM72	cocaine + caffeine + phenacetin	No result	0.50
PAM72	cocaine + caffeine + phenacetin	No result	0.54
PAM72	cocaine + caffeine + phenacetin	No result	0.50
PAM73	cocaine + caffeine	No result	0.50
PAM73	cocaine + caffeine	No result	0.51
PAM73	cocaine + caffeine	No result	0.51

PAM74	MDMA	No result	0.52
PAM74	MDMA	No result	0.50
PAM74	MDMA	No result	0.52
PAM75	cocaine + levamisole	No result	0.67
PAM75	cocaine + levamisole	No result	0.67
PAM75	cocaine + levamisole	No result	0.68
PAM76	caffeine	No result	0.64
PAM76	caffeine	Caffeine (38%) + Papaverine HCl (12%)	0.71
PAM76	caffeine	Caffeine (39%) + Papaverine HCl (11%)	0.71
PAM77	phenacetin, caffeine, levamisol	Caffeine (39%) + Papaverine HCl (13%)	0.71
PAM77	phenacetin, caffeine, levamisol	No result	0.65
PAM77	phenacetin, caffeine, levamisol	Caffeine (35%) + Paracetamol (11%)	0.71
PAM78	cocaine	No result	0.61
PAM78	cocaine	No result	0.61
PAM78	cocaine	No result	0.58
PAM79	mitrazapine	No result	0.28
PAM79	mitrazapine	No result	0.28
PAM79	mitrazapine	No result	0.28
PAM80	cocaine	No result	0.67
PAM80	cocaine	No result	0.68
PAM80	cocaine	No result	0.68
PAM81	cocaine + levamisole	No result	0.39
PAM81	cocaine + levamisole	No result	0.51
PAM81	cocaine + levamisole	No result	0.51
PAM82	tetracaine	No result	0.49
PAM82	tetracaine	No result	0.48
PAM82	tetracaine	No result	0.49
PAM83	cocaine	No result	0.66
PAM83	cocaine	No result	0.65
PAM83	cocaine	No result	0.63
PAM84	ketamine	No result	0.46
PAM84	ketamine	No result	0.46
PAM84	ketamine	No result	0.48
PAM85	phenacetin	No result	0.56
PAM85	phenacetin	No result	0.56
PAM85	phenacetin	No result	0.55
PAM86	cocaine + levamisole	No result	0.48
PAM86	cocaine + levamisole	No result	0.50
PAM86	cocaine + levamisole	No result	0.47
PAM87	cocaine + levamisole	No result	0.65
PAM87	cocaine + levamisole	No result	0.67
PAM87	cocaine + levamisole	No result	0.65
PAM88	cocaine + lidocaine + procaine + levamisole	No result	0.60
PAM88	cocaine + lidocaine + procaine + levamisole	No result	0.60
PAM88	cocaine + lidocaine + procaine + levamisole	No result	0.59
PAM89	cocaine	No result	0.53
PAM89	cocaine	No result	0.59
PAM89	cocaine	No result	0.59
PAM90	negatief	No result	0.00
PAM90	negatief	No result	0.00
PAM90	negatief	No result	0.00
PAM91	cocaine + levamisole	No result	0.68
PAM91	cocaine + levamisole	No result	0.67
PAM91	cocaine + levamisole	No result	0.65
PAM92	amphetamine	No result	0.68
PAM92	amphetamine	No result	0.62
PAM92	amphetamine	Caffeine (30%)	0.73
PAM93	ketamine	No result	0.48
PAM93	ketamine	No result	0.47
PAM93	ketamine	No result	0.49
PAM94	cocaine + phenacetin	Paracetamol (59%) + Noscapine HCl (22%)	0.94
PAM94	cocaine + phenacetin	Paracetamol (59%) + Noscapine HCl (23%)	0.95
PAM94	cocaine + phenacetin	Paracetamol (55%) + Noscapine HCl (23%)	0.93
PAM95	phenacetin	No result	0.55
PAM95	phenacetin	No result	0.56

PAM95	phenacetin	No result	0.56
PAM96	cocaine	No result	0.54
PAM96	cocaine	No result	0.52
PAM96	cocaine	No result	0.58
PAM97	cocaine + levamisole	No result	0.52
PAM97	cocaine + levamisole	No result	0.54
PAM97	cocaine + levamisole	No result	0.51
PAM98	levamisol	No result	0.49
PAM98	levamisol	No result	0.49
PAM98	levamisol	No result	0.47
PAM99	cocaine + levamisole	No result	0.70
PAM99	cocaine + levamisole	Paracetamol (18%) + Noscapine HCl (28%)	0.71
PAM99	cocaine + levamisole	Paracetamol (18%) + Noscapine HCl (28%)	0.71
PAM101	cocaine + lidocaine + phenacetin + levamisole	Noscapine HCl (29%) + Paracetamol (18%)	0.72
PAM101	cocaine + lidocaine + phenacetin + levamisole	Noscapine HCl (30%) + Paracetamol (19%)	0.75
PAM101	cocaine + lidocaine + phenacetin + levamisole	Noscapine HCl (29%) + Paracetamol (18%)	0.73
PAM102	cocaine + lidocaine	No result	0.69
PAM102	cocaine + lidocaine	No result	0.68
PAM102	cocaine + lidocaine	No result	0.68
PAM103	cocaine + lidocaine + tetracaine + levamisole	No result	0.70
PAM103	cocaine + lidocaine + tetracaine + levamisole	No result	0.68
PAM103	cocaine + lidocaine + tetracaine + levamisole	No result	0.70
PAM104	cocaine + lidocaine + levamisole	No result	0.69
PAM104	cocaine + lidocaine + levamisole	No result	0.69
PAM104	cocaine + lidocaine + levamisole	No result	0.69
PAM105	amphetamine	No result	0.56
PAM105	amphetamine	No result	0.56
PAM105	amphetamine	No result	0.57
PAM106	ketamine	No result	0.49
PAM106	ketamine	No result	0.47
PAM106	ketamine	No result	0.47
PAM107	cocaine + lidocaine	No result	0.66
PAM107	cocaine + lidocaine	No result	0.66
PAM107	cocaine + lidocaine	No result	0.68
PAM108	cocaine + phenacetin	No result	0.57
PAM108	cocaine + phenacetin	No result	0.57
PAM108	cocaine + phenacetin	No result	0.56
PAM109	cocaine	No result	0.61
PAM109	cocaine	No result	0.60
PAM109	cocaine	No result	0.53
PAM110	metamphetamine	No result	0.59
PAM110	metamphetamine	No result	0.60
PAM110	metamphetamine	No result	0.60
PAM111	ketamine	No result	0.47
PAM111	ketamine	No result	0.46
PAM111	ketamine	No result	0.47
PAM112	cocaine + lidocaine	No result	0.50
PAM112	cocaine + lidocaine	No result	0.49
PAM112	cocaine + lidocaine	No result	0.49
PAM113	cocaine	No result	0.66
PAM113	cocaine	No result	0.66
PAM113	cocaine	No result	0.65
PAM114	cocaine + phenacetin + levamisole	No result	0.70
PAM114	cocaine + phenacetin + levamisole	No result	0.69
PAM114	cocaine + phenacetin + levamisole	No result	0.70
PAM115	MDMA	No result	0.54
PAM115	MDMA	No result	0.52
PAM115	MDMA	No result	0.54
PAM116	MDMA	No result	0.50
PAM116	MDMA	No result	0.52
PAM116	MDMA	No result	0.51
PAM117	MDMA	No result	0.51
PAM117	MDMA	No result	0.53
PAM117	MDMA	No result	0.54
PAM118	cocaine	No result	0.65

PAM118	cocaine	No result	0.61
PAM118	cocaine	No result	0.66
PAM119	cocaine	No result	0.68
PAM119	cocaine	No result	0.66
PAM119	cocaine	No result	0.68
PAM120	amphetamine	No result	0.49
PAM120	amphetamine	No result	0.51
PAM120	amphetamine	No result	0.50
PAM121	cocaine	No result	0.66
PAM121	cocaine	No result	0.66
PAM121	cocaine	No result	0.66
PAM122	cocaine + levamisole	Paracetamol (19%) + Noscapine HCl (31%)	0.75
PAM122	cocaine + levamisole	Paracetamol (19%) + Noscapine HCl (30%)	0.74
PAM122	cocaine + levamisole	Paracetamol (18%) + Noscapine HCl (29%)	0.73
PAM123	cocaine + lidocaine + benzocaine + phenacetin	No result	0.58
PAM123	cocaine + lidocaine + benzocaine + phenacetin	No result	0.61
PAM123	cocaine + lidocaine + benzocaine + phenacetin	No result	0.62
PAM124	cocaine + levamisole	No result	0.58
PAM124	cocaine + levamisole	No result	0.56
PAM124	cocaine + levamisole	No result	0.58
PAM125	amphetamine	No result	0.55
PAM125	amphetamine	No result	0.55
PAM125	amphetamine	No result	0.53
PAM126	amphetamine	No result	0.50
PAM126	amphetamine	No result	0.49
PAM126	amphetamine	No result	0.52
PAM127	cocaine + phenacetin + levamisole	No result	0.69
PAM127	cocaine + phenacetin + levamisole	No result	0.67
PAM127	cocaine + phenacetin + levamisole	No result	0.69
PAM128	cocaine + levamisole	No result	0.69
PAM128	cocaine + levamisole	No result	0.68
PAM128	cocaine + levamisole	No result	0.66
PAM129	cocaine	No result	0.67
PAM129	cocaine	No result	0.67
PAM129	cocaine	No result	0.65
PAM131	cocaine + caffeine + levamisole	Paracetamol (18%) + Noscapine HCl (28%)	0.71
PAM131	cocaine + caffeine + levamisole	Paracetamol (18%) + Noscapine HCl (28%)	0.72
PAM131	cocaine + caffeine + levamisole	Paracetamol (18%) + Noscapine HCl (28%)	0.71
PAM132	mannitol	No result	0.33
PAM132	mannitol	No result	0.32
PAM132	mannitol	No result	0.32
PAM133	cocaine + lidocaine	No result	0.67
PAM133	cocaine + lidocaine	No result	0.67
PAM133	cocaine + lidocaine	No result	0.67
PAM134	cocaine	No result	0.65
PAM134	cocaine	No result	0.67
PAM134	cocaine	No result	0.63
PAM135	cocaine + phenacetin	No result	0.55
PAM135	cocaine + phenacetin	No result	0.56
PAM135	cocaine + phenacetin	No result	0.55
PAM136	amphetamine	No result	0.22
PAM136	amphetamine	No result	0.28
PAM136	amphetamine	No result	0.25
PAM137	cocaine	No result	0.66
PAM137	cocaine	No result	0.66
PAM137	cocaine	No result	0.68
PAM139	metamphetamine	No result	0.56
PAM139	metamphetamine	No result	0.60
PAM139	metamphetamine	No result	0.57
PAM140	ketamine	No result	0.47
PAM140	ketamine	No result	0.48
PAM140	ketamine	No result	0.47
PAM141	MDMA	No result	0.48
PAM141	MDMA	No result	0.49
PAM141	MDMA	No result	0.50

RESULTS ON HEROIN MATRIX

PAM142	negatief	No result	0.34
PAM142	negatief	No result	0.32
PAM142	negatief	No result	0.34
PAM143	THC	No result	0.10
PAM143	THC	No result	0.07
PAM143	THC	No result	0.00
PAM144	cocaine	No result	0.67
PAM144	cocaine	No result	0.67
PAM144	cocaine	No result	0.67
PAM145	amphetamine	No result	0.44
PAM145	amphetamine	No result	0.49
PAM145	amphetamine	No result	0.47
PAM146	cocaine + lidocaine + procaine + tetracaine + phenacetin + levamisole	No result	0.63
PAM146	cocaine + lidocaine + procaine + tetracaine + phenacetin + levamisole	No result	0.60
PAM146	cocaine + lidocaine + procaine + tetracaine + phenacetin + levamisole	No result	0.66
PAM147	cocaine + lidocaine	No result	0.66
PAM147	cocaine + lidocaine	No result	0.67
PAM147	cocaine + lidocaine	No result	0.67
PAM148	amphetamine	Caffeine (60%) + Noscapine HCl (16%)	0.90
PAM148	amphetamine	Caffeine (62%) + Noscapine HCl (15%)	0.90
PAM148	amphetamine	Caffeine (58%)	0.91
PAM149	cocaine + lidocaine	No result	0.64
PAM149	cocaine + lidocaine	No result	0.60
PAM149	cocaine + lidocaine	No result	0.56
PAM150	cocaine + caffeine + levamisole	Noscapine HCl (33%) + Paracetamol (15%)	0.72
PAM150	cocaine + caffeine + levamisole	Paracetamol (15%) + Noscapine HCl (35%)	0.72
PAM150	cocaine + caffeine + levamisole	Paracetamol (16%) + Noscapine HCl (33%)	0.72
PAM151	cocaine	No result	0.53
PAM151	cocaine	No result	0.54
PAM151	cocaine	No result	0.55
PAM152	cocaine	No result	0.49
PAM152	cocaine	No result	0.47
PAM152	cocaine	No result	0.47
PAM153	cocaine + tetracaine + phenacetin + levamisole	Paracetamol (18%) + Noscapine HCl (30%)	0.71
PAM153	cocaine + tetracaine + phenacetin + levamisole	Noscapine HCl (30%) + Paracetamol (17%)	0.72
PAM153	cocaine + tetracaine + phenacetin + levamisole	Paracetamol (18%) + Noscapine HCl (31%)	0.72
PAM154	ketamine	No result	0.45
PAM154	ketamine	No result	0.45
PAM154	ketamine	No result	0.47
PAM156	ketamine	No result	0.47
PAM156	ketamine	No result	0.49
PAM156	ketamine	No result	0.47
PAM157	cocaine + tetracaine + caffeine + levamisole	No result	0.56
PAM157	cocaine + tetracaine + caffeine + levamisole	No result	0.49
PAM157	cocaine + tetracaine + caffeine + levamisole	No result	0.51
PAM158	cocaine + lidocaine + procaine + levamisole	No result	0.54
PAM158	cocaine + lidocaine + procaine + levamisole	No result	0.51
PAM158	cocaine + lidocaine + procaine + levamisole	No result	0.53
PAM159	cocaine + lidocaine + phenacetin	No result	0.56
PAM159	cocaine + lidocaine + phenacetin	No result	0.59
PAM159	cocaine + lidocaine + phenacetin	No result	0.54
PAM160	cocaine + lidocaine + phenacetin	No result	0.61
PAM160	cocaine + lidocaine + phenacetin	No result	0.58
PAM160	cocaine + lidocaine + phenacetin	No result	0.57
PAM161	cocaine	No result	0.52
PAM161	cocaine	No result	0.51
PAM161	cocaine	No result	0.51
PAM162	cocaine + caffeine + levamisole	No result	0.52
PAM162	cocaine + caffeine + levamisole	No result	0.49
PAM162	cocaine + caffeine + levamisole	No result	0.51
PAM163	cocaine	No result	0.45
PAM163	cocaine	No result	0.50
PAM163	cocaine	No result	0.47
PAM165	cocaine + procaine + phenacetin + levamisole	No result	0.56
PAM165	cocaine + procaine + phenacetin + levamisole	No result	0.52

PAM165	cocaine + procaine + phenacetin + levamisole	No result	0.54
PAM166	ketamine	No result	0.47
PAM166	ketamine	No result	0.48
PAM166	ketamine	No result	0.45
PAM167	cocaine	No result	0.61
PAM167	cocaine	No result	0.61
PAM167	cocaine	No result	0.60
PAM168	cocaine	No result	0.61
PAM168	cocaine	No result	0.57
PAM168	cocaine	No result	0.57
PAM169	cocaine	No result	0.53
PAM169	cocaine	No result	0.55
PAM169	cocaine	No result	0.54
PAM170	levamisol	No result	0.52
PAM170	levamisol	No result	0.51
PAM170	levamisol	No result	0.50
PAM171	cocaine	No result	0.55
PAM171	cocaine	No result	0.60
PAM171	cocaine	No result	0.43
PAM172	cocaine + levamisole	No result	0.67
PAM172	cocaine + levamisole	No result	0.66
PAM172	cocaine + levamisole	No result	0.66
PAM174	THC	No result	0.00
PAM174	THC	No result	0.00
PAM174	THC	No result	0.00
PAM175	cocaine + levamisole	No result	0.65
PAM175	cocaine + levamisole	No result	0.64
PAM175	cocaine + levamisole	No result	0.65
PAM176	cocaine + phenacetin	No result	0.50
PAM176	cocaine + phenacetin	No result	0.47
PAM176	cocaine + phenacetin	No result	0.52
PAM177	cocaine + levamisole	No result	0.68
PAM177	cocaine + levamisole	No result	0.68
PAM177	cocaine + levamisole	No result	0.67
PAM179	cocaine + procaine	No result	0.64
PAM179	cocaine + procaine	No result	0.65
PAM179	cocaine + procaine	No result	0.65
PAM180	MDMA	No result	0.52
PAM180	MDMA	No result	0.51
PAM180	MDMA	No result	0.51
PAM181	cocaine + caffeine	No result	0.66
PAM181	cocaine + caffeine	No result	0.67
PAM181	cocaine + caffeine	No result	0.67
PAM182	amphetamine	Caffeine (59%)	0.91
PAM182	amphetamine	Caffeine (61%) + Noscapine HCl (15%)	0.91
PAM182	amphetamine	Caffeine (91%)	0.91
PAM183	cocaine + levamisole	No result	0.41
PAM183	cocaine + levamisole	No result	0.36
PAM183	cocaine + levamisole	No result	0.42
PAM184	3-MMC	No result	0.43
PAM184	3-MMC	No result	0.44
PAM184	3-MMC	No result	0.44
PAM185	MDMA	No result	0.53
PAM185	MDMA	No result	0.52
PAM185	MDMA	No result	0.53
PAM186	cocaine + levamisole	No result	0.61
PAM186	cocaine + levamisole	No result	0.62
PAM186	cocaine + levamisole	No result	0.62
PAM188	cocaine	No result	0.67
PAM188	cocaine	No result	0.66
PAM188	cocaine	No result	0.68
PAM189	MDMA	No result	0.53
PAM189	MDMA	No result	0.53
PAM189	MDMA	No result	0.52
PAM190	cocaine + levamisole	No result	0.59

PAM190	cocaine + levamisole	No result	0.58
PAM190	cocaine + levamisole	No result	0.50
PAM191	ketamine	No result	0.49
PAM191	ketamine	No result	0.46
PAM191	ketamine	No result	0.47
PAM192	amphetamine	No result	0.44
PAM192	amphetamine	No result	0.44
PAM192	amphetamine	No result	0.34
PAM193	ketamine	No result	0.48
PAM193	ketamine	No result	0.47
PAM193	ketamine	No result	0.46
PAM194	cocaine + levamisole	No result	0.52
PAM194	cocaine + levamisole	No result	0.60
PAM194	cocaine + levamisole	No result	0.64
PAM195	cocaine	No result	0.51
PAM195	cocaine	No result	0.54
PAM195	cocaine	No result	0.52
PAM196	lidocaine	No result	0.41
PAM196	lidocaine	No result	0.41
PAM196	lidocaine	No result	0.42
PAM197	amphetamine	No result	0.52
PAM197	amphetamine	No result	0.49
PAM197	amphetamine	No result	0.51
PAM199	cocaine + phenacetin + levamisole	Heroin base (9%) + Paracetamol (17%) + Noscapine HCl (17%) + Papaverine CMN (10%)	0.72
PAM199	cocaine + phenacetin + levamisole	No result	0.69
PAM199	cocaine + phenacetin + levamisole	No result	0.56
PAM200	MDMA	No result	0.53
PAM200	MDMA	No result	0.52
PAM200	MDMA	No result	0.53
