# Supplementary material for: On‐site forensic analysis of colored seized materials: Detection of brown heroin and MDMA‐tablets by a portable NIR spectrometer
Source: Drug Test Anal. 2022 Aug 31;14(10):1762–72. doi: 10.1002/dta.3356 (PMC9804980; doi:10.1002/dta.3356)
Supplement: Supplementary file 4 — Data S3. Supporting Information [file DTA-14-1762-s004.pdf]

RESULTS ON MDMA MATRIX
(MDMA HCl·H20 only)

legend:

true positive & true negative

false positive

false negative

false negative (similary 0.70 - 0.80)

false positive (similary 0.70 - 0.80)

Sample	Identity	Powder Puck ID	Similarity
C1	Heroin (brown)	No result	0.29
C1	Heroin (brown)	No result	0.31
C1	Heroin (brown)	No result	0.30
C17	Heroin (brown)	No result	0.30
C17	Heroin (brown)	No result	0.29
C17	Heroin (brown)	No result	0.29
H1	Heroin	No result	0.29
H1	Heroin	No result	0.25
H1	Heroin	No result	0.54
H2	Heroin	No result	0.29
H2	Heroin	No result	0.00
H2	Heroin	No result	0.28
H3	Heroin	No result	0.00
H3	Heroin	No result	0.00
H3	Heroin	No result	0.36
H4	Heroin	No result	0.50
H4	Heroin	No result	0.50
H4	Heroin	No result	0.49
H5	caffeine	No result	0.21
H5	caffeine	No result	0.20
H5	caffeine	No result	0.00
H6	negative	No result	0.64
H6	negative	No result	0.70
H6	negative	Cellulose (36%) + Magnesium Stearate (11%)	0.71
H7	Heroin	No result	0.48
H7	Heroin	No result	0.53
H7	Heroin	No result	0.31
H8	Heroin	No result	0.26
H8	Heroin	No result	0.26
H8	Heroin	No result	0.25
H9	Heroin	No result	0.25
H9	Heroin	No result	0.20
H9	Heroin	No result	0.23
H10	Heroin	No result	0.52
H10	Heroin	No result	0.52
H10	Heroin	No result	0.53
H11	Heroin	No result	0.39
H11	Heroin	No result	0.39
H11	Heroin	No result	0.22
H12	Heroin	No result	0.20
H12	Heroin	No result	0.39
H12	Heroin	No result	0.21
H13	negative	MDMA HCl Hydrate (5%) + Cellulose (63%) + Magnesium Stearate (14%)	0.93
H13	negative	Cellulose (75%) + Magnesium Stearate (17%)	0.92
H13	negative	MDMA HCl Hydrate (4%) + Cellulose (61%) + Magnesium Stearate (15%)	0.92
H14	Heroin	No result	0.39
H14	Heroin	No result	0.25
H14	Heroin	No result	0.24
H15	Heroin	No result	0.26
H15	Heroin	No result	0.00
H15	Heroin	No result	0.39

H16	paracetamol, caffeine	No result	0.13
H16	paracetamol, caffeine	No result	0.12
H16	paracetamol, caffeine	No result	0.11
H17	Heroin	No result	0.28
H17	Heroin	No result	0.14
H17	Heroin	No result	0.28
H18	Heroin	No result	0.53
H18	Heroin	No result	0.51
H18	Heroin	No result	0.30
H19	Heroin	No result	0.40
H19	Heroin	No result	0.35
H19	Heroin	No result	0.33
H20	Heroin	No result	0.30
H20	Heroin	No result	0.29
H20	Heroin	No result	0.15
H21	Heroin	No result	0.55
H21	Heroin	No result	0.55
H21	Heroin	No result	0.55
H22	Heroin	No result	0.53
H22	Heroin	No result	0.54
H22	Heroin	No result	0.53
H23	Heroin	No result	0.53
H23	Heroin	No result	0.51
H23	Heroin	No result	0.51
H24	Heroin	No result	0.27
H24	Heroin	No result	0.33
H24	Heroin	No result	0.29
H25	Heroin	No result	0.33
H25	Heroin	No result	0.52
H25	Heroin	No result	0.53
H26	PMK	No result	0.38
H26	PMK	No result	0.39
H26	PMK	No result	0.40
H27	Heroin	No result	0.53
H27	Heroin	No result	0.52
H27	Heroin	No result	0.53
H28	Heroin	No result	0.53
H28	Heroin	No result	0.52
H28	Heroin	No result	0.53
H29	Heroin	No result	0.51
H29	Heroin	No result	0.51
H29	Heroin	No result	0.50
H30	Heroin	No result	0.52
H30	Heroin	No result	0.50
H30	Heroin	No result	0.55
H31	Heroin	No result	0.28
H31	Heroin	No result	0.56
H31	Heroin	No result	0.56
H32	Heroin	No result	0.52
H32	Heroin	No result	0.55
H32	Heroin	No result	0.51
H33	Heroin	No result	0.53
H33	Heroin	No result	0.53
H33	Heroin	No result	0.54
H34	tabacco	No result	0.51
H34	tabacco	No result	0.41
H34	tabacco	No result	0.52
H35	Heroin	No result	0.51
H35	Heroin	No result	0.54

RESULTS ON MDMA MATRIX
(MDMA HCl·H20 only)

H35	Heroin	No result	0.54
H36	Heroin	No result	0.49
H36	Heroin	No result	0.51
H36	Heroin	No result	0.27
H37	instant cocoa	Cellulose (28%) + Lactose (16%) + Magnesium Stearate (19%)	0.81
H37	instant cocoa	Cellulose (27%) + Lactose (15%) + Magnesium Stearate (15%)	0.77
H37	instant cocoa	Cellulose (28%) + Lactose (21%) + Magnesium Stearate (17%)	0.83
H38	paracetamol, caffeine	No result	0.22
H38	paracetamol, caffeine	No result	0.13
H38	paracetamol, caffeine	No result	0.21
H39	Heroin	No result	0.44
H39	Heroin	No result	0.23
H39	Heroin	No result	0.44
H40	Heroin	No result	0.36
H40	Heroin	No result	0.35
H40	Heroin	No result	0.36
H41	Heroin	No result	0.25
H41	Heroin	No result	0.26
H41	Heroin	No result	0.25
H42	Heroin	No result	0.34
H42	Heroin	No result	0.31
H42	Heroin	No result	0.53
H43	Heroin	No result	0.55
H43	Heroin	No result	0.51
H43	Heroin	No result	0.54
H44	Heroin	No result	0.54
H44	Heroin	No result	0.32
H44	Heroin	No result	0.54
H45	paracetamol, caffeine	No result	0.23
H45	paracetamol, caffeine	No result	0.23
H45	paracetamol, caffeine	No result	0.22
H46	Heroin	No result	0.21
H46	Heroin	No result	0.41
H46	Heroin	No result	0.42
H47	Heroin	No result	0.35
H47	Heroin	No result	0.36
H47	Heroin	No result	0.34
H48	Heroin	No result	0.21
H48	Heroin	No result	0.22
H48	Heroin	No result	0.23
H49	Heroin	No result	0.29
H49	Heroin	No result	0.48
H49	Heroin	No result	0.49
H50	Heroin	No result	0.00
H50	Heroin	No result	0.24
H50	Heroin	No result	0.25
H51	Heroin base reference	No result	0.31
H51	Heroin base reference	No result	0.59
H51	Heroin base reference	No result	0.58
H52	Heroin base reference	No result	0.43
H52	Heroin base reference	No result	0.57
H52	Heroin base reference	No result	0.57
N23	Heroin (white)	No result	0.00
N23	Heroin (white)	No result	0.00
N23	Heroin (white)	No result	0.21
M1	MDMA	MDMA HCl Hydrate (98%)	0.98
M1	MDMA	MDMA HCl Hydrate (52%)	0.95
M1	MDMA	MDMA HCl Hydrate (76%)	0.98
M2	MDMA	MDMA HCl Hydrate (98%)	0.98

RESULTS ON MDMA MATRIX
(MDMA HCl·H20 only)

M2	MDMA	MDMA HCl Hydrate (98%)	0.98
M2	MDMA	MDMA HCl Hydrate (99%)	0.99
M3	MDMA	MDMA HCl Hydrate (99%)	0.99
M3	MDMA	MDMA HCl Hydrate (99%)	0.99
M3	MDMA	MDMA HCl Hydrate (99%)	0.99
M4	MDMA	MDMA HCl Hydrate (99%)	0.99
M4	MDMA	MDMA HCl Hydrate (99%)	0.99
M4	MDMA	MDMA HCl Hydrate (99%)	0.99
M5	MDMA	MDMA HCl Hydrate (99%)	0.99
M5	MDMA	MDMA HCl Hydrate (99%)	0.99
M5	MDMA	MDMA HCl Hydrate (99%)	0.99
M6	MDMA	MDMA HCl Hydrate (99%)	0.99
M6	MDMA	MDMA HCl Hydrate (99%)	0.99
M6	MDMA	MDMA HCl Hydrate (97%)	0.97
M7	MDMA	MDMA HCl Hydrate (99%)	0.99
M7	MDMA	MDMA HCl Hydrate (96%)	0.96
M7	MDMA	MDMA HCl Hydrate (99%)	0.99
M8	MDMA	MDMA HCl Hydrate (97%)	0.97
M8	MDMA	MDMA HCl Hydrate (98%)	0.98
M8	MDMA	MDMA HCl Hydrate (96%)	0.96
M9	MDMA	MDMA HCl Hydrate (97%)	0.97
M9	MDMA	MDMA HCl Hydrate (98%)	0.98
M9	MDMA	MDMA HCl Hydrate (99%)	0.99
M10	MDMA	MDMA HCl Hydrate (97%)	0.97
M10	MDMA	MDMA HCl Hydrate (97%)	0.97
M10	MDMA	MDMA HCl Hydrate (56%)	0.93
M11	MDMA HCl reference	MDMA HCl Hydrate (100%)	1.00
M11	MDMA HCl reference	MDMA HCl Hydrate (100%)	1.00
M11	MDMA HCl reference	MDMA HCl Hydrate (100%)	1.00
P1	MDMA	MDMA HCl Hydrate (48%) + Cellulose (33%)	0.95
P1	MDMA	MDMA HCl Hydrate (48%) + Cellulose (34%)	0.96
P1	MDMA	MDMA HCl Hydrate (48%) + Cellulose (30%)	0.93
P2	MDMA	MDMA HCl Hydrate (34%) + Cellulose (48%)	0.96
P2	MDMA	MDMA HCl Hydrate (34%) + Cellulose (47%)	0.94
P2	MDMA	MDMA HCl Hydrate (37%) + Cellulose (47%)	0.95
P3	MDMA	MDMA HCl Hydrate (42%) + Cellulose (53%)	0.95
P3	MDMA	MDMA HCl Hydrate (48%) + Cellulose (48%)	0.96
P3	MDMA	MDMA HCl Hydrate (51%) + Cellulose (45%)	0.96
P4	MDMA	MDMA HCl Hydrate (42%) + Cellulose (53%)	0.95
P4	MDMA	MDMA HCl Hydrate (47%) + Cellulose (49%)	0.95
P4	MDMA	MDMA HCl Hydrate (45%) + Cellulose (51%)	0.96
P5	MDMA	MDMA HCl Hydrate (38%) + Cellulose (58%)	0.96
P5	MDMA	MDMA HCl Hydrate (38%) + Cellulose (58%)	0.95
P5	MDMA	MDMA HCl Hydrate (42%) + Cellulose (54%)	0.96
P6	MDMA	MDMA HCl Hydrate (51%) + Cellulose (45%)	0.95
P6	MDMA	MDMA HCl Hydrate (59%) + Cellulose (37%)	0.96
P6	MDMA	MDMA HCl Hydrate (60%) + Cellulose (36%)	0.96
P7	MDMA	MDMA HCl Hydrate (34%) + Cellulose (60%)	0.94
P7	MDMA	MDMA HCl Hydrate (30%) + Cellulose (65%)	0.95
P7	MDMA	MDMA HCl Hydrate (31%) + Cellulose (64%)	0.95
P8	MDMA	MDMA HCl Hydrate (32%) + Cellulose (37%) + Magnesium Stearate (15%)	0.95
P8	MDMA	MDMA HCl Hydrate (39%) + Cellulose (29%) + Magnesium Stearate (15%)	0.95
P8	MDMA	MDMA HCl Hydrate (38%) + Cellulose (31%) + Magnesium Stearate (15%)	0.95
P9	MDMA	MDMA HCl Hydrate (50%) + Cellulose (46%)	0.95
P9	MDMA	MDMA HCl Hydrate (48%) + Cellulose (46%)	0.94
P9	MDMA	MDMA HCl Hydrate (43%) + Cellulose (37%)	0.94
P10	MDMA	MDMA HCl Hydrate (67%) + Cellulose (29%)	0.96
P10	MDMA	MDMA HCl Hydrate (66%) + Cellulose (31%)	0.97
P10	MDMA	MDMA HCl Hydrate (67%) + Cellulose (30%)	0.96

RESULTS ON MDMA MATRIX
(MDMA HCl·H20 only)

P11	MDMA	MDMA HCl Hydrate (39%) + Cellulose (56%)	0.95
P11	MDMA	MDMA HCl Hydrate (43%) + Cellulose (39%)	0.94
P11	MDMA	MDMA HCl Hydrate (48%) + Cellulose (38%)	0.96
P12	MDMA	MDMA HCl Hydrate (53%) + Cellulose (29%)	0.95
P12	MDMA	MDMA HCl Hydrate (52%) + Cellulose (26%)	0.93
P12	MDMA	MDMA HCl Hydrate (47%) + Cellulose (31%)	0.93
P13	MDMA	MDMA HCl Hydrate (51%) + Cellulose (33%)	0.95
P13	MDMA	MDMA HCl Hydrate (54%) + Cellulose (40%)	0.94
P13	MDMA	MDMA HCl Hydrate (60%) + Cellulose (36%)	0.96
P14	MDMA	MDMA HCl Hydrate (55%) + Cellulose (40%)	0.95
P14	MDMA	MDMA HCl Hydrate (55%) + Cellulose (40%)	0.94
P14	MDMA	MDMA HCl Hydrate (54%) + Cellulose (34%)	0.96
P16	MDMA	MDMA HCl Hydrate (75%) + Cellulose (23%)	0.98
P16	MDMA	MDMA HCl Hydrate (96%)	0.96
P16	MDMA	MDMA HCl Hydrate (97%)	0.97
P17	MDMA	MDMA HCl Hydrate (58%) + Cellulose (39%)	0.97
P17	MDMA	MDMA HCl Hydrate (64%) + Cellulose (32%)	0.96
P17	MDMA	MDMA HCl Hydrate (63%) + Cellulose (34%)	0.97
P18	MDMA	MDMA HCl Hydrate (41%) + Cellulose (28%) + Magnesium Stearate (14%)	0.96
P18	MDMA	MDMA HCl Hydrate (42%) + Cellulose (28%) + Magnesium Stearate (14%)	0.96
P18	MDMA	MDMA HCl Hydrate (41%) + Cellulose (29%) + Magnesium Stearate (14%)	0.96
P19	MDMA	MDMA HCl Hydrate (24%) + Mannitol (16%) + Cellulose (19%)	0.80
P19	MDMA	MDMA HCl Hydrate (18%) + Mannitol (17%) + Cellulose (20%)	0.77
P19	MDMA	MDMA HCl Hydrate (25%) + Mannitol (14%) + Cellulose (18%)	0.79
P20	MDMA	MDMA HCl Hydrate (38%) + Cellulose (30%)	0.87
P20	MDMA	MDMA HCl Hydrate (39%) + Cellulose (30%)	0.90
P20	MDMA	MDMA HCl Hydrate (39%) + Cellulose (28%)	0.87
P21	MDMA	MDMA HCl Hydrate (63%) + Cellulose (33%)	0.97
P21	MDMA	MDMA HCl Hydrate (61%) + Cellulose (36%)	0.97
P21	MDMA	MDMA HCl Hydrate (50%) + Cellulose (35%)	0.96
P22	MDMA	MDMA HCl Hydrate (34%) + Cellulose (37%)	0.90
P22	MDMA	MDMA HCl Hydrate (43%) + Cellulose (34%)	0.92
P22	MDMA	MDMA HCl Hydrate (33%) + Cellulose (37%)	0.88
P23	MDMA	MDMA HCl Hydrate (68%) + Cellulose (30%)	0.97
P23	MDMA	MDMA HCl Hydrate (58%) + Cellulose (38%)	0.96
P23	MDMA	MDMA HCl Hydrate (65%) + Cellulose (32%)	0.97
P24	MDMA	MDMA HCl Hydrate (44%) + Cellulose (40%)	0.96
P24	MDMA	MDMA HCl Hydrate (55%) + Cellulose (42%)	0.97
P24	MDMA	MDMA HCl Hydrate (56%) + Cellulose (41%)	0.97
P25	MDMA	MDMA HCl Hydrate (47%) + Cellulose (29%) + Lactose (18%)	0.94
P25	MDMA	MDMA HCl Hydrate (47%) + Cellulose (28%) + Lactose (18%)	0.93
P25	MDMA	MDMA HCl Hydrate (50%) + Cellulose (28%) + Lactose (17%)	0.94
P26	MDMA	MDMA HCl Hydrate (32%) + Cellulose (64%)	0.96
P26	MDMA	MDMA HCl Hydrate (35%) + Cellulose (47%)	0.95
P26	MDMA	MDMA HCl Hydrate (42%) + Cellulose (54%)	0.96
P27	MDMA	MDMA HCl Hydrate (33%) + Cellulose (48%)	0.94
P27	MDMA	MDMA HCl Hydrate (36%) + Cellulose (48%)	0.95
P27	MDMA	MDMA HCl Hydrate (34%) + Cellulose (47%)	0.94
P28	MDMA	MDMA HCl Hydrate (48%) + Cellulose (49%)	0.97
P28	MDMA	MDMA HCl Hydrate (51%) + Cellulose (46%)	0.97
P28	MDMA	MDMA HCl Hydrate (49%) + Cellulose (48%)	0.97
P29	MDMA	MDMA HCl Hydrate (36%) + Lactose (39%)	0.91
P29	MDMA	MDMA HCl Hydrate (34%) + Lactose (38%)	0.89
P29	MDMA	MDMA HCl Hydrate (34%) + Lactose (38%)	0.90
P30	MDMA	No result	0.00
P30	MDMA	No result	0.00
P30	MDMA	No result	0.00
P31	MDMA	MDMA HCl Hydrate (58%) + Cellulose (40%)	0.98
P31	MDMA	MDMA HCl Hydrate (56%) + Cellulose (42%)	0.98

RESULTS ON MDMA MATRIX
(MDMA HCl·H20 only)

P31	MDMA	MDMA HCl Hydrate (58%) + Cellulose (40%)	0.98
P32	MDMA	MDMA HCl Hydrate (29%) + Cellulose (67%)	0.96
P32	MDMA	MDMA HCl Hydrate (32%) + Cellulose (64%)	0.96
P32	MDMA	MDMA HCl Hydrate (33%) + Cellulose (63%)	0.96
P33	MDMA	MDMA HCl Hydrate (44%) + Cellulose (48%)	0.91
P33	MDMA	MDMA HCl Hydrate (45%) + Cellulose (34%)	0.93
P33	MDMA	MDMA HCl Hydrate (51%) + Cellulose (43%)	0.94
P34	MDMA	MDMA HCl Hydrate (46%) + Cellulose (50%)	0.96
P34	MDMA	MDMA HCl Hydrate (50%) + Cellulose (47%)	0.97
P34	MDMA	MDMA HCl Hydrate (37%) + Cellulose (45%)	0.95
P35	MDMA	MDMA HCl Hydrate (60%) + Cellulose (36%)	0.97
P35	MDMA	MDMA HCl Hydrate (48%) + Cellulose (49%)	0.97
P35	MDMA	MDMA HCl Hydrate (61%) + Cellulose (36%)	0.97
P36	MDMA	MDMA HCl Hydrate (52%) + Cellulose (45%)	0.96
P36	MDMA	MDMA HCl Hydrate (49%) + Cellulose (48%)	0.97
P36	MDMA	MDMA HCl Hydrate (46%) + Cellulose (51%)	0.97
P37	MDMA	MDMA HCl Hydrate (52%) + Cellulose (44%)	0.96
P37	MDMA	MDMA HCl Hydrate (51%) + Cellulose (44%)	0.95
P37	MDMA	MDMA HCl Hydrate (52%) + Cellulose (43%)	0.95
P38	MDMA	MDMA HCl Hydrate (65%) + Cellulose (32%)	0.97
P38	MDMA	MDMA HCl Hydrate (60%) + Cellulose (37%)	0.97
P38	MDMA	MDMA HCl Hydrate (60%) + Cellulose (37%)	0.97
P39	MDMA	MDMA HCl Hydrate (31%) + Cellulose (65%)	0.96
P39	MDMA	MDMA HCl Hydrate (28%) + Cellulose (68%)	0.97
P39	MDMA	MDMA HCl Hydrate (28%) + Cellulose (68%)	0.96
P40	MDMA	MDMA HCl Hydrate (33%) + Cellulose (63%)	0.96
P40	MDMA	MDMA HCl Hydrate (30%) + Cellulose (66%)	0.96
P40	MDMA	MDMA HCl Hydrate (31%) + Cellulose (65%)	0.96
P101	2C-B	Cellulose (96%)	0.96
P101	2C-B	Cellulose (97%)	0.97
P101	2C-B	Cellulose (97%)	0.97
P102	4-MMC	No result	0.66
P102	4-MMC	No result	0.67
P102	4-MMC	No result	0.65
P104	2C-B	Cellulose (71%) + Magnesium Stearate (17%)	0.96
P104	2C-B	Cellulose (80%) + Magnesium Stearate (18%)	0.98
P104	2C-B	Cellulose (79%) + Magnesium Stearate (19%)	0.98
P105	2C-B	Cellulose (63%) + Magnesium Stearate (21%)	0.95
P105	2C-B	Cellulose (66%) + Magnesium Stearate (22%)	0.97
P105	2C-B	Cellulose (74%) + Magnesium Stearate (23%)	0.97
P106	2Br45DMPEA	Cellulose (73%)	0.95
P106	2Br45DMPEA	Cellulose (96%)	0.96
P106	2Br45DMPEA	Cellulose (77%)	0.96
P107	2Br45DMPEA	Cellulose (95%)	0.95
P107	2Br45DMPEA	Cellulose (96%)	0.96
P107	2Br45DMPEA	Cellulose (95%)	0.95
P108	FA	Cellulose (36%) + Magnesium Stearate (16%)	0.73
P108	FA	Cellulose (40%) + Magnesium Stearate (17%)	0.77
P108	FA	Cellulose (37%) + Magnesium Stearate (16%)	0.74
P109	FA	Cellulose (36%) + Magnesium Stearate (15%)	0.72
P109	FA	No result	0.70
P109	FA	Cellulose (33%) + Magnesium Stearate (14%)	0.70
P110	2C-B	Cellulose (97%)	0.97
P110	2C-B	Cellulose (95%)	0.95
P110	2C-B	Cellulose (96%)	0.96
P111	2C-B	Cellulose (97%)	0.97
P111	2C-B	Cellulose (97%)	0.97
P111	2C-B	Cellulose (97%)	0.97
P112	4-FMA	No result	0.70

P112	4-FMA	Mannitol (17%) + Cellulose (38%)	0.74
P112	4-FMA	Cellulose (37%)	0.78
P113	2Br45DMPEA	Cellulose (68%)	0.95
P113	2Br45DMPEA	Cellulose (96%)	0.96
P113	2Br45DMPEA	Cellulose (96%)	0.96
P114	2C-B	Cellulose (94%)	0.94
P114	2C-B	Cellulose (63%)	0.93
P114	2C-B	Cellulose (96%)	0.96
P115	pentylone	Cellulose (63%) + Magnesium Stearate (17%)	0.93
P115	pentylone	Cellulose (62%) + Magnesium Stearate (18%)	0.93
P115	pentylone	Cellulose (65%) + Magnesium Stearate (17%)	0.94
P116	2C-B	Cellulose (78%)	0.97
P116	2C-B	Cellulose (78%)	0.97
P116	2C-B	Cellulose (95%)	0.95
P117	FMA	Mannitol (18%) + Cellulose (34%)	0.72
P117	FMA	Mannitol (16%) + Cellulose (34%)	0.70
P117	FMA	Cellulose (31%)	0.75
P118	2C-B	Cellulose (98%)	0.98
P118	2C-B	Cellulose (80%)	0.97
P118	2C-B	Cellulose (98%)	0.98
P119	2C-B	Cellulose (97%)	0.97
P119	2C-B	Cellulose (97%)	0.97
P119	2C-B	Cellulose (97%)	0.97
P120	2C-B	Cellulose (93%)	0.93
P120	2C-B	Cellulose (66%)	0.93
P120	2C-B	Cellulose (93%)	0.93
P121	2C-B	Cellulose (97%)	0.97
P121	2C-B	Cellulose (97%)	0.97
P121	2C-B	Cellulose (97%)	0.97
P122	2Br45DMPEA	Cellulose (69%)	0.96
P122	2Br45DMPEA	Cellulose (96%)	0.96
P122	2Br45DMPEA	Cellulose (73%)	0.96
P123	FA	No result	0.68
P123	FA	No result	0.69
P123	FA	No result	0.68
P124	FA	Cellulose (36%) + Magnesium Stearate (17%)	0.73
P124	FA	Cellulose (35%) + Magnesium Stearate (16%)	0.72
P124	FA	Cellulose (32%) + Magnesium Stearate (17%)	0.70
P125	FMA	Cellulose (39%)	0.74
P125	FMA	Cellulose (42%)	0.76
P125	FMA	Cellulose (42%)	0.80
P126	4-FA	No result	0.38
P126	4-FA	No result	0.37
P126	4-FA	No result	0.39
P127	2C-B-fly	Cellulose (77%) + Magnesium Stearate (20%)	0.97
P127	2C-B-fly	Cellulose (77%) + Magnesium Stearate (19%)	0.96
P127	2C-B-fly	Cellulose (65%) + Magnesium Stearate (16%)	0.94
P128	FMA	Cellulose (47%)	0.79
P128	FMA	Cellulose (45%)	0.80
P128	FMA	Cellulose (49%)	0.84
P129	FA	Cellulose (93%)	0.93
P129	FA	Cellulose (72%)	0.93
P129	FA	Cellulose (95%)	0.95
P130	FMA	No result	0.50
P130	FMA	No result	0.43
P130	FMA	No result	0.54
P131	mCPP	Cellulose (66%)	0.93
P131	mCPP	Cellulose (94%)	0.94
P131	mCPP	Cellulose (74%)	0.94

RESULTS ON MDMA MATRIX
(MDMA HCl·H20 only)

P132	6-APB	No result	0.62
P132	6-APB	No result	0.57
P132	6-APB	No result	0.56
P133	4-FA	No result	0.34
P133	4-FA	No result	0.30
P133	4-FA	No result	0.20
T1	MDMA	MDMA HCl Hydrate (33%) + Cellulose (45%)	0.94
T1	MDMA	MDMA HCl Hydrate (31%) + Cellulose (48%)	0.93
T1	MDMA	MDMA HCl Hydrate (22%) + Cellulose (51%)	0.90
T2	MDMA	MDMA HCl Hydrate (32%) + Cellulose (53%)	0.97
T2	MDMA	MDMA HCl Hydrate (33%) + Cellulose (50%)	0.96
T2	MDMA	MDMA HCl Hydrate (26%) + Cellulose (58%)	0.97
T3	MDMA	MDMA HCl Hydrate (32%) + Cellulose (65%)	0.97
T3	MDMA	MDMA HCl Hydrate (28%) + Cellulose (61%)	0.98
T3	MDMA	MDMA HCl Hydrate (40%) + Cellulose (58%)	0.98
T4	MDMA	MDMA HCl Hydrate (37%) + Cellulose (59%)	0.96
T4	MDMA	MDMA HCl Hydrate (35%) + Cellulose (60%)	0.96
T4	MDMA	MDMA HCl Hydrate (36%) + Cellulose (50%)	0.96
T5	MDMA	MDMA HCl Hydrate (30%) + Cellulose (67%)	0.97
T5	MDMA	MDMA HCl Hydrate (25%) + Cellulose (72%)	0.97
T5	MDMA	MDMA HCl Hydrate (36%) + Cellulose (61%)	0.97
T6	MDMA	MDMA HCl Hydrate (46%) + Cellulose (39%)	0.96
T6	MDMA	MDMA HCl Hydrate (43%) + Cellulose (41%)	0.95
T6	MDMA	MDMA HCl Hydrate (49%) + Cellulose (36%)	0.96
T7	MDMA	MDMA HCl Hydrate (24%) + Cellulose (73%)	0.97
T7	MDMA	MDMA HCl Hydrate (19%) + Cellulose (77%)	0.96
T7	MDMA	MDMA HCl Hydrate (22%) + Cellulose (73%)	0.95
T8	MDMA	MDMA HCl Hydrate (37%) + Cellulose (45%) + Magnesium Stearate (15%)	0.97
T8	MDMA	MDMA HCl Hydrate (31%) + Cellulose (39%) + Magnesium Stearate (13%)	0.95
T8	MDMA	MDMA HCl Hydrate (35%) + Cellulose (38%) + Magnesium Stearate (14%)	0.96
T9	MDMA	MDMA HCl Hydrate (35%) + Cellulose (47%)	0.95
T9	MDMA	MDMA HCl Hydrate (36%) + Cellulose (48%)	0.95
T9	MDMA	MDMA HCl Hydrate (32%) + Cellulose (51%)	0.95
T10	MDMA	MDMA HCl Hydrate (39%) + Cellulose (46%)	0.96
T10	MDMA	MDMA HCl Hydrate (34%) + Cellulose (48%)	0.94
T10	MDMA	MDMA HCl Hydrate (34%) + Cellulose (48%)	0.94
T11	MDMA	MDMA HCl Hydrate (36%) + Cellulose (59%)	0.95
T11	MDMA	MDMA HCl Hydrate (42%) + Cellulose (54%)	0.96
T11	MDMA	MDMA HCl Hydrate (38%) + Cellulose (47%) + Magnesium Stearate (12%)	0.98
T12	MDMA	MDMA HCl Hydrate (44%) + Cellulose (40%)	0.95
T12	MDMA	MDMA HCl Hydrate (28%) + Cellulose (48%)	0.92
T12	MDMA	MDMA HCl Hydrate (37%) + Cellulose (45%)	0.94
T13	MDMA	MDMA HCl Hydrate (33%) + Cellulose (50%) + Magnesium Stearate (14%)	0.98
T13	MDMA	MDMA HCl Hydrate (35%) + Cellulose (48%) + Magnesium Stearate (14%)	0.97
T13	MDMA	MDMA HCl Hydrate (30%) + Cellulose (45%) + Magnesium Stearate (13%)	0.97
T14	MDMA	MDMA HCl Hydrate (30%) + Cellulose (41%) + Magnesium Stearate (13%)	0.95
T14	MDMA	MDMA HCl Hydrate (48%) + Cellulose (49%)	0.96
T14	MDMA	MDMA HCl Hydrate (25%) + Cellulose (45%) + Magnesium Stearate (13%)	0.95
T16	MDMA	MDMA HCl Hydrate (66%) + Cellulose (33%)	0.98
T16	MDMA	MDMA HCl Hydrate (60%) + Cellulose (29%)	0.98
T16	MDMA	MDMA HCl Hydrate (65%) + Cellulose (33%)	0.98
T17	MDMA	MDMA HCl Hydrate (38%) + Cellulose (44%)	0.94
T17	MDMA	MDMA HCl Hydrate (53%) + Cellulose (44%)	0.97
T17	MDMA	MDMA HCl Hydrate (42%) + Cellulose (53%)	0.95
T18	MDMA	MDMA HCl Hydrate (36%) + Cellulose (45%) + Magnesium Stearate (16%)	0.96
T18	MDMA	MDMA HCl Hydrate (27%) + Cellulose (41%) + Magnesium Stearate (15%)	0.96
T18	MDMA	MDMA HCl Hydrate (36%) + Cellulose (45%) + Magnesium Stearate (16%)	0.97
T19	MDMA	No result	0.67
T19	MDMA	No result	0.68

RESULTS ON MDMA MATRIX
(MDMA HCl·H20 only)

T19	MDMA	No result	0.69
T20	MDMA	MDMA HCl Hydrate (24%) + Cellulose (47%)	0.89
T20	MDMA	MDMA HCl Hydrate (25%) + Cellulose (46%)	0.90
T20	MDMA	MDMA HCl Hydrate (24%) + Cellulose (47%)	0.88
T21	MDMA	MDMA HCl Hydrate (48%) + Cellulose (35%)	0.96
T21	MDMA	MDMA HCl Hydrate (46%) + Cellulose (39%)	0.96
T21	MDMA	MDMA HCl Hydrate (47%) + Cellulose (37%)	0.95
T22	MDMA	MDMA HCl Hydrate (27%) + Cellulose (47%)	0.90
T22	MDMA	MDMA HCl Hydrate (23%) + Cellulose (46%)	0.89
T22	MDMA	MDMA HCl Hydrate (25%) + Cellulose (50%)	0.92
T23	MDMA	MDMA HCl Hydrate (30%) + Cellulose (43%) + Magnesium Stearate (14%)	0.97
T23	MDMA	MDMA HCl Hydrate (34%) + Cellulose (49%) + Magnesium Stearate (15%)	0.97
T23	MDMA	MDMA HCl Hydrate (38%) + Cellulose (46%) + Magnesium Stearate (13%)	0.97
T24	MDMA	MDMA HCl Hydrate (42%) + Cellulose (55%)	0.97
T24	MDMA	MDMA HCl Hydrate (37%) + Cellulose (60%)	0.97
T24	MDMA	MDMA HCl Hydrate (33%) + Cellulose (54%)	0.97
T25	MDMA	MDMA HCl Hydrate (34%) + Cellulose (26%) + Lactose (19%)	0.93
T25	MDMA	MDMA HCl Hydrate (38%) + Cellulose (28%) + Lactose (15%)	0.93
T25	MDMA	MDMA HCl Hydrate (35%) + Cellulose (28%) + Lactose (19%)	0.93
T26	MDMA	MDMA HCl Hydrate (42%) + Cellulose (56%)	0.98
T26	MDMA	MDMA HCl Hydrate (28%) + Cellulose (69%)	0.97
T26	MDMA	MDMA HCl Hydrate (28%) + Cellulose (69%)	0.97
T27	MDMA	MDMA HCl Hydrate (28%) + Cellulose (56%)	0.96
T27	MDMA	MDMA HCl Hydrate (31%) + Cellulose (47%)	0.93
T27	MDMA	MDMA HCl Hydrate (29%) + Cellulose (55%)	0.96
T28	MDMA	MDMA HCl Hydrate (36%) + Cellulose (61%)	0.98
T28	MDMA	MDMA HCl Hydrate (47%) + Cellulose (52%)	0.98
T28	MDMA	MDMA HCl Hydrate (37%) + Cellulose (60%)	0.97
T29	MDMA	MDMA HCl Hydrate (18%) + Lactose (56%)	0.90
T29	MDMA	MDMA HCl Hydrate (25%) + Lactose (53%)	0.93
T29	MDMA	MDMA HCl Hydrate (19%) + Lactose (49%)	0.86
T30	MDMA	No result	0.45
T30	MDMA	No result	0.59
T30	MDMA	No result	0.45
T31	MDMA	MDMA HCl Hydrate (32%) + Cellulose (66%)	0.97
T31	MDMA	MDMA HCl Hydrate (40%) + Cellulose (58%)	0.97
T31	MDMA	MDMA HCl Hydrate (31%) + Cellulose (66%)	0.97
T32	MDMA	MDMA HCl Hydrate (21%) + Cellulose (76%)	0.97
T32	MDMA	MDMA HCl Hydrate (19%) + Cellulose (77%)	0.97
T32	MDMA	MDMA HCl Hydrate (23%) + Cellulose (74%)	0.97
T33	MDMA	MDMA HCl Hydrate (26%) + Cellulose (56%) + Magnesium Stearate (15%)	0.97
T33	MDMA	MDMA HCl Hydrate (21%) + Cellulose (61%) + Magnesium Stearate (14%)	0.97
T33	MDMA	MDMA HCl Hydrate (22%) + Cellulose (52%) + Magnesium Stearate (13%)	0.97
T34	MDMA	MDMA HCl Hydrate (31%) + Cellulose (66%)	0.97
T34	MDMA	MDMA HCl Hydrate (39%) + Cellulose (58%)	0.97
T34	MDMA	MDMA HCl Hydrate (31%) + Cellulose (66%)	0.97
T35	MDMA	MDMA HCl Hydrate (51%) + Cellulose (47%)	0.98
T35	MDMA	MDMA HCl Hydrate (34%) + Cellulose (61%)	0.95
T35	MDMA	MDMA HCl Hydrate (50%) + Cellulose (47%)	0.98
T36	MDMA	MDMA HCl Hydrate (38%) + Cellulose (59%)	0.97
T36	MDMA	MDMA HCl Hydrate (37%) + Cellulose (61%)	0.98
T36	MDMA	MDMA HCl Hydrate (32%) + Cellulose (65%)	0.97
T37	MDMA	MDMA HCl Hydrate (42%) + Cellulose (53%)	0.96
T37	MDMA	MDMA HCl Hydrate (43%) + Cellulose (53%)	0.96
T37	MDMA	MDMA HCl Hydrate (47%) + Cellulose (49%)	0.96
T38	MDMA	MDMA HCl Hydrate (56%) + Cellulose (42%)	0.97
T38	MDMA	MDMA HCl Hydrate (50%) + Cellulose (39%)	0.97
T38	MDMA	MDMA HCl Hydrate (57%) + Cellulose (40%)	0.98
T39	MDMA	MDMA HCl Hydrate (19%) + Cellulose (78%)	0.98

RESULTS ON MDMA MATRIX
(MDMA HCl·H20 only)

T39	MDMA	MDMA HCl Hydrate (24%) + Cellulose (72%)	0.97
T39	MDMA	MDMA HCl Hydrate (22%) + Cellulose (75%)	0.97
T40	MDMA	MDMA HCl Hydrate (33%) + Cellulose (64%)	0.97
T40	MDMA	MDMA HCl Hydrate (17%) + Cellulose (80%)	0.97
T40	MDMA	MDMA HCl Hydrate (31%) + Cellulose (66%)	0.97
T101	2C-B	Cellulose (97%)	0.97
T101	2C-B	Cellulose (72%)	0.93
T101	2C-B	Cellulose (96%)	0.96
T102	4-MMC	Cellulose (48%)	0.79
T102	4-MMC	MDMA HCl Hydrate (9%) + Cellulose (44%)	0.76
T102	4-MMC	Cellulose (42%)	0.76
T104	2C-B	Cellulose (80%) + Magnesium Stearate (18%)	0.98
T104	2C-B	Cellulose (78%) + Magnesium Stearate (20%)	0.98
T104	2C-B	Cellulose (73%) + Magnesium Stearate (16%)	0.97
T105	2C-B	Cellulose (84%)	0.84
T105	2C-B	Cellulose (70%) + Magnesium Stearate (20%)	0.98
T105	2C-B	Cellulose (70%) + Magnesium Stearate (18%)	0.97
T106	2C-B isomer	Cellulose (96%)	0.96
T106	2C-B isomer	Cellulose (97%)	0.97
T106	2C-B isomer	Cellulose (96%)	0.96
T107	2C-B isomer	Cellulose (96%)	0.96
T107	2C-B isomer	Cellulose (96%)	0.96
T107	2C-B isomer	Cellulose (97%)	0.97
T108	FA	Cellulose (42%) + Magnesium Stearate (17%)	0.78
T108	FA	Mannitol (8%) + Cellulose (26%) + Magnesium Stearate (14%)	0.72
T108	FA	Mannitol (9%) + Cellulose (35%) + Magnesium Stearate (16%)	0.80
T109	FA	Mannitol (8%) + Cellulose (29%) + Magnesium Stearate (13%)	0.73
T109	FA	Cellulose (44%) + Magnesium Stearate (16%)	0.79
T109	FA	Mannitol (9%) + Cellulose (30%) + Magnesium Stearate (13%)	0.74
T110	2C-B	Cellulose (96%)	0.96
T110	2C-B	Cellulose (96%)	0.96
T110	2C-B	Cellulose (81%)	0.97
T111	2C-B	Cellulose (96%)	0.96
T111	2C-B	Cellulose (97%)	0.97
T111	2C-B	Cellulose (84%)	0.98
T112	4-FMA	Cellulose (53%)	0.84
T112	4-FMA	Mannitol (14%) + Cellulose (36%)	0.71
T112	4-FMA	Cellulose (51%)	0.84
T113	2C-B isomer	Cellulose (96%)	0.96
T113	2C-B isomer	Cellulose (96%)	0.96
T113	2C-B isomer	Cellulose (96%)	0.96
T114	2C-B	Cellulose (71%)	0.94
T114	2C-B	Cellulose (94%)	0.94
T114	2C-B	Cellulose (93%)	0.93
T115	pentylone	Cellulose (73%) + Magnesium Stearate (21%)	0.94
T115	pentylone	Cellulose (76%) + Magnesium Stearate (20%)	0.95
T115	pentylone	Cellulose (74%) + Magnesium Stearate (20%)	0.94
T116	2C-B	Cellulose (83%)	0.97
T116	2C-B	Cellulose (96%)	0.96
T116	2C-B	Cellulose (97%)	0.97
T117	FMA	Cellulose (55%)	0.86
T117	FMA	Cellulose (50%)	0.85
T117	FMA	Cellulose (33%)	0.75
T118	2C-B	Cellulose (98%)	0.98
T118	2C-B	Cellulose (98%)	0.98
T118	2C-B	Cellulose (96%)	0.96
T119	2C-B	Cellulose (97%)	0.97
T119	2C-B	Cellulose (83%)	0.97
T119	2C-B	Cellulose (97%)	0.97

T120	2C-B	Cellulose (94%)	0.94
T120	2C-B	Cellulose (92%)	0.92
T120	2C-B	Cellulose (75%)	0.95
T121	2C-B	Cellulose (97%)	0.97
T121	2C-B	Cellulose (97%)	0.97
T121	2C-B	Cellulose (97%)	0.97
T122	2C-B isomer	Cellulose (78%)	0.96
T122	2C-B isomer	Cellulose (75%)	0.96
T122	2C-B isomer	Cellulose (76%)	0.96
T123	FA	Cellulose (40%) + Magnesium Stearate (17%)	0.77
T123	FA	Mannitol (9%) + Cellulose (29%) + Magnesium Stearate (14%)	0.74
T123	FA	Mannitol (9%) + Cellulose (29%) + Magnesium Stearate (15%)	0.75
T124	FA	Cellulose (44%) + Magnesium Stearate (18%)	0.80
T124	FA	Cellulose (39%) + Magnesium Stearate (16%)	0.76
T124	FA	Cellulose (45%) + Magnesium Stearate (18%)	0.80
T125	FMA	Cellulose (97%)	0.97
T125	FMA	Cellulose (65%)	0.92
T125	FMA	Cellulose (97%)	0.97
T126	4-FA	Cellulose (32%)	0.72
T126	4-FA	Cellulose (40%)	0.78
T126	4-FA	Cellulose (32%)	0.74
T127	2C-B-fly	Cellulose (73%) + Magnesium Stearate (20%)	0.92
T127	2C-B-fly	Cellulose (68%) + Magnesium Stearate (17%)	0.95
T127	2C-B-fly	Cellulose (63%) + Magnesium Stearate (16%)	0.93
T128	FMA	Cellulose (96%)	0.96
T128	FMA	Cellulose (97%)	0.97
T128	FMA	Cellulose (95%)	0.95
T129	FA	Cellulose (97%)	0.97
T129	FA	Cellulose (98%)	0.98
T129	FA	Cellulose (98%)	0.98
T130	FMA	No result	0.55
T130	FMA	No result	0.52
T130	FMA	No result	0.60
T131	mCPP	Cellulose (93%)	0.93
T131	mCPP	Cellulose (94%)	0.94
T131	mCPP	Cellulose (68%)	0.94
T132	6-APB	No result	0.64
T132	6-APB	No result	0.66
T132	6-APB	No result	0.67
T133	4-FA	No result	0.45
T133	4-FA	No result	0.37
T133	4-FA	No result	0.39
T2_1	2C-B; dark green; clown	Cellulose (98%)	0.98
T2_2	2C-B; green; clown	Cellulose (86%)	0.99
T2_3	2C-B; green; Mario	Cellulose (84%)	0.97
T2_4	2C-B; light green; Moncler logo	Cellulose (99%)	0.99
T2_5	2C-B; pink; fox	Cellulose (99%)	0.99
T2_6	2C-B; pink; NASA logo	Cellulose (82%) + Magnesium Stearate (15%)	0.97
T2_7	2C-B; pink; Plusle	Cellulose (75%) + Magnesium Stearate (12%)	0.96
T2_8	2C-B; purple; Maybach logo	Cellulose (93%)	0.93
T2_9	2C-B; salmon; 2cb	Cellulose (82%)	0.97
T2_10	2C-B; salmon; griffin	Cellulose (97%)	0.97
T2_11	2C-B; salmon; Oreo logo	Cellulose (98%)	0.98
T2_12	2C-B; yellow; Pickachu	Cellulose (37%)	0.78
T2_13	2C-B; yellow; Plusle	Cellulose (79%)	0.96
T2_14	2C-B; yellow; robot	Cellulose (84%)	0.98
T2_15	4-FA; pink; Duplo	Mannitol (9%) + Cellulose (27%) + Magnesium Stearate (15%)	0.74
T2_16	amphetamine; blue; Bitcoin logo	Cellulose (96%)	0.96
T2_17	FMA; gray; Tomorrowland logo	Cellulose (40%)	0.80

RESULTS ON MDMA MATRIX
(MDMA HCl·H20 only)

T2_18	FMA; yellow; Tesla logo	Mannitol (18%) + Cellulose (37%)	0.74
T2_19	MDMA; black; Duracell	No result	0.00
T2_20	MDMA; black; Philipp Plein logo	No result	0.00
T2_21	MDMA; blue; FCBarcelona logo	MDMA HCl Hydrate (34%) + Cellulose (62%)	0.96
T2_22	MDMA; blue; Porsche logo	MDMA HCl Hydrate (37%) + Cellulose (60%)	0.97
T2_23	MDMA; blue; Punisher logo	MDMA HCl Hydrate (35%) + Cellulose (40%) + Magnesium Stearate (10%)	0.96
T2_24	MDMA; cream; Coca Cola logo	MDMA HCl Hydrate (27%) + Cellulose (69%)	0.97
T2_25	MDMA; cream; Flugel logo	MDMA HCl Hydrate (13%) + Cellulose (83%)	0.96
T2_26	MDMA; cream; Maserati logo	MDMA HCl Hydrate (61%) + Cellulose (37%)	0.98
T2_27	MDMA; cream; Rolex logo	MDMA HCl Hydrate (34%) + Cellulose (63%)	0.97
T2_28	MDMA; green; four leaf clover	MDMA HCl Hydrate (39%) + Cellulose (57%)	0.96
T2_29	MDMA; green; Heineken logo	MDMA HCl Hydrate (43%) + Cellulose (52%)	0.94
T2_30	MDMA; green; Jurrasic Park logo	MDMA HCl Hydrate (30%) + Cellulose (48%)	0.92
T2_31	MDMA; green; Nike sneaker	MDMA HCl Hydrate (36%) + Cellulose (59%)	0.96
T2_32	MDMA; grey; Jurrasic Park logo	MDMA HCl Hydrate (27%) + Cellulose (46%)	0.91
T2_33	MDMA; light blue; Philipp Plein logo	MDMA HCl Hydrate (38%) + Cellulose (44%)	0.94
T2_34	MDMA; light gray; granate	MDMA HCl Hydrate (29%) + Cellulose (53%)	0.95
T2_35	MDMA; light yellow; Casa de Papel logo	MDMA HCl Hydrate (22%) + Cellulose (76%)	0.98
T2_36	MDMA; light yellow; Philipp Plein logo	MDMA HCl Hydrate (29%) + Cellulose (49%)	0.94
T2_37	MDMA; light yellow; Punisher logo	MDMA HCl Hydrate (37%) + Cellulose (44%) + Magnesium Stearate (15%)	0.96
T2_38	MDMA; light yellow; Trump	MDMA HCl Hydrate (30%) + Cellulose (56%)	0.97
T2_39	MDMA; ocher; Mickey Mouse	MDMA HCl Hydrate (53%) + Cellulose (46%)	0.99
T2_40	MDMA; ocher; Mybrand logo	MDMA HCl Hydrate (13%) + Cellulose (56%) + Magnesium Stearate (14%)	0.95
T2_41	MDMA; orange; AUDI logo	MDMA HCl Hydrate (16%) + Cellulose (70%)	0.97
T2_42	MDMA; orange; Fanta logo	MDMA HCl Hydrate (18%) + Cellulose (64%)	0.94
T2_43	MDMA; orange; Michelin logo	MDMA HCl Hydrate (38%) + Cellulose (58%)	0.96
T2_44	MDMA; orange; Soundcloud logo	MDMA HCl Hydrate (29%) + Cellulose (68%)	0.98
T2_45	MDMA; pink; Casa de Papel logo	MDMA HCl Hydrate (16%) + Cellulose (75%)	0.98
T2_46	MDMA; pink; Skittles shape	MDMA HCl Hydrate (37%) + Cellulose (59%)	0.96
T2_47	MDMA; pink; Strawberry	MDMA HCl Hydrate (43%) + Cellulose (40%)	0.95
T2_48	MDMA; pink; Superman logo	MDMA HCl Hydrate (21%) + Cellulose (77%)	0.97
T2_49	MDMA; purple; Bugatti logo	MDMA HCl Hydrate (48%) + Cellulose (49%)	0.97
T2_50	MDMA; purple; shield	MDMA HCl Hydrate (15%) + Cellulose (79%)	0.94
T2_51	MDMA; purple; trump	MDMA HCl Hydrate (31%) + Cellulose (53%) + Magnesium Stearate (14%)	0.98
T2_52	MDMA; red; DJ	MDMA HCl Hydrate (59%) + Lactose (15%) + Cellulose (23%)	0.97
T2_53	MDMA; red; Nintendo	MDMA HCl Hydrate (53%) + Cellulose (45%)	0.98
T2_54	MDMA; red; Punisher logo	MDMA HCl Hydrate (13%) + Cellulose (68%) + Magnesium Stearate (15%)	0.96
T2_55	MDMA; red; Skittles shape	MDMA HCl Hydrate (32%) + Cellulose (38%) + Magnesium Stearate (13%)	0.95
T2_56	MDMA; red; WIFI logo	MDMA HCl Hydrate (36%) + Cellulose (47%)	0.95
T2_57	MDMA; salmon; Punisher logo	MDMA HCl Hydrate (34%) + Cellulose (40%) + Magnesium Stearate (12%)	0.95
T2_58	MDMA; white; smiley	MDMA HCl Hydrate (38%) + Cellulose (25%) + Lactose (16%)	0.93
T2_59	MDMA; yellow; Ducati logo	MDMA HCl Hydrate (53%) + Cellulose (45%)	0.98
T2_60	MDMA; yellow; Ghostbusters logo	MDMA HCl Hydrate (31%) + Cellulose (42%) + Magnesium Stearate (13%)	0.96
T2_61	MDMA; yellow; gold bar shape	No result	0.69
T2_62	MDMA; yellow; Minion shape	MDMA HCl Hydrate (27%) + Cellulose (55%)	0.95
T2_63	MDMA; yellow; Skittles shape	MDMA HCl Hydrate (42%) + Cellulose (55%)	0.97
T2_64	MDMA; yellow; Stewie shape	MDMA HCl Hydrate (40%) + Cellulose (56%)	0.96
T2_65	MDMA; yellow; Versace logo	MDMA HCl Hydrate (9%) + Cellulose (86%)	0.95
C1	Heroin (brown)	No result	0.31
C1	Heroin (brown)	No result	0.31
C1	Heroin (brown)	No result	0.32
C2	GHB (powder)	No result	0.49
C2	GHB (powder)	No result	0.48
C2	GHB (powder)	No result	0.48
C3	cocaine base	No result	0.19
C3	cocaine base	No result	0.19
C3	cocaine base	No result	0.18
C4	cocaine HCl	No result	0.39
C4	cocaine HCl	No result	0.39

RESULTS ON MDMA MATRIX
(MDMA HCl·H20 only)

C4	cocaine HCl	No result	0.39
C5	MDMA	MDMA HCl Hydrate (99%)	0.99
C5	MDMA	MDMA HCl Hydrate (100%)	1.00
C5	MDMA	MDMA HCl Hydrate (100%)	1.00
C6	cocaine base	No result	0.19
C6	cocaine base	No result	0.18
C6	cocaine base	No result	0.19
C7	cocaine HCl	No result	0.38
C7	cocaine HCl	No result	0.39
C7	cocaine HCl	No result	0.38
C8	amphetamine	No result	0.18
C8	amphetamine	No result	0.17
C8	amphetamine	No result	0.18
C9	amphetamine	No result	0.16
C9	amphetamine	No result	0.17
C9	amphetamine	No result	0.16
C10	GHB (powder)	No result	0.45
C10	GHB (powder)	No result	0.47
C10	GHB (powder)	No result	0.48
C11	GHB (powder)	No result	0.00
C11	GHB (powder)	No result	0.15
C11	GHB (powder)	No result	0.00
C12	GHB (liquid)	No result	0.00
C12	GHB (liquid)	No result	0.00
C12	GHB (liquid)	No result	0.00
C13	ketamine	No result	0.37
C13	ketamine	No result	0.36
C13	ketamine	No result	0.36
C14	methamphetamine	No result	0.29
C14	methamphetamine	No result	0.30
C14	methamphetamine	No result	0.31
C15	methamphetamine	No result	0.29
C15	methamphetamine	No result	0.31
C15	methamphetamine	No result	0.30
C16	MDMA	MDMA HCl Hydrate (99%)	0.99
C16	MDMA	MDMA HCl Hydrate (100%)	1.00
C16	MDMA	MDMA HCl Hydrate (99%)	0.99
C17	Heroin (brown)	No result	0.33
C17	Heroin (brown)	No result	0.54
C17	Heroin (brown)	No result	0.32
D1	2-CB	No result	0.09
D1	2-CB	No result	0.10
D1	2-CB	No result	0.11
D2	Caffeine	No result	0.36
D2	Caffeine	No result	0.34
D2	Caffeine	No result	0.37
D3	non-dairy creamer	Cellulose (37%) + Magnesium Stearate (29%)	0.86
D3	non-dairy creamer	Cellulose (39%) + Magnesium Stearate (29%)	0.87
D3	non-dairy creamer	Cellulose (37%) + Magnesium Stearate (29%)	0.86
D4	Ethylcathinon	No result	0.42
D4	Ethylcathinon	No result	0.44
D4	Ethylcathinon	No result	0.42
D5	N-ethylnorpentedron	No result	0.35
D5	N-ethylnorpentedron	No result	0.35
D5	N-ethylnorpentedron	No result	0.34
D6	MEC-CMC-CEC	No result	0.45
D6	MEC-CMC-CEC	No result	0.44
D6	MEC-CMC-CEC	No result	0.44
D7	FPM	No result	0.25

RESULTS ON MDMA MATRIX
(MDMA HCl·H20 only)

D7	FPM	No result	0.26
D7	FPM	No result	0.29
D8	levamisol	No result	0.28
D8	levamisol	No result	0.30
D8	levamisol	No result	0.29
D9	non-dairy creamer	Cellulose (37%) + Magnesium Stearate (32%)	0.88
D9	non-dairy creamer	Cellulose (37%) + Magnesium Stearate (33%)	0.88
D9	non-dairy creamer	Cellulose (38%) + Magnesium Stearate (31%)	0.88
D10	cocaine	No result	0.39
D10	cocaine	No result	0.40
D10	cocaine	No result	0.43
D11	2-CB	No result	0.12
D11	2-CB	No result	0.09
D11	2-CB	No result	0.12
D12	3-MEC	No result	0.44
D12	3-MEC	No result	0.44
D12	3-MEC	No result	0.27
D13	4-MEC	No result	0.39
D13	4-MEC	No result	0.23
D13	4-MEC	No result	0.38
D14	4-MEC	No result	0.41
D14	4-MEC	No result	0.25
D14	4-MEC	No result	0.39
D15	4-MEC	No result	0.40
D15	4-MEC	No result	0.39
D15	4-MEC	No result	0.38
D16	2-FMA	No result	0.44
D16	2-FMA	No result	0.44
D16	2-FMA	No result	0.43
D17	4-FA	No result	0.16
D17	4-FA	No result	0.14
D17	4-FA	No result	0.17
D18	4-MMC	No result	0.36
D18	4-MMC	No result	0.35
D18	4-MMC	No result	0.37
D19	3,4-dMMC	No result	0.00
D19	3,4-dMMC	No result	0.00
D19	3,4-dMMC	No result	0.00
D20	2-MMC	No result	0.47
D20	2-MMC	No result	0.45
D20	2-MMC	No result	0.47
D21	3-MMC	No result	0.51
D21	3-MMC	No result	0.50
D21	3-MMC	No result	0.50
D22	3-MMC	No result	0.50
D22	3-MMC	No result	0.50
D22	3-MMC	No result	0.51
D23	4-CMC	No result	0.36
D23	4-CMC	No result	0.37
D23	4-CMC	No result	0.38
D24	N-ethylpentylon	No result	0.00
D24	N-ethylpentylon	No result	0.00
D24	N-ethylpentylon	No result	0.00
D25	3-CMC	No result	0.39
D25	3-CMC	No result	0.40
D25	3-CMC	No result	0.40
D26	4-CMC	No result	0.36
D26	4-CMC	No result	0.34
D26	4-CMC	No result	0.36

D27	2-FMA	No result	0.42
D27	2-FMA	No result	0.41
D27	2-FMA	No result	0.42
D28	3,4-dMMC	No result	0.16
D28	3,4-dMMC	No result	0.13
D28	3,4-dMMC	No result	0.16
D29	5-APB	No result	0.39
D29	5-APB	No result	0.36
D29	5-APB	No result	0.38
D30	6-APB	No result	0.11
D30	6-APB	No result	0.14
D30	6-APB	No result	0.00
D31	DMMC	No result	0.17
D31	DMMC	No result	0.14
D31	DMMC	No result	0.15
D32	CMC	No result	0.33
D32	CMC	No result	0.33
D32	CMC	No result	0.33
D33	4-MC	No result	0.00
D33	4-MC	No result	0.00
D33	4-MC	No result	0.00
D34	Pentedrone	No result	0.37
D34	Pentedrone	No result	0.41
D34	Pentedrone	No result	0.37
D35	4-CMC	No result	0.37
D35	4-CMC	No result	0.36
D35	4-CMC	No result	0.38
D36	4-CMC	No result	0.43
D36	4-CMC	No result	0.44
D36	4-CMC	No result	0.42
D37	cocaine HCl	No result	0.41
D37	cocaine HCl	No result	0.41
D37	cocaine HCl	No result	0.41
D38	4-CEC	No result	0.37
D38	4-CEC	No result	0.37
D38	4-CEC	No result	0.38
N1	paracetamol	No result	0.16
N1	paracetamol	No result	0.15
N1	paracetamol	No result	0.15
N2	caffeine	No result	0.35
N2	caffeine	No result	0.34
N2	caffeine	No result	0.34
N3	levamisole	No result	0.29
N3	levamisole	No result	0.28
N3	levamisole	No result	0.28
N4	lidocaine	No result	0.24
N4	lidocaine	No result	0.34
N4	lidocaine	No result	0.28
N5	phenacetin	No result	0.29
N5	phenacetin	No result	0.33
N5	phenacetin	No result	0.32
N6	procaine	No result	0.62
N6	procaine	No result	0.62
N6	procaine	No result	0.61
N7	benzocaine	No result	0.00
N7	benzocaine	No result	0.00
N7	benzocaine	No result	0.00
N8	mannitol	Mannitol (99%)	0.99
N8	mannitol	Mannitol (99%)	0.99

N8	mannitol	Mannitol (99%)	0.99
N9	lactose	Lactose (99%)	0.99
N9	lactose	Lactose (99%)	0.99
N9	lactose	Lactose (99%)	0.99
N10	vitamin C	No result	0.26
N10	vitamin C	No result	0.35
N10	vitamin C	No result	0.37
N11	sugar (powdered)	No result	0.55
N11	sugar (powdered)	No result	0.55
N11	sugar (powdered)	No result	0.55
N12	glucose	No result	0.63
N12	glucose	No result	0.63
N12	glucose	No result	0.64
N13	boric acid	No result	0.20
N13	boric acid	No result	0.18
N13	boric acid	No result	0.17
N14	diltiazem	No result	0.37
N14	diltiazem	No result	0.35
N14	diltiazem	No result	0.35
N15	prometazine	No result	0.16
N15	prometazine	No result	0.15
N15	prometazine	No result	0.15
N16	non-dairy creamer	Magnesium Stearate (30%) + Cellulose (37%)	0.87
N16	non-dairy creamer	Cellulose (38%) + Magnesium Stearate (31%)	0.88
N16	non-dairy creamer	Cellulose (37%) + Magnesium Stearate (31%)	0.86
N17	wheat flour	Cellulose (34%)	0.82
N17	wheat flour	Cellulose (34%)	0.82
N17	wheat flour	Cellulose (34%)	0.83
N18	acetylsalicylic acid	No result	0.00
N18	acetylsalicylic acid	No result	0.00
N18	acetylsalicylic acid	No result	0.00
N19	ketamine	No result	0.38
N19	ketamine	No result	0.38
N19	ketamine	No result	0.37
N20	amphetamine	No result	0.16
N20	amphetamine	No result	0.17
N20	amphetamine	No result	0.14
N21	MDMA (powder)	MDMA HCl Hydrate (99%)	0.99
N21	MDMA (powder)	MDMA HCl Hydrate (99%)	0.99
N21	MDMA (powder)	MDMA HCl Hydrate (99%)	0.99
N22	methamphetamine	No result	0.30
N22	methamphetamine	No result	0.28
N22	methamphetamine	No result	0.29
N23	Heroin (white)	No result	0.21
N23	Heroin (white)	No result	0.00
N23	Heroin (white)	No result	0.21
N24	sildenafil citrate tablet, grinded	Cellulose (42%)	0.84
N24	sildenafil citrate tablet, grinded	Cellulose (47%)	0.87
N24	sildenafil citrate tablet, grinded	Cellulose (52%)	0.86
N25	oxazepam tablet, grinded	Lactose (66%)	0.94
N25	oxazepam tablet, grinded	Lactose (67%) + Cellulose (15%)	0.93
N25	oxazepam tablet, grinded	Lactose (63%) + Cellulose (15%)	0.92
N26	flunitrazepam tablet, grinded	Cellulose (45%) + Lactose (52%)	0.97
N26	flunitrazepam tablet, grinded	Cellulose (46%) + Lactose (49%)	0.95
N26	flunitrazepam tablet, grinded	Cellulose (49%) + Lactose (48%)	0.97
N27	mephedrone	No result	0.35
N27	mephedrone	No result	0.38
N27	mephedrone	No result	0.38
N28	4-FA tablet, grinded	No result	0.10

RESULTS ON MDMA MATRIX
(MDMA HCl·H20 only)

N28	4-FA tablet, grinded	No result	0.11
N28	4-FA tablet, grinded	No result	0.11
N29	paracetamol :caffein, 1:1	No result	0.00
N29	paracetamol :caffein, 1:1	No result	0.19
N29	paracetamol :caffein, 1:1	No result	0.08
N30	levamisole:lidocaine, 1:1	No result	0.34
N30	levamisole:lidocaine, 1:1	No result	0.35
N30	levamisole:lidocaine, 1:1	No result	0.34
N31	levamisole:paracetamol:lidocaine, 1:1:1	No result	0.25
N31	levamisole:paracetamol:lidocaine, 1:1:1	No result	0.19
N31	levamisole:paracetamol:lidocaine, 1:1:1	No result	0.21
N32	levamisole:phenacetin, 1:1	No result	0.25
N32	levamisole:phenacetin, 1:1	No result	0.26
N32	levamisole:phenacetin, 1:1	No result	0.26
N33	phenacetin:lidocaine, 1:1	No result	0.28
N33	phenacetin:lidocaine, 1:1	No result	0.28
N33	phenacetin:lidocaine, 1:1	No result	0.27
N34	phenacetin:procaine, 1:1	No result	0.53
N34	phenacetin:procaine, 1:1	No result	0.52
N34	phenacetin:procaine, 1:1	No result	0.52
N35	levamisole:phenacetin:procaine, 1:1:1	No result	0.48
N35	levamisole:phenacetin:procaine, 1:1:1	No result	0.47
N35	levamisole:phenacetin:procaine, 1:1:1	No result	0.47
N36	paracetamol:phenacetin, 1:1	No result	0.24
N36	paracetamol:phenacetin, 1:1	No result	0.23
N36	paracetamol:phenacetin, 1:1	No result	0.24
N37	diazepam tablet 10 mg, grinded	Cellulose (53%) + Lactose (38%)	0.91
N37	diazepam tablet 10 mg, grinded	Cellulose (36%) + Lactose (35%)	0.88
N37	diazepam tablet 10 mg, grinded	Cellulose (37%) + Lactose (36%)	0.89
N38	methylphenidate 10 mg tablet, grinded	Cellulose (34%)	0.82
N38	methylphenidate 10 mg tablet, grinded	Cellulose (37%) + Lactose (19%)	0.83
N38	methylphenidate 10 mg tablet, grinded	Cellulose (39%) + Lactose (18%)	0.85
N39	smartshop blend mix caffeine, lactose, mannitol	Mannitol (27%) + Lactose (35%) + Magnesium Stearate (20%)	0.95
N39	smartshop blend mix caffeine, lactose, mannitol	Mannitol (27%) + Lactose (40%) + Magnesium Stearate (17%)	0.96
N39	smartshop blend mix caffeine, lactose, mannitol	Mannitol (33%) + Lactose (41%) + Magnesium Stearate (21%)	0.95
N40	inositol	Inositol (99%)	0.99
N40	inositol	Inositol (89%)	0.99
N40	inositol	Inositol (99%)	0.99
PAM1	caffeine + levamisol	No result	0.70
PAM1	caffeine + levamisol	MDMA HCl Hydrate (11%) + Mannitol (38%)	0.71
PAM1	caffeine + levamisol	MDMA HCl Hydrate (11%) + Mannitol (39%)	0.72
PAM2	cocaine	No result	0.31
PAM2	cocaine	No result	0.30
PAM2	cocaine	No result	0.32
PAM3	cocaine	No result	0.41
PAM3	cocaine	No result	0.41
PAM3	cocaine	No result	0.42
PAM6	cocaine	No result	0.19
PAM6	cocaine	No result	0.21
PAM6	cocaine	No result	0.22
PAM7	cocaine + procaine	No result	0.42
PAM7	cocaine + procaine	No result	0.43
PAM7	cocaine + procaine	No result	0.43
PAM8	cocaine	No result	0.43
PAM8	cocaine	No result	0.41
PAM8	cocaine	No result	0.40
PAM9	cocaine + lidocaine + procaine + levamisole	No result	0.69
PAM9	cocaine + lidocaine + procaine + levamisole	No result	0.67
PAM9	cocaine + lidocaine + procaine + levamisole	No result	0.68

RESULTS ON MDMA MATRIX
(MDMA HCl·H20 only)

PAM11	MDMA	MDMA HCl Hydrate (98%)	0.98
PAM11	MDMA	MDMA HCl Hydrate (98%)	0.98
PAM11	MDMA	MDMA HCl Hydrate (99%)	0.99
PAM12	cocaine	No result	0.40
PAM12	cocaine	No result	0.40
PAM12	cocaine	No result	0.42
PAM13	cocaine	No result	0.39
PAM13	cocaine	No result	0.39
PAM13	cocaine	No result	0.40
PAM14	negatief	No result	0.46
PAM14	negatief	No result	0.46
PAM14	negatief	No result	0.46
PAM15	ketamine	No result	0.32
PAM15	ketamine	No result	0.37
PAM15	ketamine	No result	0.38
PAM16	cocaine + procaine + phenacetin	No result	0.57
PAM16	cocaine + procaine + phenacetin	No result	0.59
PAM16	cocaine + procaine + phenacetin	No result	0.59
PAM17	cocaine + lidocaine + phenacetin + levamisole	No result	0.21
PAM17	cocaine + lidocaine + phenacetin + levamisole	No result	0.23
PAM17	cocaine + lidocaine + phenacetin + levamisole	No result	0.23
PAM18	ketamine	No result	0.39
PAM18	ketamine	No result	0.38
PAM18	ketamine	No result	0.38
PAM19	ketamine	No result	0.43
PAM19	ketamine	No result	0.44
PAM19	ketamine	No result	0.42
PAM20	cocaine + phenacetin	No result	0.19
PAM20	cocaine + phenacetin	No result	0.16
PAM20	cocaine + phenacetin	No result	0.21
PAM21	cocaine	No result	0.40
PAM21	cocaine	No result	0.40
PAM21	cocaine	No result	0.39
PAM22	cocaine	No result	0.39
PAM22	cocaine	No result	0.41
PAM22	cocaine	No result	0.41
PAM23	cocaine	No result	0.15
PAM23	cocaine	No result	0.30
PAM23	cocaine	No result	0.30
PAM25	negatief	No result	0.10
PAM25	negatief	No result	0.07
PAM25	negatief	No result	0.11
PAM26	ketamine	No result	0.38
PAM26	ketamine	No result	0.15
PAM26	ketamine	No result	0.41
PAM27	cocaine + levamisole	No result	0.30
PAM27	cocaine + levamisole	No result	0.18
PAM27	cocaine + levamisole	No result	0.32
PAM28	cocaine	No result	0.41
PAM28	cocaine	No result	0.41
PAM28	cocaine	No result	0.40
PAM29	amphetamine	No result	0.18
PAM29	amphetamine	No result	0.18
PAM29	amphetamine	No result	0.19
PAM31	cocaine + levamisole	No result	0.38
PAM31	cocaine + levamisole	No result	0.41
PAM31	cocaine + levamisole	No result	0.41
PAM32	cocaine + lidocaine + caffeine + phenacetin + levamisole	No result	0.31
PAM32	cocaine + lidocaine + caffeine + phenacetin + levamisole	No result	0.32

RESULTS ON MDMA MATRIX
(MDMA HCl·H20 only)

PAM32	cocaine + lidocaine + caffeine + phenacetin + levamisole	No result	0.30
PAM33	cocaine + levamisole	No result	0.29
PAM33	cocaine + levamisole	No result	0.30
PAM33	cocaine + levamisole	No result	0.31
PAM35	cocaine + levamisole	No result	0.36
PAM35	cocaine + levamisole	No result	0.36
PAM35	cocaine + levamisole	No result	0.36
PAM36	cocaine + phenacetin + levamisole	No result	0.26
PAM36	cocaine + phenacetin + levamisole	No result	0.28
PAM36	cocaine + phenacetin + levamisole	No result	0.26
PAM37	paracetamol + caffeine	No result	0.20
PAM37	paracetamol + caffeine	No result	0.15
PAM37	paracetamol + caffeine	No result	0.20
PAM39	ketamine	No result	0.35
PAM39	ketamine	No result	0.37
PAM39	ketamine	No result	0.38
PAM40	lidocaine	No result	0.40
PAM40	lidocaine	No result	0.42
PAM40	lidocaine	No result	0.43
PAM41	cocaine + lidocaine + levamisole	No result	0.33
PAM41	cocaine + lidocaine + levamisole	No result	0.32
PAM41	cocaine + lidocaine + levamisole	No result	0.33
PAM42	cocaine	No result	0.42
PAM42	cocaine	No result	0.41
PAM42	cocaine	No result	0.40
PAM43	MDMA	MDMA HCl Hydrate (99%)	0.99
PAM43	MDMA	MDMA HCl Hydrate (99%)	0.99
PAM43	MDMA	MDMA HCl Hydrate (99%)	0.99
PAM44	cocaine	No result	0.40
PAM44	cocaine	No result	0.40
PAM44	cocaine	No result	0.41
PAM45	phenacetin	No result	0.34
PAM45	phenacetin	No result	0.33
PAM45	phenacetin	No result	0.34
PAM46	amphetamine	No result	0.32
PAM46	amphetamine	No result	0.34
PAM46	amphetamine	No result	0.32
PAM47	cocaine	No result	0.40
PAM47	cocaine	No result	0.39
PAM47	cocaine	No result	0.41
PAM48	phenacetin	Cellulose (33%)	0.80
PAM48	phenacetin	Cellulose (32%)	0.79
PAM48	phenacetin	Cellulose (32%)	0.80
PAM49	cocaine + phenacetin + levamisole	No result	0.21
PAM49	cocaine + phenacetin + levamisole	No result	0.21
PAM49	cocaine + phenacetin + levamisole	No result	0.21
PAM50	cocaine	No result	0.31
PAM50	cocaine	No result	0.19
PAM50	cocaine	No result	0.31
PAM51	cocaine + levamisole	No result	0.19
PAM51	cocaine + levamisole	No result	0.19
PAM51	cocaine + levamisole	No result	0.21
PAM52	cocaine + levamisole	No result	0.31
PAM52	cocaine + levamisole	No result	0.31
PAM52	cocaine + levamisole	No result	0.31
PAM53	negatief	No result	0.33
PAM53	negatief	No result	0.24
PAM53	negatief	No result	0.32
PAM56	MDMA	MDMA HCl Hydrate (99%)	0.99

RESULTS ON MDMA MATRIX
(MDMA HCl·H20 only)

PAM56	MDMA	MDMA HCl Hydrate (99%)	0.99
PAM56	MDMA	MDMA HCl Hydrate (71%)	0.94
PAM57	cocaine	No result	0.40
PAM57	cocaine	No result	0.40
PAM57	cocaine	No result	0.39
PAM58	cocaine	No result	0.32
PAM58	cocaine	No result	0.30
PAM58	cocaine	No result	0.32
PAM59	negatief	No result	0.00
PAM59	negatief	No result	0.00
PAM59	negatief	No result	0.00
PAM60	MDMA	MDMA HCl Hydrate (97%)	0.97
PAM60	MDMA	MDMA HCl Hydrate (99%)	0.99
PAM60	MDMA	MDMA HCl Hydrate (97%)	0.97
PAM61	amphetamine	No result	0.27
PAM61	amphetamine	No result	0.32
PAM62	cocaine + levamisole	No result	0.40
PAM62	cocaine + levamisole	No result	0.41
PAM62	cocaine + levamisole	No result	0.25
PAM63	cocaine + lidocaine + caffeine + phenacetin + levamisole	No result	0.32
PAM63	cocaine + lidocaine + caffeine + phenacetin + levamisole	No result	0.30
PAM63	cocaine + lidocaine + caffeine + phenacetin + levamisole	No result	0.37
PAM64	cocaine	No result	0.31
PAM64	cocaine	No result	0.29
PAM64	cocaine	No result	0.30
PAM65	levamisol	No result	0.28
PAM65	levamisol	No result	0.28
PAM65	levamisol	No result	0.29
PAM66	lidocaine	No result	0.39
PAM66	lidocaine	No result	0.41
PAM66	lidocaine	No result	0.41
PAM67	levamisol	No result	0.31
PAM67	levamisol	No result	0.30
PAM67	levamisol	No result	0.31
PAM68	cocaine + phenacetin + levamisole	No result	0.19
PAM68	cocaine + phenacetin + levamisole	No result	0.22
PAM68	cocaine + phenacetin + levamisole	No result	0.18
PAM69	cocaine	No result	0.30
PAM69	cocaine	No result	0.31
PAM69	cocaine	No result	0.16
PAM71	negatief	No result	0.25
PAM71	negatief	No result	0.25
PAM71	negatief	No result	0.27
PAM72	cocaine + caffeine + phenacetin	No result	0.18
PAM72	cocaine + caffeine + phenacetin	No result	0.17
PAM72	cocaine + caffeine + phenacetin	No result	0.17
PAM73	cocaine + caffeine	No result	0.19
PAM73	cocaine + caffeine	No result	0.32
PAM73	cocaine + caffeine	No result	0.32
PAM74	MDMA	MDMA HCl Hydrate (99%)	0.99
PAM74	MDMA	MDMA HCl Hydrate (99%)	0.99
PAM74	MDMA	MDMA HCl Hydrate (98%)	0.98
PAM75	cocaine + levamisole	No result	0.42
PAM75	cocaine + levamisole	No result	0.43
PAM75	cocaine + levamisole	No result	0.41
PAM76	caffeine	No result	0.63
PAM76	caffeine	No result	0.57
PAM76	caffeine	No result	0.54
PAM77	phenacetin, caffeine, levamisol	No result	0.44

PAM77	phenacetin, caffeine, levamisol	No result	0.45
PAM77	phenacetin, caffeine, levamisol	No result	0.44
PAM78	cocaine	No result	0.47
PAM78	cocaine	No result	0.55
PAM78	cocaine	No result	0.48
PAM79	mitrazapine	Cellulose (33%) + Magnesium Stearate (14%)	0.74
PAM79	mitrazapine	Cellulose (35%) + Magnesium Stearate (15%)	0.76
PAM79	mitrazapine	Cellulose (36%) + Magnesium Stearate (13%)	0.75
PAM80	cocaine	No result	0.40
PAM80	cocaine	No result	0.41
PAM80	cocaine	No result	0.42
PAM81	cocaine + levamisole	Mannitol (97%)	0.97
PAM81	cocaine + levamisole	Mannitol (61%)	0.89
PAM81	cocaine + levamisole	Mannitol (55%)	0.87
PAM82	tetracaine	No result	0.34
PAM82	tetracaine	No result	0.34
PAM82	tetracaine	No result	0.34
PAM83	cocaine	No result	0.39
PAM83	cocaine	No result	0.40
PAM83	cocaine	No result	0.42
PAM84	ketamine	No result	0.39
PAM84	ketamine	No result	0.39
PAM84	ketamine	No result	0.38
PAM85	phenacetin	No result	0.32
PAM85	phenacetin	No result	0.33
PAM85	phenacetin	No result	0.34
PAM86	cocaine + levamisole	Mannitol (55%)	0.87
PAM86	cocaine + levamisole	Mannitol (56%)	0.88
PAM86	cocaine + levamisole	Mannitol (60%)	0.90
PAM87	cocaine + levamisole	No result	0.45
PAM87	cocaine + levamisole	No result	0.44
PAM87	cocaine + levamisole	No result	0.42
PAM88	cocaine + lidocaine + procaine + levamisole	No result	0.59
PAM88	cocaine + lidocaine + procaine + levamisole	No result	0.61
PAM88	cocaine + lidocaine + procaine + levamisole	No result	0.59
PAM89	cocaine	No result	0.35
PAM89	cocaine	No result	0.33
PAM89	cocaine	No result	0.28
PAM90	negatief	No result	0.25
PAM90	negatief	No result	0.24
PAM90	negatief	No result	0.17
PAM91	cocaine + levamisole	No result	0.42
PAM91	cocaine + levamisole	No result	0.41
PAM91	cocaine + levamisole	No result	0.42
PAM92	amphetamine	No result	0.38
PAM92	amphetamine	No result	0.38
PAM92	amphetamine	No result	0.34
PAM93	ketamine	No result	0.39
PAM93	ketamine	No result	0.38
PAM93	ketamine	No result	0.40
PAM94	cocaine + phenacetin	No result	0.16
PAM94	cocaine + phenacetin	No result	0.17
PAM94	cocaine + phenacetin	No result	0.18
PAM95	phenacetin	No result	0.33
PAM95	phenacetin	No result	0.34
PAM95	phenacetin	No result	0.34
PAM96	cocaine	No result	0.31
PAM96	cocaine	No result	0.31
PAM96	cocaine	No result	0.30

PAM97	cocaine + levamisole	No result	0.19
PAM97	cocaine + levamisole	No result	0.20
PAM97	cocaine + levamisole	No result	0.19
PAM98	levamisol	No result	0.29
PAM98	levamisol	No result	0.29
PAM98	levamisol	No result	0.30
PAM99	cocaine + levamisole	No result	0.40
PAM99	cocaine + levamisole	No result	0.40
PAM99	cocaine + levamisole	No result	0.41
PAM101	cocaine + lidocaine + phenacetin + levamisole	No result	0.39
PAM101	cocaine + lidocaine + phenacetin + levamisole	No result	0.36
PAM101	cocaine + lidocaine + phenacetin + levamisole	No result	0.37
PAM102	cocaine + lidocaine	No result	0.41
PAM102	cocaine + lidocaine	No result	0.40
PAM102	cocaine + lidocaine	No result	0.42
PAM103	cocaine + lidocaine + tetracaine + levamisole	No result	0.40
PAM103	cocaine + lidocaine + tetracaine + levamisole	No result	0.40
PAM103	cocaine + lidocaine + tetracaine + levamisole	No result	0.40
PAM104	cocaine + lidocaine + levamisole	No result	0.40
PAM104	cocaine + lidocaine + levamisole	No result	0.41
PAM104	cocaine + lidocaine + levamisole	No result	0.41
PAM105	amphetamine	No result	0.23
PAM105	amphetamine	No result	0.23
PAM105	amphetamine	No result	0.24
PAM106	ketamine	No result	0.37
PAM106	ketamine	No result	0.37
PAM106	ketamine	No result	0.38
PAM107	cocaine + lidocaine	No result	0.41
PAM107	cocaine + lidocaine	No result	0.41
PAM107	cocaine + lidocaine	No result	0.41
PAM108	cocaine + phenacetin	No result	0.30
PAM108	cocaine + phenacetin	No result	0.31
PAM108	cocaine + phenacetin	No result	0.31
PAM109	cocaine	No result	0.31
PAM109	cocaine	No result	0.30
PAM109	cocaine	No result	0.31
PAM110	metamphetamine	No result	0.35
PAM110	metamphetamine	No result	0.36
PAM110	metamphetamine	No result	0.32
PAM111	ketamine	No result	0.41
PAM111	ketamine	No result	0.38
PAM111	ketamine	No result	0.38
PAM112	cocaine + lidocaine	No result	0.31
PAM112	cocaine + lidocaine	No result	0.30
PAM112	cocaine + lidocaine	No result	0.32
PAM113	cocaine	No result	0.41
PAM113	cocaine	No result	0.40
PAM113	cocaine	No result	0.41
PAM114	cocaine + phenacetin + levamisole	No result	0.40
PAM114	cocaine + phenacetin + levamisole	No result	0.41
PAM114	cocaine + phenacetin + levamisole	No result	0.39
PAM115	MDMA	MDMA HCl Hydrate (99%)	0.99
PAM115	MDMA	MDMA HCl Hydrate (99%)	0.99
PAM115	MDMA	MDMA HCl Hydrate (100%)	1.00
PAM116	MDMA	MDMA HCl Hydrate (99%)	0.99
PAM116	MDMA	MDMA HCl Hydrate (99%)	0.99
PAM116	MDMA	MDMA HCl Hydrate (99%)	0.99
PAM117	MDMA	MDMA HCl Hydrate (99%)	0.99
PAM117	MDMA	MDMA HCl Hydrate (100%)	1.00

RESULTS ON MDMA MATRIX
(MDMA HCl·H20 only)

PAM117	MDMA	MDMA HCl Hydrate (100%)	1.00
PAM118	cocaine	No result	0.29
PAM118	cocaine	No result	0.30
PAM118	cocaine	No result	0.30
PAM119	cocaine	No result	0.41
PAM119	cocaine	No result	0.42
PAM119	cocaine	No result	0.42
PAM120	amphetamine	No result	0.34
PAM120	amphetamine	No result	0.37
PAM120	amphetamine	No result	0.32
PAM121	cocaine	No result	0.41
PAM121	cocaine	No result	0.43
PAM121	cocaine	No result	0.43
PAM122	cocaine + levamisole	No result	0.42
PAM122	cocaine + levamisole	No result	0.41
PAM122	cocaine + levamisole	No result	0.43
PAM123	cocaine + lidocaine + benzocaine + phenacetin	No result	0.61
PAM123	cocaine + lidocaine + benzocaine + phenacetin	No result	0.63
PAM123	cocaine + lidocaine + benzocaine + phenacetin	No result	0.60
PAM124	cocaine + levamisole	No result	0.30
PAM124	cocaine + levamisole	No result	0.31
PAM124	cocaine + levamisole	No result	0.31
PAM125	amphetamine	No result	0.37
PAM125	amphetamine	No result	0.38
PAM125	amphetamine	No result	0.36
PAM126	amphetamine	No result	0.30
PAM126	amphetamine	No result	0.30
PAM126	amphetamine	No result	0.26
PAM127	cocaine + phenacetin + levamisole	No result	0.22
PAM127	cocaine + phenacetin + levamisole	No result	0.24
PAM127	cocaine + phenacetin + levamisole	No result	0.23
PAM128	cocaine + levamisole	No result	0.42
PAM128	cocaine + levamisole	No result	0.40
PAM128	cocaine + levamisole	No result	0.41
PAM129	cocaine	No result	0.40
PAM129	cocaine	No result	0.41
PAM129	cocaine	No result	0.42
PAM131	cocaine + caffeine + levamisole	No result	0.43
PAM131	cocaine + caffeine + levamisole	No result	0.40
PAM131	cocaine + caffeine + levamisole	No result	0.44
PAM132	mannitol	Mannitol (99%)	0.99
PAM132	mannitol	Mannitol (99%)	0.99
PAM132	mannitol	Mannitol (99%)	0.99
PAM133	cocaine + lidocaine	No result	0.41
PAM133	cocaine + lidocaine	No result	0.42
PAM133	cocaine + lidocaine	No result	0.42
PAM134	cocaine	No result	0.41
PAM134	cocaine	No result	0.41
PAM134	cocaine	No result	0.41
PAM135	cocaine + phenacetin	No result	0.21
PAM135	cocaine + phenacetin	No result	0.20
PAM135	cocaine + phenacetin	No result	0.21
PAM136	amphetamine	No result	0.35
PAM136	amphetamine	No result	0.33
PAM136	amphetamine	No result	0.35
PAM137	cocaine	No result	0.42
PAM137	cocaine	No result	0.42
PAM137	cocaine	No result	0.41
PAM139	metamphetamine	No result	0.32

RESULTS ON MDMA MATRIX
(MDMA HCl·H20 only)

PAM139	metamphetamine	No result	0.33
PAM139	metamphetamine	No result	0.34
PAM140	ketamine	No result	0.38
PAM140	ketamine	No result	0.37
PAM140	ketamine	No result	0.37
PAM141	MDMA	MDMA HCl Hydrate (99%)	0.99
PAM141	MDMA	MDMA HCl Hydrate (70%)	0.98
PAM141	MDMA	MDMA HCl Hydrate (99%)	0.99
PAM142	negatief	Mannitol (99%)	0.99
PAM142	negatief	Mannitol (99%)	0.99
PAM142	negatief	Mannitol (99%)	0.99
PAM143	THC	No result	0.18
PAM143	THC	No result	0.15
PAM143	THC	No result	0.00
PAM144	cocaine	No result	0.42
PAM144	cocaine	No result	0.40
PAM144	cocaine	No result	0.42
PAM145	amphetamine	No result	0.36
PAM145	amphetamine	No result	0.38
PAM145	amphetamine	No result	0.35
PAM146	cocaine + lidocaine + procaine + tetracaine + phenacetin + levamisole	No result	0.29
PAM146	cocaine + lidocaine + procaine + tetracaine + phenacetin + levamisole	No result	0.26
PAM146	cocaine + lidocaine + procaine + tetracaine + phenacetin + levamisole	No result	0.35
PAM147	cocaine + lidocaine	No result	0.41
PAM147	cocaine + lidocaine	No result	0.41
PAM147	cocaine + lidocaine	No result	0.43
PAM148	amphetamine	No result	0.31
PAM148	amphetamine	No result	0.32
PAM148	amphetamine	No result	0.32
PAM149	cocaine + lidocaine	No result	0.48
PAM149	cocaine + lidocaine	No result	0.50
PAM149	cocaine + lidocaine	No result	0.51
PAM150	cocaine + caffeine + levamisole	No result	0.39
PAM150	cocaine + caffeine + levamisole	No result	0.37
PAM150	cocaine + caffeine + levamisole	No result	0.40
PAM151	cocaine	MDMA HCl Hydrate (10%) + Mannitol (44%)	0.75
PAM151	cocaine	MDMA HCl Hydrate (12%) + Mannitol (39%)	0.73
PAM151	cocaine	MDMA HCl Hydrate (10%) + Mannitol (43%)	0.75
PAM152	cocaine	No result	0.17
PAM152	cocaine	No result	0.30
PAM152	cocaine	No result	0.31
PAM153	cocaine + tetracaine + phenacetin + levamisole	No result	0.41
PAM153	cocaine + tetracaine + phenacetin + levamisole	No result	0.41
PAM153	cocaine + tetracaine + phenacetin + levamisole	No result	0.40
PAM154	ketamine	No result	0.38
PAM154	ketamine	No result	0.39
PAM154	ketamine	No result	0.38
PAM156	ketamine	No result	0.40
PAM156	ketamine	No result	0.39
PAM156	ketamine	No result	0.39
PAM157	cocaine + tetracaine + caffeine + levamisole	No result	0.52
PAM157	cocaine + tetracaine + caffeine + levamisole	No result	0.58
PAM157	cocaine + tetracaine + caffeine + levamisole	No result	0.58
PAM158	cocaine + lidocaine + procaine + levamisole	No result	0.31
PAM158	cocaine + lidocaine + procaine + levamisole	No result	0.32
PAM158	cocaine + lidocaine + procaine + levamisole	No result	0.33
PAM159	cocaine + lidocaine + phenacetin	MDMA HCl Hydrate (12%) + Mannitol (35%)	0.71
PAM159	cocaine + lidocaine + phenacetin	No result	0.67
PAM159	cocaine + lidocaine + phenacetin	MDMA HCl Hydrate (9%) + Mannitol (42%)	0.74

RESULTS ON MDMA MATRIX
(MDMA HCl·H20 only)

PAM160	cocaine + lidocaine + phenacetin	No result	0.66
PAM160	cocaine + lidocaine + phenacetin	No result	0.64
PAM160	cocaine + lidocaine + phenacetin	No result	0.64
PAM161	cocaine	No result	0.30
PAM161	cocaine	No result	0.31
PAM161	cocaine	No result	0.18
PAM162	cocaine + caffeine + levamisole	No result	0.58
PAM162	cocaine + caffeine + levamisole	No result	0.62
PAM162	cocaine + caffeine + levamisole	No result	0.59
PAM163	cocaine	No result	0.15
PAM163	cocaine	No result	0.16
PAM163	cocaine	No result	0.14
PAM165	cocaine + procaine + phenacetin + levamisole	No result	0.23
PAM165	cocaine + procaine + phenacetin + levamisole	No result	0.21
PAM165	cocaine + procaine + phenacetin + levamisole	No result	0.21
PAM166	ketamine	No result	0.41
PAM166	ketamine	No result	0.40
PAM166	ketamine	No result	0.38
PAM167	cocaine	No result	0.49
PAM167	cocaine	No result	0.48
PAM167	cocaine	No result	0.50
PAM168	cocaine	No result	0.48
PAM168	cocaine	No result	0.51
PAM168	cocaine	No result	0.51
PAM169	cocaine	Mannitol (33%)	0.75
PAM169	cocaine	Mannitol (30%)	0.73
PAM169	cocaine	Mannitol (32%)	0.72
PAM170	levamisol	No result	0.19
PAM170	levamisol	No result	0.20
PAM170	levamisol	No result	0.20
PAM171	cocaine	No result	0.29
PAM171	cocaine	No result	0.30
PAM171	cocaine	No result	0.29
PAM172	cocaine + levamisole	No result	0.40
PAM172	cocaine + levamisole	No result	0.39
PAM172	cocaine + levamisole	No result	0.39
PAM174	THC	No result	0.00
PAM174	THC	No result	0.00
PAM174	THC	No result	0.00
PAM175	cocaine + levamisole	No result	0.51
PAM175	cocaine + levamisole	No result	0.53
PAM175	cocaine + levamisole	No result	0.51
PAM176	cocaine + phenacetin	No result	0.17
PAM176	cocaine + phenacetin	No result	0.18
PAM176	cocaine + phenacetin	No result	0.16
PAM177	cocaine + levamisole	No result	0.42
PAM177	cocaine + levamisole	No result	0.40
PAM177	cocaine + levamisole	No result	0.40
PAM179	cocaine + procaine	No result	0.40
PAM179	cocaine + procaine	No result	0.42
PAM179	cocaine + procaine	No result	0.41
PAM180	MDMA	MDMA HCl Hydrate (99%)	0.99
PAM180	MDMA	MDMA HCl Hydrate (100%)	1.00
PAM180	MDMA	MDMA HCl Hydrate (99%)	0.99
PAM181	cocaine + caffeine	No result	0.40
PAM181	cocaine + caffeine	No result	0.40
PAM181	cocaine + caffeine	No result	0.40
PAM182	amphetamine	No result	0.27
PAM182	amphetamine	No result	0.32

RESULTS ON MDMA MATRIX
(MDMA HCl·H20 only)

PAM182	amphetamine	No result	0.29
PAM183	cocaine + levamisole	Inositol (36%)	0.77
PAM183	cocaine + levamisole	Inositol (30%) + Mannitol (26%)	0.76
PAM183	cocaine + levamisole	Inositol (24%) + Mannitol (27%)	0.72
PAM184	3-MMC	No result	0.50
PAM184	3-MMC	No result	0.50
PAM184	3-MMC	No result	0.51
PAM185	MDMA	MDMA HCl Hydrate (99%)	0.99
PAM185	MDMA	MDMA HCl Hydrate (99%)	0.99
PAM185	MDMA	MDMA HCl Hydrate (100%)	1.00
PAM186	cocaine + levamisole	No result	0.52
PAM186	cocaine + levamisole	No result	0.49
PAM186	cocaine + levamisole	No result	0.51
PAM188	cocaine	No result	0.40
PAM188	cocaine	No result	0.41
PAM188	cocaine	No result	0.40
PAM189	MDMA	MDMA HCl Hydrate (99%)	0.99
PAM189	MDMA	MDMA HCl Hydrate (95%)	0.95
PAM189	MDMA	MDMA HCl Hydrate (98%)	0.98
PAM190	cocaine + levamisole	No result	0.22
PAM190	cocaine + levamisole	No result	0.20
PAM190	cocaine + levamisole	No result	0.19
PAM191	ketamine	No result	0.38
PAM191	ketamine	No result	0.38
PAM191	ketamine	No result	0.38
PAM192	amphetamine	No result	0.30
PAM192	amphetamine	No result	0.16
PAM192	amphetamine	No result	0.18
PAM193	ketamine	No result	0.36
PAM193	ketamine	No result	0.37
PAM193	ketamine	No result	0.39
PAM194	cocaine + levamisole	No result	0.16
PAM194	cocaine + levamisole	No result	0.18
PAM194	cocaine + levamisole	No result	0.20
PAM195	cocaine	No result	0.16
PAM195	cocaine	No result	0.19
PAM195	cocaine	No result	0.19
PAM196	lidocaine	No result	0.43
PAM196	lidocaine	No result	0.43
PAM196	lidocaine	No result	0.42
PAM197	amphetamine	No result	0.34
PAM197	amphetamine	No result	0.33
PAM197	amphetamine	No result	0.33
PAM199	cocaine + phenacetin + levamisole	No result	0.34
PAM199	cocaine + phenacetin + levamisole	No result	0.36
PAM199	cocaine + phenacetin + levamisole	No result	0.35
PAM200	MDMA	MDMA HCl Hydrate (99%)	0.99
PAM200	MDMA	MDMA HCl Hydrate (100%)	1.00
PAM200	MDMA	MDMA HCl Hydrate (99%)	0.99
